# Design, Synthesis, and Evaluation of Camptothecin-Based Antibody–Drug Conjugates with High Hydrophilicity and Structural Stability

**DOI:** 10.3390/molecules30071398

**Published:** 2025-03-21

**Authors:** Tingyu Xiong, Jiyu Jin, Dongliang Liu, Chen Jin

**Affiliations:** 1 College of Chemistry and Chemical Engineering, Donghua University, 2999 North Renmin Road, Shanghai 201620, China; 2 Shanghai Tekanbio Pharm-Tech Co., Ltd., Room 4001, Floor 4, Unit 3, Building 8, No. 160, Basheng Road, China (Shanghai) Pilot Free Trade Zone, Shanghai 200120, China; 3 Shanghai Engineering Research Center of Molecular Therapeutics and New Drug Development, School of Chemistry and Molecular Engineering, East China Normal University, 3663 North Zhongshan Road, Shanghai 200062, China

**Keywords:** antibody–drug conjugates, polysarcosine, polyethylene glycol, topoisomerase I inhibitor

## Abstract

In this study, we constructed a linear antibody–drug conjugate (ADC), 7300-LP1003, by coupling the camptothecin derivative 095 to a linker through an ether bond. In vitro enzyme experiments indicated that LP1003 releases 095 through the action of tissue cathepsin B. Therefore, we introduced lysine pairs with different water-soluble substituents to further modify the linker and synthesized side-chain ADCs 7300-LP3004 and 7300-LP2004, modified by polysarcosine and polyethylene glycol, respectively. In vitro experiments showed that, after incubation at 55 °C in phosphate-buffered saline for 48 h, 7300-LP3004 aggregation was 45.24%, which was significantly lower than that of 7300-LP1003 (77.14%). Cell cytotoxicity assays demonstrated that the side-chain ADCs, 7300-LP3004 and 7300-LP2004, exhibited significantly higher activity (IC50 values of 39.74 nM and 32.17 nM, respectively) compared to the linear ADC and 7300-Deruxtecan (IC50 of 186.5 nM and 124.5 nM, respectively). In the subcutaneous model of SHP-77 NOD scid gamma mice, when the ADC dose was 5 mg/kg, 7300-LP3004 showed the highest tumor inhibition rate with a tumor growth inhibition (TGI) of 106.09%, which was superior to that of the positive control 7300-Deruxtecan, which had a TGI of 103.95%. In conclusion, 7300-LP3004 demonstrated strong antitumor activity and high physicochemical stability, highlighting the need for further research and development of ADC drugs.

## 1. Introduction

Antibody–drug conjugates (ADCs) are innovative drugs with targeted therapeutic properties and that have emerged as promising anticancer therapies [1]. ADCs combine the specificity of monoclonal antibodies with the potency of cytotoxic payloads, enabling selective drug delivery to tumor cells and overcoming the limitations of traditional chemotherapeutic drugs, such as non-specific toxicity and low drug accumulation in tumors [2,3]. IMMU-132 (sacituzumab govitecan) was the first ADC to use the camptothecin derivative SN38 as a payload and was the first drug to successfully treat triple-negative breast cancer [4]. Later, ENHERTU (Trastuzumab, T-DXd) was the first ADC to use the camptothecin derivative Dxd as a payload, successfully achieving targeted therapy against HER2 and showing significant efficacy in HER2-positive breast cancer [5]. However, despite the clinical success of ADCs, their potential is limited by challenges such as complex pharmacokinetics, insufficient tumor targeting, inadequate payload release, and resistance mechanisms [6].

The linker in ADCs plays a crucial role because premature release of the payload into the bloodstream can cause severe toxic reactions and diminish therapeutic efficacy [7]. An ideal linker should exhibit high stability, water solubility, and specific release properties. The linker should remain stable in the bloodstream to prevent premature payload release and should be cleaved or hydrolyzed under specific conditions within tumor cells to release the cytotoxic payload and exert therapeutic effects [8]. Song et al. [9] reported that using ether bonds as linkers in ADCs with SN38, a camptothecin derivative, enhanced payload stability while ensuring efficient drug release within target tumor cells. Polyethylene glycol (PEG), one of the most commonly used hydrophilic polymers, is widely used in ADCs to regulate water solubility owing to its simple structure and stable spatial conformation [10,11,12,13]. However, PEG also has inherent drawbacks, such as non-biodegradability, potential allergic reactions in patients, and accelerated blood clearance [14]. The amide bond (-CONR-) and flexible structure of PSar enhance its hydrophilicity [15,16,17,18,19,20]. Meng et al. [6] reported that replacing PEG with PSar in ADCs resulted in higher hydrophilicity, less aggregation, and greater stability, thus improving the hydrophilicity of hydrophobic camptothecin derivatives, enhancing ADC stability, reducing aggregation, and significantly increasing the antitumor activity of the payload [14,21]

In this study, we replaced SN38 with the more potent camptothecin compound 095 as a payload for an antibody–drug conjugate. This payload was conjugated to the PEG-modified Val-Ala-PAB linker via an ether bond, and antibody 7300 targeting B7H3 was utilized to successfully prepare the linear ADC 7300-LP1003. Subsequently, we introduced side-chain modifications with PEG and PSar to obtain the side-chain ADCs 7300-LP2004 and 7300-LP3004. Research demonstrated that the PSar-modified side-chain ADC 7300-LP3004 exhibited stronger water solubility, higher stability, and enhanced in vitro and in vivo antitumor activities than the positive control compound. This design and screening process identified promising ADC candidates with optimized linker–payload configurations.

## 2. Results

### 2.1. Construction and Synthesis of Linker–Payload (LP)

**Figure 1:**
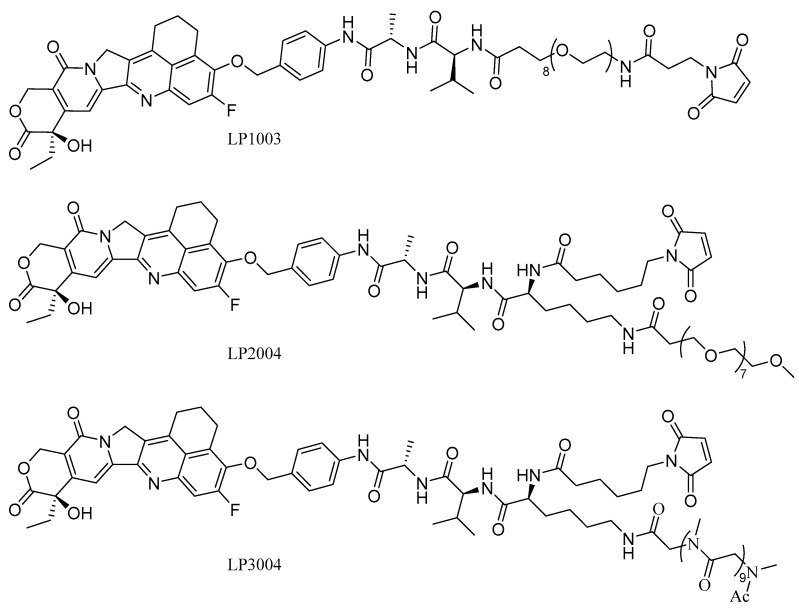
The structure of the LP used in this study.

We showed through in vitro cytotoxin experiments that the semi-inhibitory concentration (IC50) of 095 was better than Dxd (14.39 nM and 59.56 nM, respectively) (Figure S1), indicating that 095 exhibited higher activity than Dxd on SHP-77 cells in vitro. Therefore, we selected the camptothecin derivative 095 as the payload. To improve LP hydrophilicity, we designed and synthesized three sets of hydrophilic linker–payloads: a PSar-modified branched-link agent, a polyethylene glycol-modified straight-link agent, and a polyethylene glycol-modified branched-link agent (Figure 1). Detailed experiments and data are provided in the Supplementary Materials.

**Scheme 1:**
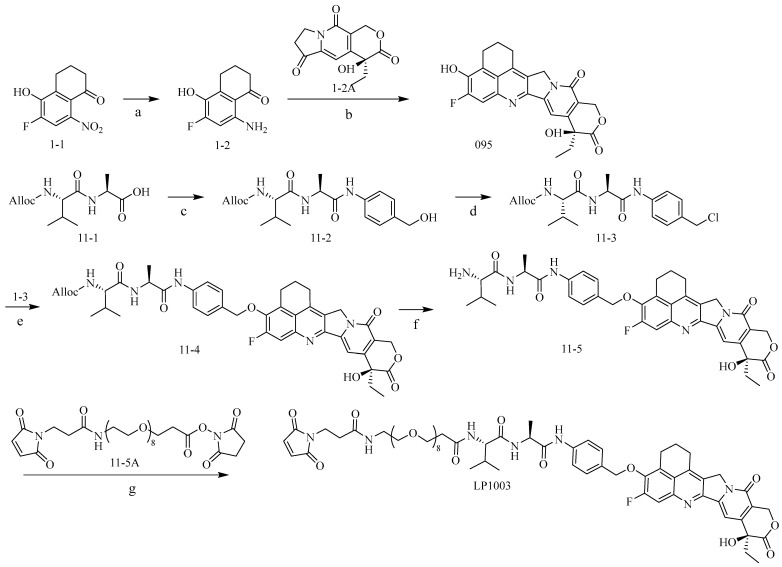
Synthesis of LP1003. Conditions: (a) Ni, MeOH, R.T, 99.23%; (b) Toluene/AcOH, PPTS, 110 °C, 83.16%; (c) EEDQ, THF, R.T, 63.49%; (d) THF, SOCl_2_, R.T, 94.07%; (e) DMF, Pempidine, 50 °C, 38.84%; (f) Pd(PPh_3_)_4_, DMF, R.T, 78.05%; (g) ACN, TEA, R.T, 43.87%.

First, a PEG-modified straight-chain linker payload (LP1003) was synthesized. As shown in Scheme 1, commercially available compound **1-1** was reduced with Raney nickel in methanol at room temperature to obtain compound **1-2** with a yield of over 95%. Compound **1-2** was subjected to the Friedlander reaction with commercially available **1-2A** at 110 °C, and the crude product was purified using DCM/MTBE/ethanol beating to obtain compound **095** with a yield of more than 80%. Commercially available **11-1** and (4-aminophenyl) methanol were condensed in a THF/EEDQ mixture. The resulting crude product was purified using THF/MTBE beating to obtain compound **11-2** with a yield of 63.49%. Compound **11-2** reacted with dichlorosulfoxide in THF to obtain compound **11-3** in quantitative yield, which was directly used in the next reaction without purification. At 55 °C, compound **11-3** and compound **095** underwent a substitution reaction to obtain compound **11-4** with a yield of 38.84%. The allyloxycarbonyl protection group of compound **11-4** was removed using Pd(PPh_3_)_4_ and piperidine in DMF, and compound **11-5** was purified using column chromatography with a yield of 78.05%. Under the influence of TEA, compound **11-5** was reacted with commercially available **11-5A** to obtain purified LP1003 with a yield of 43.87%.

**Scheme 2:**
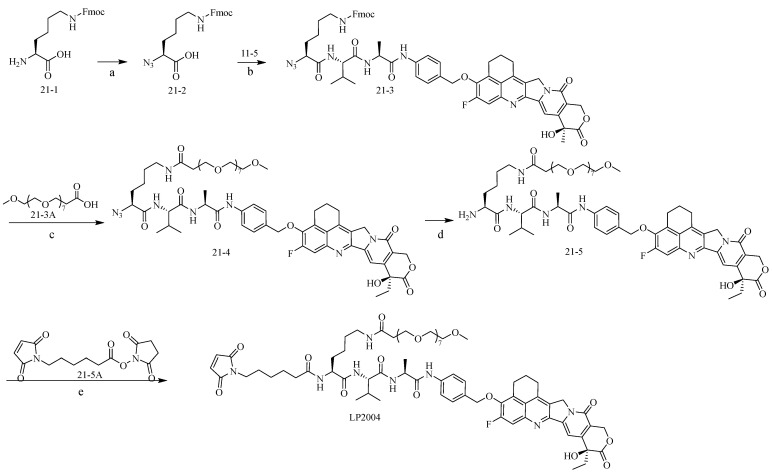
Synthesis of LP2004. Conditions: (a) MeOH/DCM/H_2_O, K_2_CO_3_, CuSO_4_, R.T, 56.07%; (b) DMF, TEA, HATU, R.T, 99.57%; (c) DMF, DEA, HATU, DIPET, R.T, 57.78%; (d) PBS (pH 6.0), P(CH_3_)_3_, 0 °C, 47.66%; (e) DMF/ACN, TEA, R.T, 53.53%.

We further synthesized a PEG-modified branched-link LP2004 as shown in Scheme 2. Under alkaline conditions, compound **21-1** reacted with 1H-imidazole-1-azide hydrochloride. The pH was adjusted to 3 using 4 M HCl, and compound **21-2** was extracted with EA, resulting in a yield of 56.07%. In the presence of TEA in DMF, compound **11-5** and compound **21-2** were reacted to obtain compound **21-3** with a yield of 99.57%. In the presence of DEA, the Fmoc-protective groups of compound **21-3** were removed to obtain the corresponding amino compounds. Under the action of HATU, the amino compounds reacted with **21-3A** to obtain compound **21-4** with a yield of 57.78%. Compound **21-4** was reduced by P(CH_3_)_3_ to obtain compound **21-5** with a yield of 47.66%. Under the influence of TEA, compound **21-5** was reacted with commercially available **21-5A** to obtain purified LP2004 with a yield of 53.53%.

**Scheme 3:**
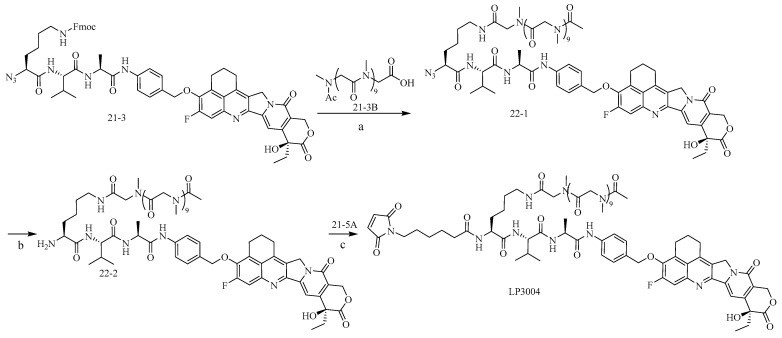
Synthesis of LP3004. Conditions: (a) DMF, DEA, HATU, DIPET, R.T, 85.91%; (b) PBS (pH 6.0), P(CH_3_)_3_, 0 °C, 30.49%; (c) DMF/ACN, TEA, R.T, 46.33%.

Finally, we synthesized a PSar-modified branched-link LP3004 as shown in Scheme 3. In the presence of DEA, the Fmoc protective groups of compound **21-3** were removed to obtain the corresponding amino compound. Under the action of HATU, the amino compound reacted with **21-3B** to obtain compound **22-1** with a yield of 85.91%. Compound 22-1 was reduced by P(CH_3_)_3_ to obtain compound **22-2** with a yield of 30.49%. Compound **22-2** was substituted with commercially available **21-5A** to obtain LP3004 with a yield of 46.33%.

### 2.2. The Physical Characterization of LP2004 and LP3004

**Figure 2:**
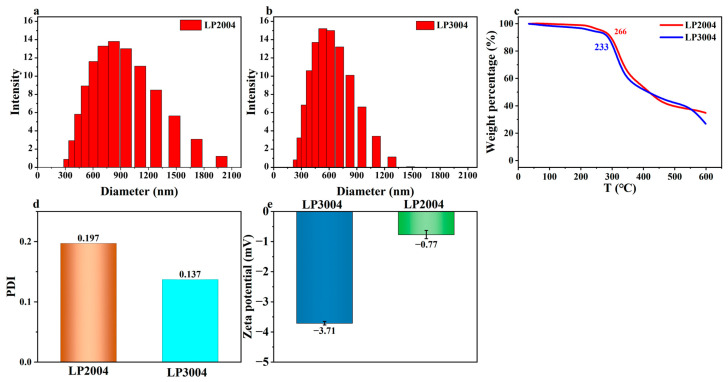
Zetasizer Nano ZS results of LP2004 (**a**) and LP3004 (**b**). (**c**) TGA curves of LP3004 and LP2004. (**d**) PDI of LP2004 and LP3004. (**e**) Zeta potential data of LP3004 and LP2004.

A higher polydispersity index (PDI) value and a lower absolute zeta potential indicate a wider particle size distribution [22], which leads to increased instability of the drug molecules and a higher tendency for aggregation [23]. The mean intensity and PDI of LP3004 were 607.3 nm and 0.137, respectively (Figure 2b,d). In contrast, the mean intensity and PDI of LP2004 increased to 890.4 nm and 0.197 (Figure 2a,d). PSar modification significantly reduced the PDI when the mc-Lys-Val-Ala-PAB tripeptide sequence was used, and the absolute zeta potential of LP was significantly increased after modification with hydrophilic PSar. The zeta potential of LP3004 and LP2004 was −3.71 mV and −0.77 mV, respectively (Figure 2e). The thermal decomposition temperatures (95%) of LP3004 and LP2004 measured using thermogravimetric analysis were 233 °C and 266 °C, respectively (Figure 2c). Our results demonstrated that LP3004 exhibited greater stability than LP2004 when the same tripeptide sequence was used.

### 2.3. In Vitro Evaluation of Linker–Payload

**Figure 3:**
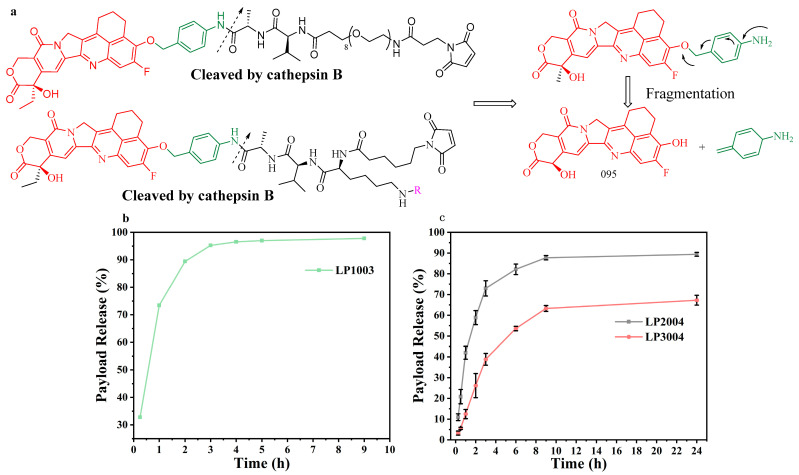
(**a**) LP1003, LP2004, or LP3004 was efficiently cleaved by cathepsin B in the buffer (R = CO-CH_2_-PSar10-Ac or CO-CH_2_-PEG8-Me). (**b**) Cathepsin B-mediated release of free drugs from LP1003. (**c**) Cathepsin B-mediated release of free drugs from LP2004 and LP3004.

To investigate the release of payload from the LP, we conducted an in vitro enzymatic experiment. High-performance liquid chromatography (HPLC) was used to monitor the rate of cathepsin B cleavage and payload release, while liquid chromatography–mass spectrometry (LC-MS) was employed to confirm the identity of the cleavage product. First, we evaluated the effects of LP1003 on human placental cathepsin B-mediated cleavage (Figure 3a). The release of 095 from LP1003 was monitored using HPLC at 37 °C. After incubation with cathepsin B for 9 h, 97.77% of 095 was released (Figure 3b), confirming the feasibility of linking 095 to the linker via ether bonds. The release of 095 from LP3004 and LP2004 was also monitored. LP3004 and LP2004 were incubated in a cathepsin B solution. The results demonstrated that the amount of 095 increased over time (Figure 3c and Figure S2a–e). Both LP3004 and LP2004 released 095 through cathepsin B-mediated cleavage. We observed faster bond cleavage in LP3004 compared to LP2004, with 095 release rates of 89% and 67%, respectively (Figure 3c).

### 2.4. Bioconjugation and Physicochemical Characterization of ADCs

**Figure 4:**
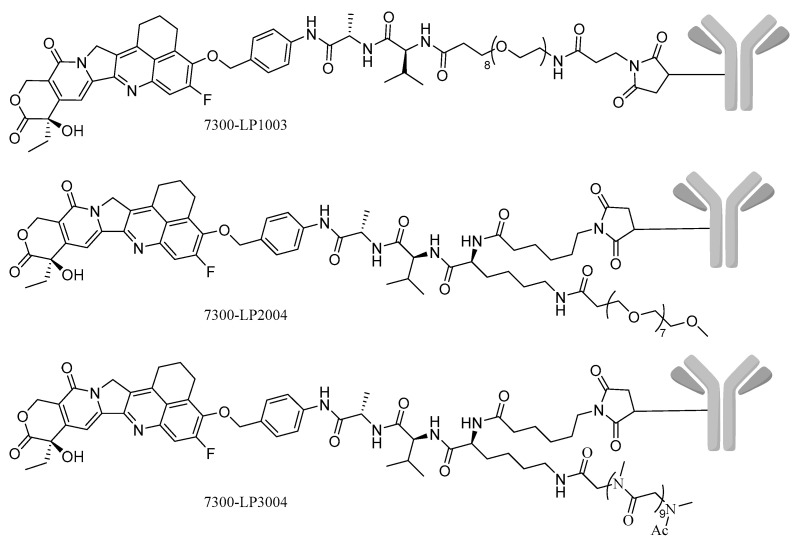
The structure of ADCs.

**Figure 5:**
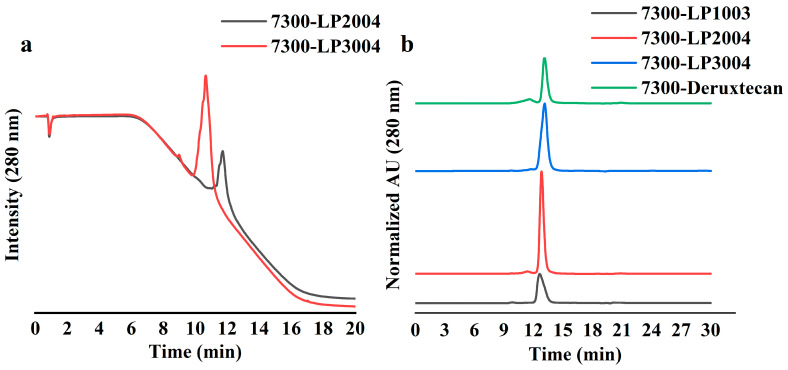
(**a**) HIC of 7300-LP2004 and 7300-LP3004. (**b**) SEC of DAR8 ADCs.

We employed the biological coupling methods to conjugate LP1003, LP2004, and LP3004 with the reduced 7300 antibody, resulting in 7300-LP1003, 7300-LP2004, and 7300-LP3004, respectively, with a drug–antibody ratio (DAR) of 8 (Figure 4). Antibody 7300 is an lgG1 monoclonal antibody targeting B7-H3. The specific coupling methods are detailed in the Supporting Materials. Analytical characterization using LC-MS confirmed the efficiency of the conjugation (Figure S3a–d). Bioconjugation yields exceeded 75% for 7300-LP3004, 7300-LP2004, and 7300-LP1003, and were over 60% for 7300-Deruxtecan. To evaluate the impact of chemical modifications with PEG and PSar on hydrophobicity, hydrophobic interaction chromatography (HIC) was performed under physiological conditions in phosphate buffer at pH 7.4 (Figure 5a). We observed a significant increase in the HIC retention time for LP2004 compared to LP3004, confirming that LP3004 exhibited greater hydrophilicity than LP2004 when conjugated with the same antibodies.

This result indicates that the use of PSar in ADC formation can reduce ADC hydrophobicity. This feature is advantageous for the construction of ADCs, particularly high-drug–antibody ratio, as increased ADC hydrophobicity generates aggregation [24].

Size exclusion chromatography (SEC) analysis revealed that 7300-LP3004 existed as a 95%+ monomer with no detectable aggregation. In the formulation, 7300-Deruxtecan exhibited 17% aggregation, while 7300-LP2004 and 7300-LP1003 showed 2.3% and 3.7 aggregation, respectively (Figure 5b).

### 2.5. 7300-LP3004 Exhibited Strong Stability In Vitro

**Figure 6:**
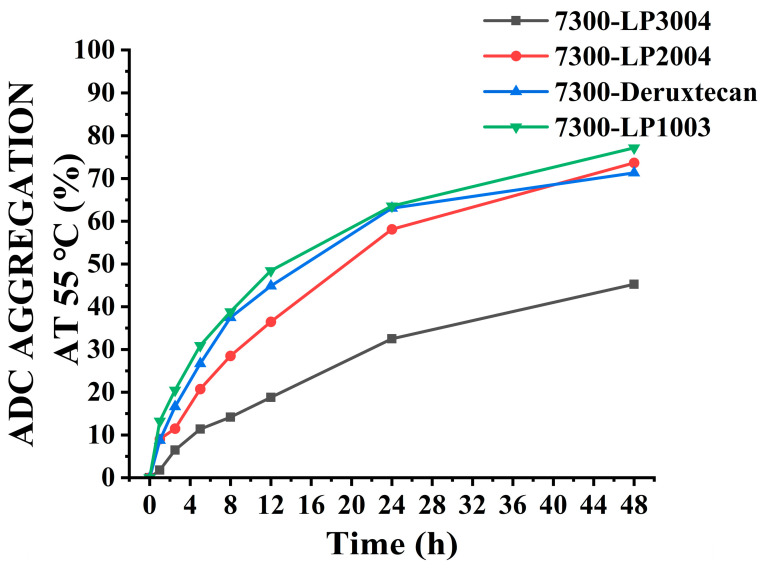
Thermostability of DAR8 ADCs. ADC aggregation was analyzed by SEC after heating at 55 °C.

The aggregation of ADCs after heating at 55 °C was analyzed using SEC. The increase in aggregation levels among different ADCs was compared to obtain a preliminary stability assessment for each ADC production technology. The aggregation of several ADCs increased significantly after 48 h (Figure 6, Table S1). However, the aggregation level of 7300-LP3004 was significantly lower than that of 7300-LP2004, 7300-LP1003, and 7300-Deruxtecan. These results indicated that the 7300-LP3004 candidate compounds exhibited higher stability.

### 2.6. In Vitro Cytotoxicity of ADCs in the SHP-77 Cell Line

The in vitro activity of ADCs was evaluated using a co-toxicity assay and compared to that of 7300-LP1003 and 7300-Deruxtecan (Figure S4). All ADCs exhibited specific cytotoxicity against SHP-77 cells. The IC50 values of 7300-LP3004, 7300-LP2004, 7300-LP1003, and 7300-Deruxtecan were 39.74 nM, 32.17 nM, 186.6 nM, and 124.5 nM, respectively (Table 1). Side-chain ADCs demonstrated significantly higher cytotoxicity toward SHP-77 cells compared to branch-chain ADCs.

### 2.7. Tras-LP3004 Displayed Strong Bystander Activity In Vitro

**Figure 7:**
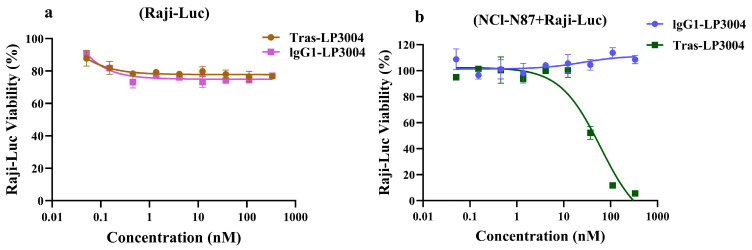
Bystander killing effect of Tras-LP3004 and lgG1-LP3004 co-culture in vitro. (**a**) Tras-LP3004 and lgG1-LP3004 were cultured separately in Raji-Luc cells. (**b**) Tras-LP3004 and lgG1-LP3004 were co-cultured in Raji-Luc and NCl-N87, respectively.

Bystander killing experiments were performed in vitro using Tras-LP3004 and lgG1-LP3004 (Tras-LP3004 refers to the ADC formed through the conjugation of trastuzumab and LP3004, while lgG1, an immunoglobulin, is not HER2-targeting). Raji-Luc cells were cultured alone or co-cultured with NCl-N87 cells (1:1 ratio of NCl-N87 to Raji-Luc). When only Raji-Luc cells were present, the two ADCs had no significant effect on the HER2-positive Raji-Luc cells (Figure 7a). However, it is noteworthy that Tras-LP3004 effectively inhibited Raji-Luc when they were cultured with NCl-N87 cells (Figure 7b). These findings suggest that Tras-LP3004 can indirectly kill Raji-Luc cells via the bystander effect of HER2-positive NCl-N87 cells.

### 2.8. Validation of ADCs In Vivo

**Figure 8:**
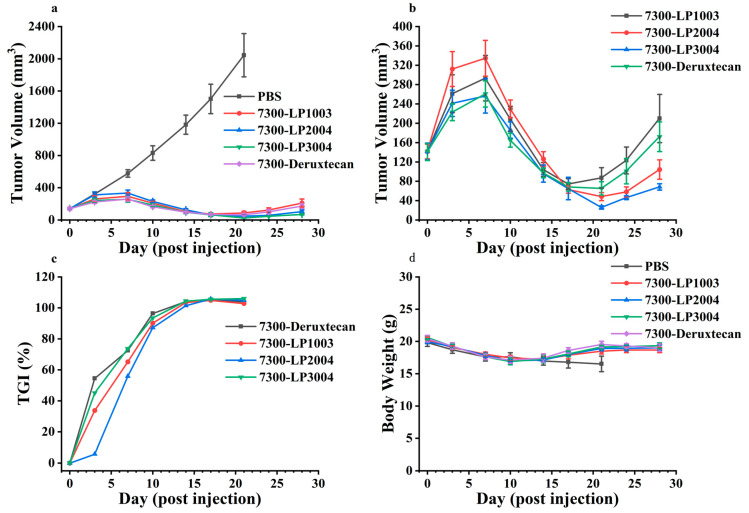
(**a**) Tumor volume changes in lung cancer tumor mice after different ADC injections. (**b**) Comparison of tumor volume between the 7300-LP3004 group, 7300-LP2004 group, 7300-LP1003 group, and 7300-Deruxtecan group. (**c**) TGI of different treatment groups. (**d**) Body weights of the mice during the treatment period.

We utilized a NOD scid gamma mouse model of the human small-cell lung cancer cell line SHP-77 to evaluate the activity of different ADCs. The animals were divided into six groups and administered an ADC dose of 5 mg/kg. After 21 days of 7300-LP3004 injected, no abnormalities were observed during necropsy. When the average tumor volume in the control group exceeded 2000 mm^3^ after 24 days of ADC injection, the mice were euthanized. As shown in Figure 8a, 7300-LP3004, 7300-LP2004, 7300-LP1003, and 7300-Deruxtecan all significantly inhibited tumor growth and showed clear therapeutic effects. Twenty-one days after ADC injection, the average tumor volume in the phosphate-buffered saline group was 2044.31 mm^3^, and the tumor volumes of the 7300-LP3004, 7300-LP2004, 7300-LP1003, and 7300-Deruxtecan groups were 25.90 mm^3^, 48.37 mm^3^, 87.19 mm^3^, and 68.37 mm^3^, respectively. Notably, 7300-LP3004 had better tumor suppression and therapeutic effects than 7300-LP2004, 7300-LP1003, and 7300-Deruxtecan (Figure 8b). As shown in Figure 8c and Table S2, the tumor growth inhibition (TGI) of 7300-Deruxtecan and 7300-LP1003 were 103.95% and 102.84%, respectively. However, 7300-LP2004 and 7300-LP3004 showed better results at a TGI of 104.90% and 106.09%, respectively. The TGI of the SHP-77 model changed from large to small as: 7300-LP3004 > 7300-LP2004 > 7300-Deruxtecan > 7300-LP1003. These results showed that 7300-LP3004 of the candidate compound can significantly improve the inhibition and therapeutic effect of ADC on tumors compared to 7300-LP2004, probably owing to its stronger hydrophilicity. Twenty-eight days after the injection of 5 mg/kg ADC, there was no significant change in the body weight of mice (Figure 8d), and no symptoms such as pain or inability to drink and eat were observed, indicating that all four ADCs were well-tolerated. The independence test of the samples was presented in Tables S3 and S4.

## 3. Discussion

In the study of ADCs, the payload and linkers play an important role in the efficacy of conjugates. In previous studies, most ADCs were associated with microtubule inhibitors, DNA alkylating agents, and topoisomerase I (TOP1) inhibitors [25,26,27]. 7300-Deruxtecan is a payload of the camptothecin compound Dxd, which has been approved for the treatment of tumors [28,29]. Therefore, camptothecin TOP1 inhibitors as ADC payloads have been the focus of ADC research. However, when the DAR of the ADCs was higher than four, the increased hydrophobicity of camptothecin caused the ADCs to aggregate during the coupling process, which was not conducive to their use [30]. Therefore, it is essential to design linkers with strong hydrophilicity, high stability, and easy-to-release payloads for tumor cells. ADC design should enhance the hydrophilicity of the linker and select a payload with high cellular activity so that ADCs have higher drug loads, thereby enhancing the concentration of toxins in tumor cells and further improving their inhibitory effects on tumors. In this study, we investigated a class of ADCs with potential clinical applications. PSar or PEG was selected to connect to the linker because hydrophilic ADCs help mitigate the aggregation and rapid clearance problems caused by a high DAR, resulting in a uniform and hydrophilic ADC. Simultaneously, the camptothecin derivative 095, which exhibits stronger activity than Dxd, was used to replace SN38 as the payload.

7300-LP3004 contained a PSar-modified Lys-Val-Ala-PAB linker, 7300-LP2004 contained a PEG-modified Lys-Val-Ala-PAB linker, and 7300-LP1003 contained a PEG-modified linker. Several ADCs were studied using the same DAR8 to determine their physicochemical properties both in vitro and in vivo. Many studies have shown that an increase in the DAR value increases the concentration of endotoxins in tumor cells, leading to side effects such as increased hydrophobicity of ADC, easy generation of aggregates, and accelerated plasma clearance [31]. The addition of PEG or PSar effectively reduced the hydrophobicity of ADCs. In the present study, the addition of PEG or PSar produced a uniform DAR8 ADC. HIC showed that 7300-LP3004 was more hydrophilic than 7300-LP2004. The cathepsin B release experiment showed that LP3004 released the payload more readily than LP2004. SEC showed that 7300-LP3004 aggregated less than 7300-LP2004, 7300-LP1003, and 7300-Deruxtecan.

Here, we evaluated the bystander-killing abilities of Tras-LP3004 and lgG1-LP3004 in Raji-Luc cells cultured alone and in a co-culture experiment with NCl-N87 cells, which demonstrated the effective bystander-killing ability of PSar-based ADCs. In our in vivo experiments, no significant weight changes were observed in the mice injected with 5 mg/kg of ADC. Compared with the control group, 7300-LP3004, 7300-LP2004, 7300-LP1003, and 7300-Deruxtecan showed clear antitumor effects; however, the inhibitory and therapeutic effects of 7300-LP3004 were superior to those of 7300-LP2004, 7300-LP1003, and 7300-Deruxtecan. This result can be explained by the fact that PSar is highly hydrophilic and exhibits excellent biocompatibility, which contributes to the prolonged circulation time of 7300-LP3004 in the bloodstream [21]. This stabilization further enhanced the bioavailability and tumor-targeting efficiency of the ADC, contributing to its superior in vivo performance; thus, its antitumor effect is the most pronounced. The inhibitory effect of 7300-LP2004 on the tumor was superior to that of 7300-LP1003. Using the same hydrophilic linker, PEG-linked Lys-Val-Ala-PAB demonstrated a more favorable effect compared to PEG-linked Val-Ala-PAB. This result can be explained by the 55 °C stability experiment, in which 7300-LP1003 exhibited the highest degree of aggregation, which may be more pronounced in the human body than that of 7300-LP2004; therefore, the therapeutic effect was poor.

In summary, we report that the DAR8 ADC, formed by the combination of a linker and payload, can maintain the ADC at a high DAR without compromising the drug efficacy. 7300-LP3004 showed significant antitumor properties in lung cancer models, superior to those of 7300-Deruxtecan and PEG-modified ADCs. Finally, our results support the conclusion that using a PSar linker in the Lys-Val-Ala-PAB connector technology is of great significance for the development of ADCs at a later stage. The connector improved the hydrophilicity of ADCs and reduced their aggregation, aiding in the development of different ADCs.

## 4. Materials and Methods

### 4.1. Preparation of ADCs

In a solution of lgG1 monoclonal antibodies (7300) targeting B7H3 (5 mg/mL in PBS (pH 7.4), 10 molar equivalents of tris(2-carboxyethyl)phosphine (TCEP) were added, and the mixture was incubated at 37 °C for 1 h. After monitoring the complete reduction of the antibody by RPLC, 12 molar equivalents of the LP were added to the reduced antibody along with DMSO to maintain a 15% (*v*/*v*) concentration for conjugation, followed by incubation at 37 °C for 2 h. Once RPLC monitoring indicated that the reduced antibody was fully consumed, L-cysteine was added to quench any unreacted LP. The conjugates were then sterile-filtered using a 0.2 μm PES filter, and the buffer was exchanged with PBS (pH 7.4) via a high-speed refrigerated centrifuge (Merrick instrument, Cincinnati, OH, USA). Protein concentration was measured spectrophotometrically at 280 nm using a microspectrophotometer (Zuofei, Guangzhou, China), and the final ADC was stored at −80 °C.

### 4.2. Characterization of Linker–Payload

2.5 mg of the linker–payload was weighed and dissolved in 0.5 mL of DMSO, followed by a slow dropwise addition of 4.5 mL of distilled water. NanoZS Zetasizer instrument (Malvern Panalytical, Malvern, UK) was used to characterize the particles in terms of size, PDI, and zeta potential. The instrument was used at 25 °C to determine the zeta potential, size, and PDI of the linker–payload.

### 4.3. Cathepsin B-Mediated Cleavage Assay of Linker–Payload

Cathepsin B (100 μg, ≥5 units/mg protein) derived from human placenta was solubilized in 200 μL of buffer (PBS, 1 mM EDTA, pH 7.2). Forty microliters (40 μL) of cathepsin B solution was activated by adding 80 μL of buffer B (PBS, 32 mM DTT, 17 mM EDTA, pH 7.2). Twenty microliters (20 μL) of the test compound (5 mM) was added with 20 μL of N-acetylcysteine (25 mM), DMF (70 μL) and 890 μL of buffer (PBS pH 7.2) and incubated at 37 °C for 30 min. The incubated sample (60 μL) was added dropwise to the activated cathepsin B solution and incubated at 37 °C. Twenty microliters (20 μL) of the final solution was collected at different time points, and protein precipitation was performed by adding 20 μL of cold ACN before being centrifuged at 4 °C for 20 min at 15,000 rpm. The supernatant was collected for analysis. All samples were analyzed using an Agilent 1260 Infinity II HPLC system (Agilent Technologies, Waldbronn, Germany) equipped with a Shim-pack GIST C18 column (2.1 mm × 100 mm, 2.0 μm; Shimadu, Tokyo, Japan). The system operated at a temperature of 37 °C for 13 min and a flow rate of 0.5 mL/min. Mobile phase A consisted of water containing 0.1% H_3_PO_4_, while mobile phase B comprised ACN. The gradient program employed the following conditions: B%: 28% to 49% (0–7 min), 49% to 90% (7–10 min), 90% to 28% (10–10.01 min), and 28% (10.01–13 min)

### 4.4. Characterization of Antibody–Drug Conjugates

Reversed-phase liquid chromatography (RPLC) was performed on an Agilent 1260 Infinity II HPLC system (Agilent Technologies, Germany). The column used was a BioResolve RP mAb Polyphenyl column, 450 Å (2.1 mm × 300 mm, 2.7 μm; Waters, Drinagh, Ireland). The system operated at a temperature of 80 °C for 28 min with a flow rate of 0.3 mL/min. Mobile phase A consisted of water containing 0.1% TFA, while mobile phase B comprised ACN containing 0.1% TFA. The gradient program employed the following conditions: B%: 25% to 35% (0–4 min), 35% to 42% (4–18 min), 42% to 80% (18–19 min), 80% (19–21 min), and 80% to 25% (21–21.01 min).

HIC was performed using a TSKgel Butyl-NPR column (4.6 mm I.D. × 10 cm, 2.5 μm, Tosoh Corporation, Tokyo, Japan). The system operated at a temperature of 30 °C for 30 min and a flow rate of 1 mL/min. Mobile phase A consisted of 25 mM sodium phosphate and 1.5 M ammonium sulfate (pH 7.0), while mobile phase B consisted of 25 mM sodium phosphate and 10% isopropanol (pH 7.0). The gradient program employed the following conditions: B%: 10% to 10% (0–2 min), 10% to 100% (10 min), 100% to 100% (12–17 min), and 10% (17.01–25 min). UV detection was performed at 280 nm.

SEC was performed on a LC-20 AT system (Shimadzu, Tokyo, Japan). The column used was a TSKgel G3000SWXL (7.8 mm × 300 mm, 5 um; Tosoh Corporation, Tokyo, Japan). The system was run at 30 °C for 30 min in a 0.6 mL/min isogradient, and the mobile phase consisted of 150 mM NaCl, 200 mM sodium phosphate, and 15% (*v*/*v*) ACN. UV detection was performed at 280 nm.

### 4.5. Stability Study

Each ADC (0.5 mg/mL) was incubated at 55 °C in PBS (pH 7.2). At each time point (0 h, 1 h, 2.5 h, 5 h, 8 h, 12 h, 24 h, and 48 h), an equal volume (50 μL) was collected, and the percentage of ADC aggregation was analyzed by SEC.

### 4.6. Cell Culture

The human small cell lung cancer cell line SHP-77 was cultured in RPMI-1640 medium supplemented with 10% fetal bovine serum (FBS) at 37 °C in a 5% CO_2_ atmosphere. SHP-77 cells in the exponential growth phase were collected and resuspended in PBS at an appropriate concentration for subcutaneous tumor inoculation in mice. The SHP-77 cells were authenticated by short tandem repeat (STR) analysis.

### 4.7. In Vitro Cytotoxicity Assays

SHP-77 cells were grown in RPMI-1640 medium supplemented with 10% FBS and 1 μg/mL puromycin at 37 °C in a 5% CO_2_ incubator. SHP-77 cells were dissociated using TrypLE^TM^ Express Enzyme (Thermo Fisher Scientific, Waltham, MA, USA) and, after counting, the cells were resuspended in RPMI-1640 medium supplemented with 10% FBS at a density of 3 × 10^4^ cells/mL. The cells were plated in 96-well plates and incubated at 37 °C for 24 h. ADCs and payloads were diluted separately and added to the 96-well cell culture plates containing cells, followed by incubation in a 5% CO_2_ incubator for 5 days. A multifunctional microplate reader was used, and the absorbance was measured at 450 nm with a reference wavelength of 630 nm. The IC50 values were calculated using GraphPad Prism 10.1.2 software.

### 4.8. In Vitro Bystander Killing Assay

In the experiment, the cell line Raji-Luc was cultured alone or mixed at the ratio of 1:1 (1 NCl-N87: 1 Raji-Luc), with a total of 4000 cells per well in a 96-well plate, and incubated overnight at 37 °C in the presence of 5% CO_2_. ADC samples were diluted and incubated at concentrations of 333.33, 111.11, 37.04, 12.35, 4.12, 1.37, 0.46, 0.15, and 0.05 nM at 37 °C in a 5% CO_2_ incubator for 5 days, respectively. After incubation, 100 μL Bright-Glo^TM^ (Promega Corporation, Madison, WI, USA) reagent was added to each well and incubated at room temperature for 5 min. A Nivo instrument was used for analysis, and data were analyzed using GraphPad Prism software.

### 4.9. In Vivo Efficacy Experiments

Mice aged 6 to 8 weeks were housed in autoclaved cages and maintained under sterile conditions. A total of 3 × 10^6^ SHP-77 cells were resuspended in a 1:1 mixture of PBS and Matrigel (0.15 mL per mouse) and injected subcutaneously into the right flank of the mice. When the tumor volume reached approximately 150 mm^3^, the mice were randomly divided into treatment and control groups based on tumor size and volume. ADCs were administered intravenously at a dose of 5 mg/kg once (*n* = 6 mice per group). Tumor volumes were measured twice weekly (every 3–4 days) using a vernier caliper (length × width), and the following formula was used: V = 0.5 × (length × width^2^). The tumor growth inhibition (TGI, %) was calculated as follows:

TGI = (1 − ϭT/ϭC) × 100%

ϭT and ϭC: These represent the change in tumor volume (final–initial) for the treated and control groups, respectively [32].

TGI = 0%: No inhibition. TGI = 100%: Complete growth arrest. TGI > 100%: Tumor regression. TGI < 0%: Adverse effect.

The mice were euthanized when they reached the endpoint criteria (tumor volume exceeding 2000 mm^3^ or >30% reduction of body.

### 4.10. Statistics

In vivo experiment data were represented as means +/− SEM. An independent sample *t*-test was used to compare the relative tumor volume and tumor weight between the treatment and control groups. Statistical significance was evaluated using SPSS 28.0 software. *p*-values were represented as * (*p* < 0.05), ** (*p* < 0.01), and *** (*p* < 0.001), with “ns” standing for non-significant.

## Figures and Tables

**Table 1:** IC50 values of ADCs.

ADC	IC50
7300-LP2004	32.17 nM
7300-LP3004	39.74 nM
7300-LP1003	186.6 nM
7300-Deruxtecan	124.5 nM

## Data Availability

Data are available upon request to the Corresponding Authors.

## Supplementary Materials

The following supporting information can be downloaded at: https://www.mdpi.com/article/10.3390/molecules30071398/s1. Abbreviation, the synthesis of compounds, and copies of selected ^1^H NMR, ^13^C NMR, and Mass Spectra are provided in the Supplementary Materials. Supplementary Figure S1. Cell cytotoxicity profiles. Supplementary Figure S2. (a) Payload was used as a control. (b) LP3004 without cathepsin B. (c) LP3004 with cathepsin B for 24 h. (d) LP2004 without cathepsin B. (e) LP2004 with cathepsin B for 24 h. Supplementary Figure S3. (a) MS analysis of 7300-LP3004. (b) MS analysis of 7300-LP2004. (c) MS analysis of 7300-LP1003. (d) MS analysis of 7300-Deruxtecan. Supplementary Table S1: Stability testing of ADCs in PBS at 55 °C. Supplementary Table S2. Subcutaneous tumor model grouping, administration regimen, and TGI after drug treatment of SHP-77 mice. Supplementary Figure S4. Cell cytotoxicity profiles. Supplementary Table S3. Independent sample testing of mouse tumors. * (*p* < 0.05), ** (*p* < 0.01), “ns” non-significant. Supplementary Table S4. Independent sample testing of mouse body weight. * (*p* < 0.05), ** (*p* < 0.01), and *** (*p* < 0.001), “ns” non-significant.
