# Supplementary material for: Design, Synthesis, and Evaluation of Camptothecin-Based Antibody–Drug Conjugates with High Hydrophilicity and Structural Stability
Source: Molecules. 2025 Mar 21;30(7):1398. doi: 10.3390/molecules30071398 (PMC11990160; doi:10.3390/molecules30071398)
Supplement: Supplementary file 1 [file molecules-30-01398-s001.zip › molecules-3542620-supplementary.pdf]

Supporting Information

Design, Synthesis, and Evaluation of Camptothecin-Based Antibody–Drug Conjugates with High Hydrophilicity and Structural Stability

Tingyu Xiong [1,2,†], Jiyu Jin [2,3,†], Dongliang Liu[1,*], Chen Jin[2,*]

[1] College of Chemistry and Chemical Engineering, Donghua University, 2999 North Renmin Road, Shanghai 201620, China
[2] Shanghai Tekanbio Pharm-Tech Co., Ltd., Room 4001, Floor 4, Unit 3, Building 8, No. 160, Basheng Road, China (Shanghai) Pilot Free Trade Zone, Shanghai 200120, China
[3] Shanghai Engineering Research Center of Molecular Therapeutics and New Drug Development, School of Chemistry and Molecular Engineering, East China Normal University, 3663 North Zhongshan Road, Shanghai 200062, China
* Correspondence: dlliu@dhu.edu.cn (D.L.); jinc@tekanbio.com (C.J.); Tel.: +86-133-8160-0252 (D.L.); +86-180-2105-9803 (C.J.)
† These authors contributed equally to this work.

Abbreviation

ADC, Antibody–drug conjugates; PEG, Polyethylene glycol; PSar, Polysarcosine; MeOH, Methanol; DCM, Dichloromethane; TLC, Thin Layer Chromatography; RT, Room Temperature; THF, Tetrahydrofuran; EEDQ, 2-Ethoxy-1-ethoxycarbonyl-1,2-dihydroquinoline; MTBE, Methyl Tert-Butyl Ether; EA, Ethyl acetate; AcOH, Acetic Acid; PPTS, Pyridinium p-toluenesulfonate; EtOH, Ethanol; DMF, Dimethylformamide; HATU, Hexafluorophosphate Azabenztriazole Tetramethyl Uronium; DEA, Diethylamine; DIPEA, N,N-Diisopropylethylamine; PBS, Phosphate-Buffered Saline; ACN, Acetonitrile; DMSO, Dimethylsulfoxide; FA, Formic Acid; TEA, Triethylamine; PAB, Para-aminobenzyl; PDI, Polymer dispersity index; LP, Linker Payload; TGA, Thermogravimetric analysis; HPLC, High-Performance Liquid Chromatography; LC-MS, Liquid Chromatography–Mass Spectrometry; DAR, Drug to Antibody Ratio; HIC, Hydrophobic interaction chromatography; SEC, Size exclusion chromatography; pH, Potential of Hydrogen; EDTA, Ethylenediaminetetraacetic acid; TCEP, Tris(2-carboxyethyl)phosphine; RPLC, Reverse-Phase Liquid Chromatography; DTT, Dithiothreitol; TGI, Tumor Growth Inhibition; SEM, Standard Error of the Mean.

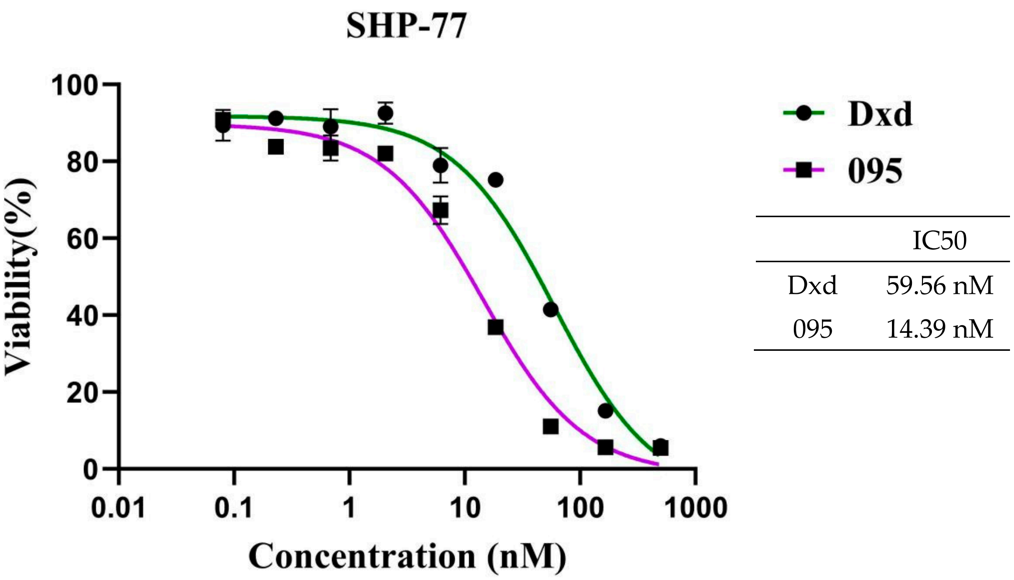

	IC50
Dxd	59.56 nM
095	14.39 nM

Figure S1. Cell cytotoxicity profiles.

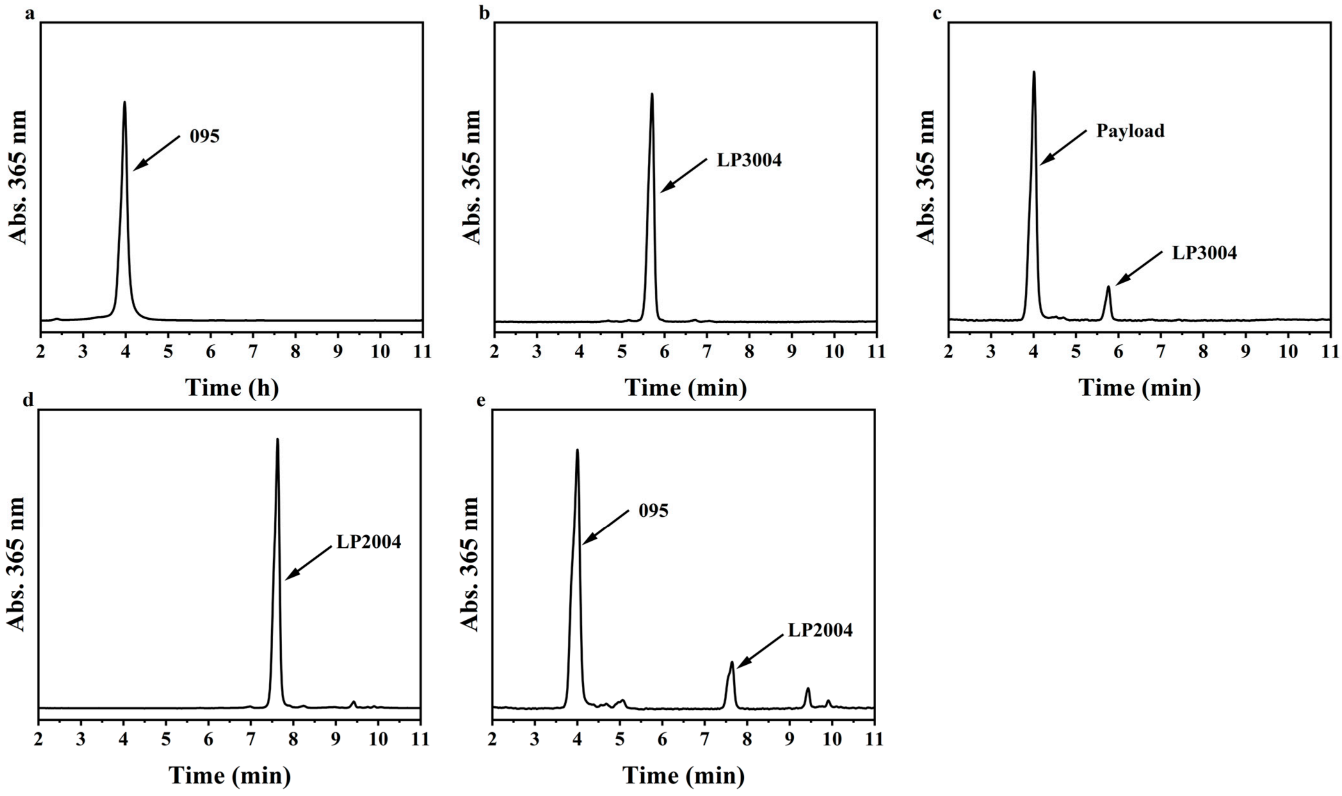

Figure S2. (a) Payload was used as a control. (b) LP3004 without cathepsin B. (c) LP3004 with cathepsin B for 24 h. (d) LP2004 without cathepsin B. (e) LP2004 with cathepsin B for 24 h.

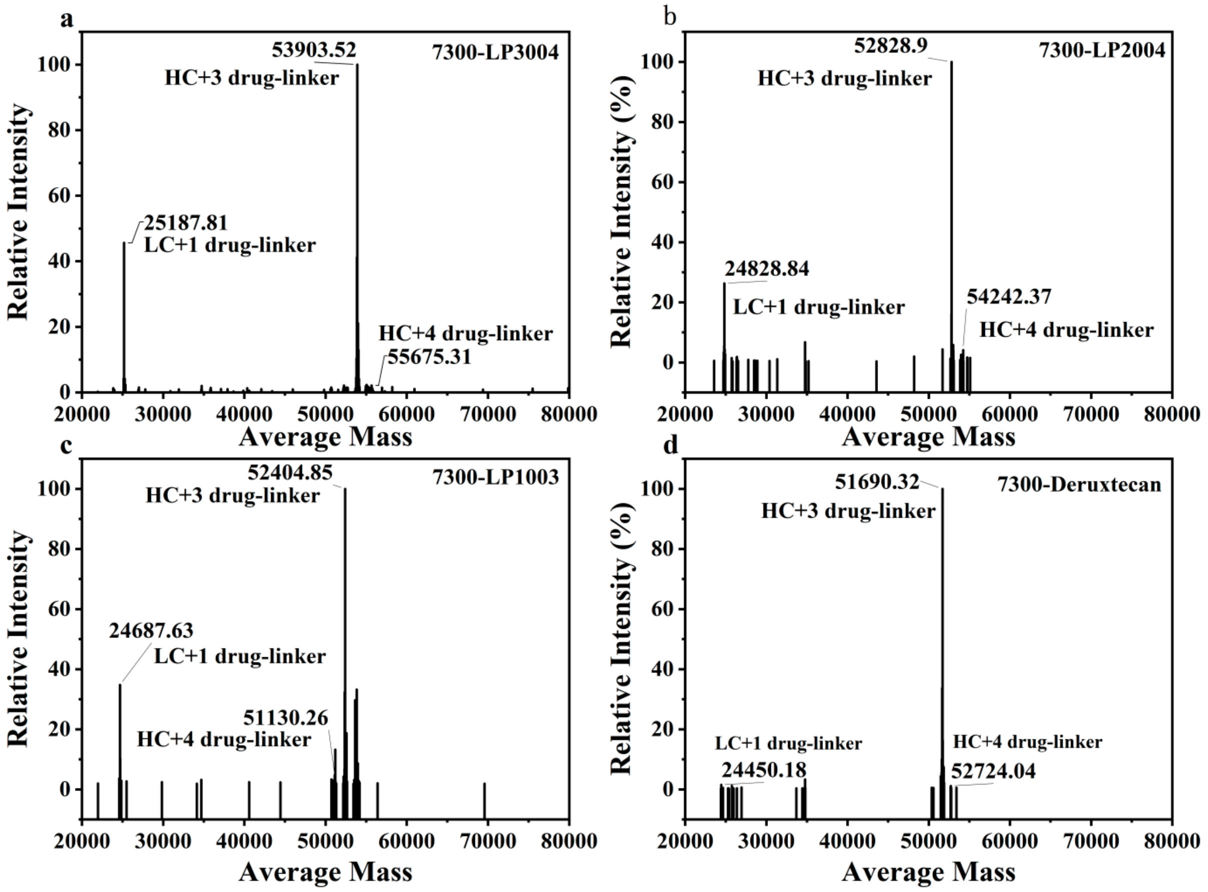

Figure S3. (a) MS analysis of 7300-LP3004. (b) MS analysis of 7300-LP2004. (c) MS analysis of 7300-LP1003. (d) MS analysis of 7300-Deruxtecan.

Table S1: Stability testing of ADCs in PBS at 55℃.

Time	7300-Deruxtecan	7300-LP1003	7300-LP2004	7300-LP3004
h	%	%	%	%
0	0	0	0	0
1	8.745	13.226	9.033	1.773
2.5	16.611	20.468	11.476	6.467
5	26.693	30.909	20.746	11.38
8	37.473	38.805	28.499	14.171
12	44.865	48.446	36.461	18.781
24	63.05	63.558	58.112	32.484
48	71.325	77.139	73.652	45.244

Table S2. Subcutaneous tumor model grouping, administration regimen, and TGI after drug treatment of SHP-77 mice.

Group	Route of administration	Administration dose (mg/kg)	Dose frequency	TGI (%)
PBS	Intraperitoneal injection	/	1	NA
7300-Deruxtecan	Intraperitoneal injection	5	1	103.95

7300-LP1003	Intraperitoneal injection	5	1	102.84
7300-LP2004	Intraperitoneal injection	5	1	104.90
7300-LP3004	Intraperitoneal injection	5	1	106.09

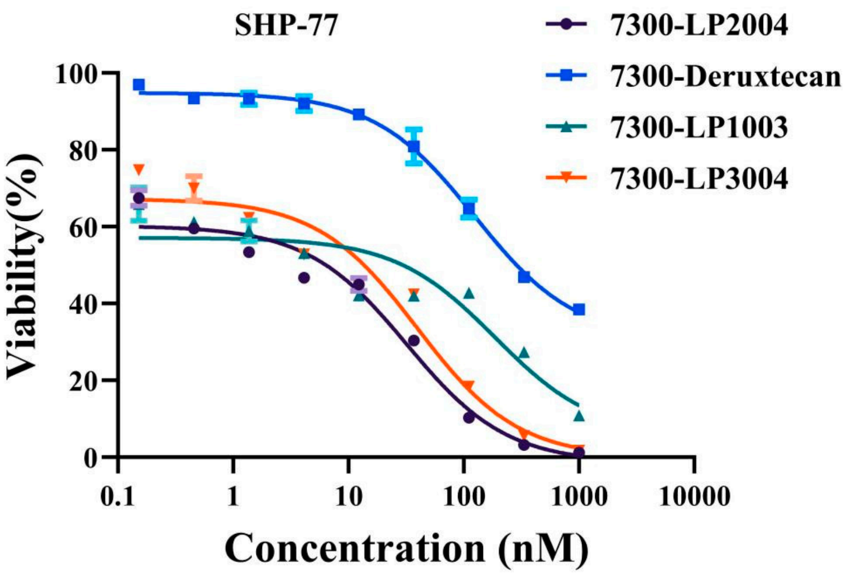

Figure S4. Cell cytotoxicity profiles.

Table S3. Independent sample testing of mouse tumors. * ($p < 0.05$), ** ($p < 0.01$, "ns" non-significant.

/	Injection of 7300-Deruxtecan		Injection of 7300-LP1003		Injection of 7300-LP2004		Injection of 7300-LP3004	
Day	p-value	sign	p-value	sign	p-value	sign	p-value	sign
0	0.6800	ns	0.6750	ns	0.8700	ns	0.6850	ns
3	0.3380	ns	0.4800	ns	0.2900	ns	0.9580	ns
7	0.3810	ns	0.9340	ns	0.6250	ns	0.5890	ns
10	0.0060	**	0.0180	*	0.0080	**	0.0080	**
14	0.0030	**	0.0040	**	0.0040	**	0.0050	**
17	0.0210	*	0.0220	*	0.0200	*	0.0310	*
21	0.0400	*	0.0480	*	0.0360	*	0.0330	*

Table S4. Independent sample testing of mouse body weight. * ($p < 0.05$), ** ($p < 0.01$), and *** ($p < 0.001$), "ns" non-significant.

/	Injection of 7300-Deruxtecan		Injection of 7300-LP1003		Injection of 7300-LP2004		Injection of 7300-LP3004	
Day	p-value	sign	p-value	sign	p-value	sign	p-value	sign
0	0.5420	ns	0.1070	ns	0.0590	ns	0.0240	*
3	0.4630	ns	0.0250	*	0.0880	ns	0.1170	ns
7	0.0320	*	0.0040	**	0.0010	**	0.0490	*
10	0.4880	ns	0.0540	ns	0.0030	**	0.4800	ns
14	0.8300	ns	0.4980	ns	0.0180	*	0.7190	ns
17	0.0150	*	0.1360	ns	0.0020	**	0.0240	*
21	0.0040	**	0.0020	**	0.0010	**	0.0000	***

Synthesis of Linker–Payload

Scheme 1. Synthesis of LP1003

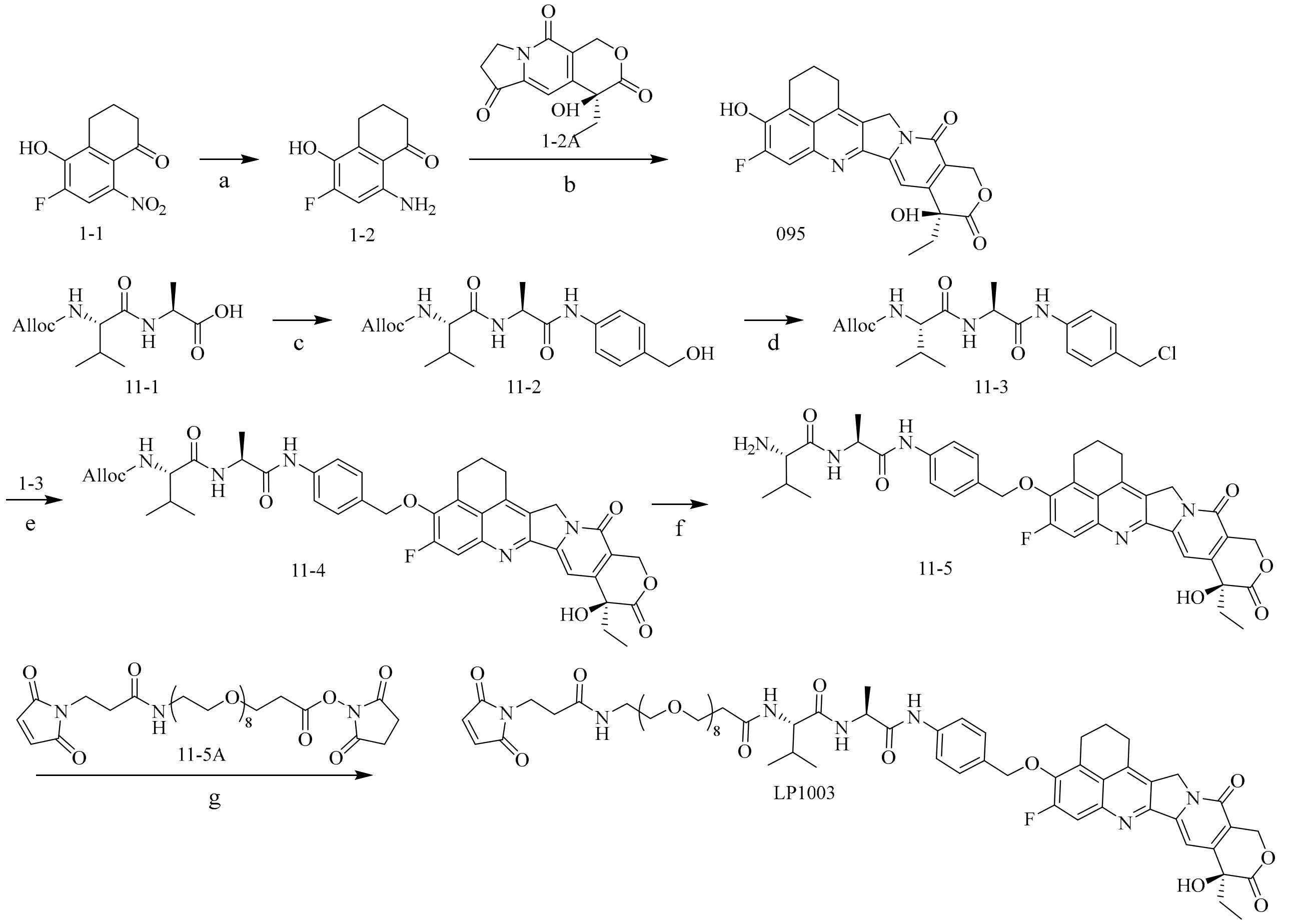

Synthesis of intermediate 1-2

Compound 1-1 (15.00 g, 66.61 mmol) was added to MeOH (750 mL) and stirred at RT. Raney nickel (15 g, 255.57 mmol) was added to the reaction vessel. The reaction mixture was stirred under hydrogen gas for 2 h until TLC (DCM/MeOH 20/1) showed that compound 1-1 had been consumed. The reaction mixture was filtered, and the filtrate was concentrated under reduced pressure to obtain a brown solid (12.90 g, 99.23%), which was used directly in the next step without further purification. LC-MS (ESI$^+$) m/z 196.3 (M+H)$^+$.

Synthesis of intermediate 095

Compound 1-2 (8.67 g, 44.42 mmol) were added to toluene (90 mL) and AcOH (90 mL) and stirred at RT. Compounds 1-2A (15.20 g, 57.74 mmol) and PPTS (2.58 g, 10.25 mmol) were added to the reaction vessel. The mixture was then heated to 110°C and stirred under N_2 atmosphere for 24 h until TLC (DCM/MeOH 10/1) showed that compound 1-2 had been consumed. The reaction mixture was concentrated under reduced pressure, and then AcOH (45 mL) was added to be redissolved. This was added dropwise to water (450 mL), and the brown solid was gradually precipitated and filtered. The filter cake was washed with water (20 mL) and dried to obtain the crude product. The crude product was triturated using DCM/MTBE/EtOH (90 mL/90 mL/1.5 mL) to obtain a brown solid (12.00 g, 83.16%). LC-MS (ESI$^+$) m/z 423.2 (M+H)$^+$. ^{1}H NMR (400 MHz, DMSO-*d*$_6$) δ 10.23 (s, 1H), 7.73 (d, *J* = 11.8 Hz, 1H), 7.24 (s, 1H), 6.50 (s, 1H), 5.42 (s, 2H), 5.17 (s, 2H), 3.08 (t, *J* = 6.2 Hz, 2H), 3.01 (t, *J* = 6.2 Hz, 2H), 2.01 (p, *J* = 6.5 Hz, 2H), 1.87 (td, *J* = 14.4, 13.1, 6.1 Hz, 2H), 0.88 (t, *J* = 7.3 Hz, 3H).

Synthesis of intermediate 11-2

Compound 11-1 (10.00 g, 23.84 mmol) was added to THF (200 mL) and stirred at RT. (4-Aminophenyl) methanol (5.20 g, 42.23 mmol) and EEDQ (10.44 g, 42.23 mmol) were added to the reaction vessel. The mixture was stirred under N_2 atmosphere for 24 h until TLC (DCM/MeOH 10/1) showed that compound 11-1 had been consumed. The reaction mixture was concentrated under reduced pressure, and then THF (14 mL) was added to be redissolved. This was added dropwise to MTBE (140 mL), and the yellow solid was gradually precipitated and filtered. The filter cake was washed with MTBE (20 mL) and dried to obtain a yellow solid (8.80 g, 63.49%). LC-MS (ESI^+) m/z 378.2 $(M+H)^+$. ^1H NMR (400 MHz, DMSO-d_6) δ 9.90 (s, 1H), 8.14 (d, J = 7.0 Hz, 1H), 7.52 (d, J = 8.1 Hz, 2H), 7.24 (t, J = 9.9 Hz, 3H), 5.90 (ddt, J = 16.5, 10.8, 5.5 Hz, 1H), 5.29 (d, J = 17.2 Hz, 1H), 5.16 (d, J = 10.6 Hz, 1H), 5.09 (t, J = 5.6 Hz, 1H), 4.44 (dd, J = 21.5, 5.4 Hz, 5H), 3.88 (t, J = 7.9 Hz, 1H), 1.96 (d, J = 6.8 Hz, 1H), 1.29 (d, J = 7.0 Hz, 3H), 0.85 (dd, J = 18.9, 6.8 Hz, 6H). ^{13}C NMR (101 MHz, DMSO-d_6) δ 171.48, 171.38, 156.48, 138.02, 137.89, 134.09, 127.39, 119.33, 117.42, 64.91, 63.05, 60.41, 49.45, 30.81, 19.64, 18.57.

Synthesis of intermediate 11-3

Compound 11-2 (4.50 g, 11.92 mmol) was added to anhydrous THF (90 mL) and stirred at 0°C. Sulfur oxychloride (1.73 mL, 23.84 mmol) was slowly added to the reaction vessel. The mixture was then heated to 20°C and stirred under N_2 atmosphere for 2 h until TLC (DCM/MeOH 10/1) showed that compound 11-2 had been consumed. The reaction mixture was concentrated under reduced pressure to obtain a light-yellow solid (4.44 g, 94.07%), which was used directly in the next step without further purification. LC-MS (ESI^+) m/z 396.2 $(M+H)^+$.

Synthesis of intermediate 11-4

Compound 095 (4.00 g, 9.47 mmol) were added to DMF (80 mL) and stirred at RT. Compounds 11-3 (8.81 g, 22.25 mmol) and pempidine (8.05 mL, 44.51 mmol) were added to the reaction vessel. The mixture was then heated to 50°C and stirred under N_2 atmosphere for 24 h until TLC (DCM/MeOH 10/1) showed that compound 095 had been consumed. The mixture was cooled down to 20 °C, added dropwise to water (800 mL), and the brown solid was gradually precipitated and filtered. The filter cake was washed with water (100 mL) and dried under reduced pressure. The crude product was purified by silica gel chromatography (DCM/MeOH gradient from 100:1 to 20:1). The solvent was concentrated under reduced pressure to obtain a white solid (2.80 g, 38.84%). LC-MS (ESI^+) m/z 782.3 $(M+H)^+$. ^1H NMR (400 MHz, DMSO-d_6) δ 10.03 (s, 1H), 8.18 (d, J = 6.9 Hz, 1H), 7.80 (d, J = 12.3 Hz, 1H), 7.60 (d, J = 8.1 Hz, 2H), 7.38 (d, J = 8.1 Hz, 2H), 7.32 - 7.24 (m, 2H), 6.52 (d, J = 1.9 Hz, 1H), 5.91 (ddt, J = 17.0, 11.7, 5.8 Hz, 1H), 5.42 (s, 2H), 5.30 (d, J = 17.2 Hz, 1H), 5.17 (d, J = 12.3 Hz, 3H), 5.08 (s, 2H), 4.45 (dd, J = 24.4, 6.0 Hz, 3H), 3.89 (t, J = 8.0 Hz, 1H), 3.01 (dt, J = 39.7, 6.1 Hz, 4H), 1.91 (ddq, J = 27.2, 14.0, 6.7 Hz, 5H), 1.31 (d, J = 7.0 Hz, 3H), 0.87 (dq, J = 16.0, 6.7, 5.0 Hz, 9H). ^{13}C NMR (101 MHz, DMSO-d_6) δ 172.90, 171.62, 171.52, 158.66, 157.13, 156.48, 156.15, 151.55, 150.38, 146.12, 145.81 (d, J = 12.6 Hz), 142.23 (d, J = 14.4 Hz), 141.47, 139.57, 134.08, 131.71, 129.88, 129.79, 126.16, 122.72, 119.34, 119.30, 117.40, 112.02, 111.83, 97.02, 75.43, 72.83, 65.72, 64.91, 60.38, 49.52, 30.82, 26.51, 23.90, 20.96, 19.63, 18.55, 8.22.

Synthesis of intermediate 11-5

Compound 11-4 (2.30 g, 9.47 mmol) was added to DMF (23 mL) and stirred at RT. $Pd(PPh_3)_4$ (33.99 mg, 29.42 μmol) and piperidine (2.32 mL, 23.53 mmol) were added to the reaction vessel. The reaction mixture was stirred under N_2 atmosphere for 2 h until TLC (DCM/MeOH 10/1) showed that compound 11-4 had been consumed. The reaction mixture was concentrated under reduced pressure. The residue was purified by silica gel chromatography (DCM/MeOH gradient from 100:1 to 30:1). The solvent was concentrated under reduced pressure to obtain a yellow solid (1.60 g, 78.05%). LC-MS (ESI^+) m/z 698.6 $(M+H)^+$. ^1H NMR (400 MHz, DMSO-d_6) δ 10.10 (s, 1H), 8.17 (d, J = 7.2 Hz, 1H), 7.80 (d, J = 12.3 Hz, 1H), 7.60 (d, J = 8.1 Hz, 2H), 7.39 (d, J = 8.1 Hz, 2H), 7.28 (s, 1H), 6.51 (s, 1H), 5.42 (s, 2H), 5.12 (d, J = 27.7 Hz, 4H), 4.45 (q, J = 7.6, 7.1 Hz, 1H), 3.19 - 2.90 (m, 5H), 1.98 - 1.72 (m, 5H), 1.30 (d, J = 7.0 Hz, 3H), 0.98 - 0.84 (m, 6H), 0.78 (d, J = 6.8 Hz, 3H). ^{13}C NMR (101 MHz, DMSO-d_6) δ 173.80, 172.91, 171.68, 158.68, 157.17, 156.18, 151.63, 150.42, 146.17, 145.84 (d, J = 7.8 Hz), 142.26 (d, J = 10.2 Hz), 141.55, 139.52, 131.79, 129.91, 129.82, 126.26, 122.79, 119.46, 119.30, 112.06, 111.87, 97.05, 75.46, 72.84, 65.73, 59.73, 49.23, 31.70, 30.81, 26.52, 23.93, 21.01, 19.78, 18.97, 17.52, 8.23.

Synthesis of intermediate LP1003

Compound 11-5 (0.30 g, 429.94 μmol) was added to ACN (3 mL) and stirred at 0°C. Compound 11-5A (444.81 mg, 644.92 μmol) and TEA (59.93 μL, 429.94 μmol) were added to the reaction vessel. The reaction mixture was stirred under N_2 atmosphere for 0.5 h until HPLC showed that compound 11-5 had been consumed. The reaction mixture was concentrated under reduced pressure to obtain a crude product. The crude product was dissolved in DMSO (5 mL) and cleaned up with a C18 Spherical 20 ~ 35 μm 100 Å cartridge. The mobile phase A was water + 0.1% FA, and the mobile phase B was ACN. The gradient range was 10 ~ 50% B. The solvent was concentrated under reduced pressure to obtain a white solid (240 mg, 43.87%). HRMS (ES$^+$) m/z 1272.5718 (M+H)$^+$. ^{1}H NMR (400 MHz, DMSO-*d*$_6$) *δ* 9.97 (s, 1H), 8.34 - 7.16 (m, 11H), 6.53 (d, *J* = 16.4, 1H), 5.59 - 5.27 (m, 2H), 5.36 - 4.80 (m, 4H), 4.51 - 4.33 (m, 1H), 4.21 (d, *J* = 7.6, 1H), 3.48 (s, 40H), 2.82 - 2.19 (m, 4H), 2.09 - 1.64 (m, 5H), 1.30 (d, *J* = 6.9, 3H), 0.85 (dd, *J* = 16.2, 6.9, 9H)..

Scheme 2. Synthesis of LP2004

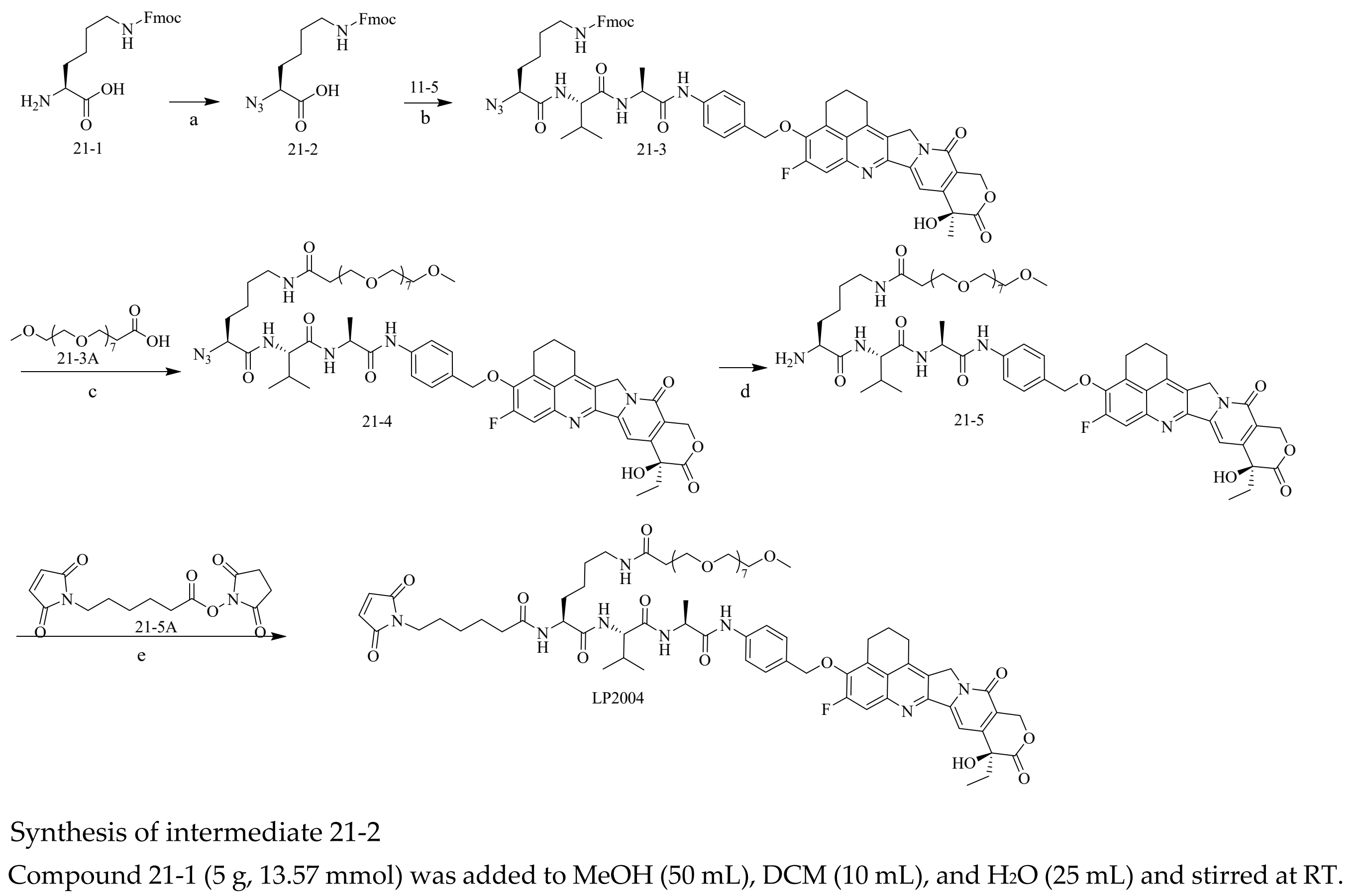

Synthesis of intermediate 21-2

Compound 21-1 (5 g, 13.57 mmol) was added to MeOH (50 mL), DCM (10 mL), and H_2O (25 mL) and stirred at RT. K_2CO_3 (3.75 g, 27.14 mmol), $CuSO_4$ (217 mg, 1.36 mmol), and 1H-imidazole-1-sulfonyl azide hydrochloride (3.00 g, 14.25 mmol) were added to the reaction vessel. The reaction mixture was stirred under N_2 atmosphere for 5 h until TLC (DCM/MeOH 10/1) showed that compound 21-1 had been consumed. After the mixture was concentrated under reduced pressure, H_2O (13 mL) was added. The mixture was adjusted to pH 3 with 4 M HCl and extracted with EA (3 × 50 mL). The organic phase was washed with saturated NaCl (2 × 100 mL), dried over Na_2SO_4, and filtered. The filtrate was concentrated under reduced pressure to obtain a light-green oil (3.00 g, 56.07%). LC-MS (ESI$^+$) m/z 395.2 (M+H)$^+$. ^{1}H NMR (400 MHz, DMSO-*d*$_6$) *δ* 13.32 (s, 1H), 7.89 (d, *J* = 7.5, 2H), 7.69 (d, *J* = 7.5, 2H), 7.37 (dt, *J* = 32.9, 7.4, 5H), 4.26 (dd, *J* = 31.3, 6.9, 3H), 4.09 (dd, *J* = 8.4, 4.9, 1H), 2.99 (q, *J* = 6.5, 2H), 1.68 (ddq, *J* = 36.7, 14.3, 7.0, 2H), 1.45 - 1.08 (m, 4H).

Synthesis of intermediate 21-3

Compound 11-5 (1.50 g, 2.15 mmol) was added to DMF (30 mL) and stirred at RT. Compounds 21-2 (1.02 g, 2.58 mmol), TEA (0.51 mL, 3.65 mmol), and HATU (1.06 g, 2.79 mmol) were added to the reaction vessel. The reaction mixture was stirred at RT for 1 h until TLC (DCM/MeOH 10/1) showed that compound 11-5 had been consumed. The

reaction mixture was added dropwise to water (300 mL), and the yellow solid was gradually precipitated and filtered. The filter cake was washed with water (20 mL) and dried to obtain a yellow solid (2.3 g, 99.57%). LC-MS (ESI^+) m/z 1074.9 $(M+H)^+$. ^1H NMR (400 MHz, DMSO-d_6) δ 10.03 (s, 1H), 8.31 (d, J = 6.7, 1H), 8.19 (d, J = 8.7, 1H), 7.84 (dd, J = 17.1, 9.9, 3H), 7.63 (dd, J = 26.6, 7.8, 4H), 7.46 - 7.16 (m, 8H), 6.52 (s, 1H), 5.42 (s, 2H), 5.13 (d, J = 35.6, 4H), 4.51 - 4.11 (m, 5H), 3.86 (t, J = 7.1, 1H), 3.02 (dd, J = 40.9, 6.3, 6H), 1.93 (dddd, J = 42.9, 28.0, 13.8, 6.9, 5H), 1.66 (q, J = 7.9, 2H), 1.48 - 1.13 (m, 7H), 0.87 (dd, J = 18.5, 7.0, 9H). ^{13}C NMR (101 MHz, DMSO-d_6) δ 172.93, 171.67, 170.98, 170.14, 158.73, 157.98, 157.18, 156.22, 151.68, 150.44, 146.22, 145.94 (d, J = 12.8 Hz), 142.98, 142.28 (d, J = 14.2 Hz), 141.55, 139.82, 137.83, 131.66, 129.97, 129.83, 129.32, 127.69, 126.32, 122.86, 121.76, 120.41, 119.35, 119.28, 110.09, 97.05, 75.48, 72.85, 65.73, 61.47, 57.98, 49.71, 31.59, 30.78, 29.67, 26.52, 23.94, 23.16, 21.02, 19.61, 18.50, 18.30, 8.24.

Synthesis of intermediate 21-4

Compound 21-3 (0.62 g, 0.58 mmol) were added to DMF (20 mL) and stirred at RT. DEA (89.21 μL, 0.87 mmol) was added to the reaction vessel. The reaction mixture was stirred at RT for 5 h until TLC (DCM/MeOH 10/1) showed that compound 21-3 had been consumed. Compound 21-3A (226.74 μL, 0.58 mmol), HATU (0.26 g, 0.69 mmol), and DIPEA (0.15 mL, 0.87 mmol) were slowly added to the reaction vessel at RT. The reaction mixture was stirred at RT for 1 h until HPLC showed that compound 21-3A had been consumed. The reaction mixture was added dropwise to water (120 mL), and the yellow solid was gradually precipitated and filtered. The filter cake was washed with water (20 mL) and dried to obtain the crude product. The crude product was triturated using DCM (10 mL) to obtain a yellow solid (0.67 g, 57.78%). LC-MS (ESI^+) m/z 1247.2 $(M+H)^+$. ^1H NMR (400 MHz, CD_3OD) δ 7.51 (d, J = 7.7 Hz, 2H), 7.37 - 7.10 (m, 4H), 5.45 (d, J = 29.2 Hz, 1H), 5.38 (s, 1H), 5.23 (s, 1H), 5.19 (s, 1H), 4.77 (s, 1H), 4.53 (dd, J = 24.2, 15.1 Hz, 2H), 4.29 (d, J = 7.2 Hz, 1H), 3.92 (t, J = 6.8 Hz, 1H), 3.61 (s, 30H), 3.34 (s, 3H), 3.17 (t, J = 6.7 Hz, 2H), 2.79 (d, J = 27.1 Hz, 4H), 2.43 (t, J = 6.1 Hz, 2H), 2.25 - 1.67 (m, 7H), 1.48 (dq, J = 20.7, 13.6, 10.4 Hz, 7H), 0.97 (dt, J = 14.6, 7.1 Hz, 9H). ^{13}C NMR (101 MHz, CD_3OD) δ 173.18, 172.42, 171.70, 171.49, 171.03, 158.52, 157.25, 156.00, 150.80, 150.27, 145.69, 145.01 (d, J = 12.5 Hz), 142.26 (d, J = 13.7 Hz), 141.44, 138.57, 132.14, 129.26, 128.96, 124.87, 122.21, 119.50, 118.86, 110.85, 110.67, 97.65, 75.08, 72.69, 71.56, 70.63 - 69.69 (m), 66.96, 65.30, 62.40, 58.68, 57.75, 49.84, 38.74, 36.33, 30.93, 30.90, 30.71, 28.55, 26.17, 23.32, 22.73, 20.35, 18.48, 17.47, 16.77, 6.95.

Synthesis of intermediate 21-5

Compound 21-4 (0.6 g, 0.48 mmol) was added to a PBS solution (pH 6.0) (12 mL) and stirred at 0°C. $P(CH_3)_3$ (1 mol/L THF) (2.41 mL, 2.41 mmol) was slowly added to the reaction vessel. The reaction mixture was stirred at 0°C for 2 h until HPLC showed that compound 21-4 had been consumed. The reactant was dissolved in MeOH (20 mL) and cleaned up with a C18 Spherical 20 ~ 35 μm 100 Å cartridge. The mobile phase A was water + 0.1% FA, and the mobile phase B was ACN. The gradient range was 10 ~ 80 % B. The solvent was concentrated under reduced pressure to obtain a white solid (0.28 g, 47.66%). LC-MS (ESI^+) m/z 1221.2 $(M+H)^+$. ^1H NMR (400 MHz, DMSO-d_6) δ 10.07 (s, 1H), 8.32 (d, J = 25.7, 2H), 8.14 (d, J = 8.6, 1H), 7.83 (d, J = 12.8, 2H), 7.60 (d, J = 8.1, 2H), 7.39 (d, J = 8.2, 2H), 7.29 (s, 1H), 5.42 (s, 2H), 5.15 (d, J = 39.1, 4H), 4.38 (q, J = 7.2, 1H), 4.25 (d, J = 6.8, 1H), 3.69 - 3.30 (m, 31H), 3.23 (s, 3H), 3.04 (dt, J = 34.9, 6.2, 6H), 2.29 (t, J = 6.6, 2H), 2.12 - 1.16 (m, 14H), 1.05 - 0.68 (m, 9H). ^{13}C NMR (101 MHz, DMSO-d_6) δ 172.89, 171.70, 171.16, 171.20, 171.02, 156.95, 155.78, 154.33, 151.40, 150.73, 145.67, 145.09 (d, J = 16.1 Hz), 142.05 (d, J = 13.6 Hz, 141.16, 139.39, 131.71, 129.45, 129.41, 125.29, 122.28, 119.36, 119.12, 111.40, 109.76, 97.22, 75.23, 72.78, 71.62, 70.34 - 69.77 (m), 67.15, 65.89, 62.63, 58.42, 58.16, 49.77, 40.15, 39.94, 39.73, 39.52, 39.31, 39.10, 38.89, 36.51, 32.56, 31.08, 31.01, 30.87, 29.04, 26.37, 23.67, 22.46, 20.61, 19.52, 18.46, 18.16, 8.07.

Preparation of compound LP2004

Compound 21-5 (0.10 g, 81.94 μmol) was added to DMF (1 mL) and ACN (2 mL) and stirred at RT. Compound 21-5A (75.78 mg, 0.25 mmol) and TEA (34.17 μL, 0.25 mmol) were added to the reaction vessel at 0°C. The reaction mixture was stirred under N_2 atmosphere for 0.5 h until HPLC showed that compound 21-5 had been consumed. The reaction mixture was concentrated under reduced pressure to obtain the crude product. The crude product was dissolved in

DMSO (5 mL) and cleaned up with a C18 Spherical 20 ~ 35 μm 100 Å cartridge. The mobile phase A was water + 0.1 % FA, and the mobile phase B was ACN. The gradient range was 10 ~ 50 % B. The solvent was concentrated under reduced pressure to obtain a white solid (62 mg, 53.53%). HRMS (ES$^+$) m/z 1435.6677 (M+Na)$^+$. ^{1}H NMR (400 MHz, DMSO-*d*$_6$) *δ* 10.02 (s, 1H), 8.18 (d, *J* = 6.6 Hz, 1H), 7.96 (d, *J* = 7.9 Hz, 1H), 7.80 (h, *J* = 4.2 Hz, 2H), 7.63 (dd, *J* = 21.5, 8.4 Hz, 3H), 7.45 - 7.22 (m, 3H), 7.00 (s, 2H), 6.52 (s, 1H), 5.57 - 4.83 (m, 6H), 4.44 - 4.13 (m, 3H), 3.61 - 3.34 (m, 32H), 3.23 (d, *J* = 3.0 Hz, 3H), 3.14 - 2.86 (m, 6H), 2.37 - 1.00 (m, 24H), 0.85 (dq, *J* = 24.3, 9.0, 8.1 Hz, 9H). ^{13}C NMR (101 MHz, DMSO-*d*$_6$) *δ* 172.93, 172.67, 172.33, 171.59, 171.53, 171.09, 170.25, 159.12, 156.33, 155.56, 151.87, 150.48, 146.30, 146.05 (d, *J* = 12.1 Hz), 142.36 (d, *J* = 14.5 Hz), 139.63, 136.85, 134.90, 131.66, 129.91, 129.82, 125.34, 123.02, 119.38, 119.34, 112.23, 112.11, 97.06, 75.52, 72.84, 71.74, 70.45 - 69.89 (m), 67.33, 65.73, 62.80, 58.51, 57.71, 53.00, 49.57, 40.60, 40.52, 40.39, 40.19, 39.98, 39.77, 39.56, 39.44, 39.35, 38.79, 36.61, 35.41, 31.77, 31.19, 30.72, 29.32, 28.25, 26.61, 26.24, 25.28, 23.34, 21.08, 19.60, 18.42, 18.37, 8.24.

Scheme 3. Synthesis of LP3004

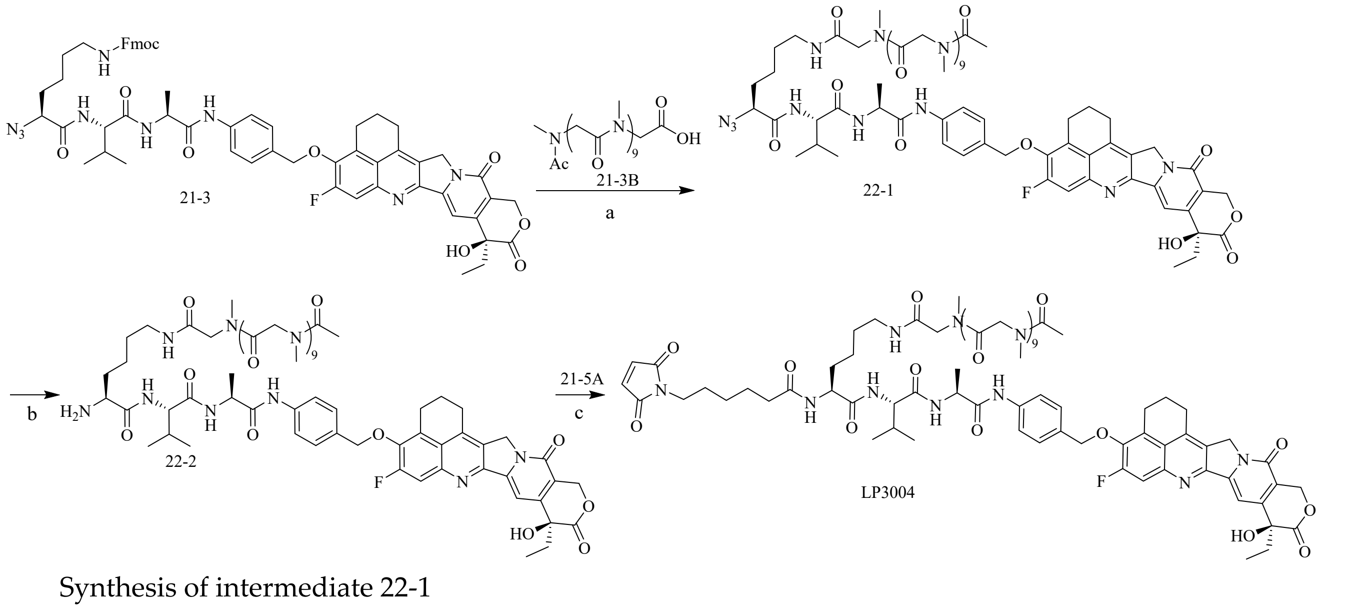

Synthesis of intermediate 22-1

Compound 21-3 (0.62 g, 0.58 mmol) were added to DMF (20 mL) and stirred at RT. DEA (89.21 μL, 0.87 mmol) was added to the reaction vessel. The reaction mixture was stirred at RT for 5 h until TLC (DCM/MeOH 10/1) showed that compound 21-3 had been consumed. Compound 21-3B (0.72 g, 930.94 μmol), HATU (0.71 g, 1.86 mmol), and DIPEA (0.32 mL, 1.86 mmol) were slowly added to the reaction vessel at RT. The reaction mixture was stirred at RT for 1 h until HPLC showed that compound 21-3B had been consumed. The reaction mixture was diluted with H_2O (200 mL) and extracted with DCM/MeOH (10:1) (3 × 250 mL). The organic phase was collected, dried with anhydrous sodium sulfate, and filtered. The filtrate was concentrated under reduced pressure to obtain a yellow solid (1.28 g, 85.91%). LC-MS (ESI$^+$) m/z 1605.5 (M+H)$^+$. ^{1}H NMR (400 MHz, DMSO-*d*$_6$) *δ* 10.06 (s, 1H), 8.34 (d, *J* = 6.5 Hz, 1H), 8.19 (d, *J* = 8.4 Hz, 1H), 7.82 (t, *J* = 15.1 Hz, 2H), 7.60 (d, *J* = 8.0 Hz, 2H), 7.39 (d, *J* = 8.1 Hz, 2H), 7.28 (s, 1H), 6.53 (s, 1H), 5.42 (s, 2H), 5.12 (d, *J* = 28.5 Hz, 4H), 4.52 - 3.79 (m, 23H), 3.25 - 2.67 (m, 36H), 2.51 (s, 6H), 2.19 - 1.16 (m, 17H), 0.87 (dd, *J* = 19.1, 7.4 Hz, 9H). ^{13}C NMR (101 MHz, DMSO-*d*$_6$) *δ* 172.96, 171.23, 169.87 - 168.25 (m), 157.01, 155.86, 152.06, 150.82, 150.39, 145.80, 145.16, 142.06, 141.35, 139.43, 131.80, 129.54, 129.53, 125.48, 122.33, 119.37, 119.13, 113.18, 111.28, 97.21, 75.24, 72.82, 65.35, 62.95, 58.16, 53.21, 50.66 - 48.89 (m), 37.20, 36.37 - 35.11 (m), 32.45, 30.92, 30.76, 28.97, 26.30, 23.19, 22.22, 21.57, 19.50, 18.45, 18.14, 8.07.

Synthesis of intermediate 22-2

Compound 22-1 (0.50 g, 0.31 mmol) was added to a PBS solution (pH 6.0) (12 mL) and stirred at 0°C. $P(CH_3)_3$ (1 mol/L THF) (1.56 mL, 1.56 mmol) was slowly added to the reaction vessel. The reaction mixture was stirred at 0°C for 2 h until HPLC showed that compound 22-1 had been consumed. The reactant was dissolved in MeOH (20 mL) and cleaned up with a C18 Spherical 20 ~ 35 μm 100 Å cartridge. The mobile phase A was water + 0.1 %FA, and the mobile phase B was ACN. The gradient range was 10 ~ 80% B. The solvent was concentrated under reduced pressure to obtain

a white solid (150 mg, 30.49%). LC-MS (ESI$^+$) m/z 1578.9 (M+H)$^+$. ^{1}H NMR (400 MHz, DMSO-*d*$_6$) δ 10.07 (s, 1H), 8.42 - 8.25 (m, 2H), 8.10 (s, 1H), 7.84 (d, *J* = 12.2 Hz, 1H), 7.60 (d, *J* = 8.0 Hz, 2H), 7.39 (d, *J* = 8.1 Hz, 2H), 7.29 (s, 1H), 5.59 - 4.90 (m, 6H), 4.51 - 3.81 (m, 22H), 3.29 - 2.57 (m, 37H), 2.07 - 1.18 (m, 17H), 0.99 - 0.71 (m, 9H). ^{13}C NMR (101 MHz, DMSO-*d*$_6$) δ 172.88, 171.67, 170.99, 170.33 - 167.76 (m), 156.94, 155.87, 154.82, 150.94, 150.23, 145.74, 142.15, 141.09, 139.46, 131.68, 129.56, 129.28, 125.41, 122.25, 119.24, 119.08, 112.04, 111.34, 96.96, 77.23, 72.81, 64.81, 61.52, 58.06, 52.77 - 47.24 (m), 37.18, 36.30 - 34.65 (m), 31.18, 30.99, 30.81, 28.98, 27.50, 23.83, 23.13, 21.56, 19.50, 18.37, 8.11.

Preparation of compound LP3004

Compound 22-2 (0.10 g, 63.34 μmol) was added to DMF (1 mL) and ACN (2 mL) and stirred at RT. Succinimide 6-(maleimide) caproate (58.58 mg, 0.19 mmol) and TEA (26.41 μL, 0.19 mmol) were added to the reaction vessel at 0°C. The reaction mixture was stirred under N_2 atmosphere for 0.5 h until HPLC showed that compound 22-2 had been consumed. The reaction mixture was concentrated under reduced pressure to obtain a crude product. The crude product was dissolved in DMSO (5 mL) and cleaned up with a C18 Spherical 20 ~ 35 μm 100 Å cartridge. The mobile phase A was water + 0.1% FA, and the mobile phase B was ACN. The gradient range was 10 ~ 50% B. The solvent was concentrated under reduced pressure to obtain a white solid (52 mg, 46.33%). HRMS (ES$^+$) m/z 1793.8606 (M+Na)$^+$. ^{1}H NMR (400 MHz, DMSO-*d*$_6$) δ 10.04 (s, 1H), 8.29 (d, *J* = 49.7 Hz, 2H), 8.09 - 7.91 (m, 1H), 7.82 (h, *J* = 7.6, 7.1 Hz, 1H), 7.62 (dd, *J* = 19.3, 12.9 Hz, 3H), 7.50 - 7.18 (m, 3H), 6.99 (s, 2H), 5.66 - 4.85 (m, 6H), 4.50 - 3.85 (m, 23H), 3.18 - 2.66 (m, 38H), 2.18 - 1.08 (m, 25H), 0.84 (tt, *J* = 20.0, 9.9 Hz, 9H).

Copies of selected ^{1}H NMR, ^{13}C NMR, and Mass Spectra

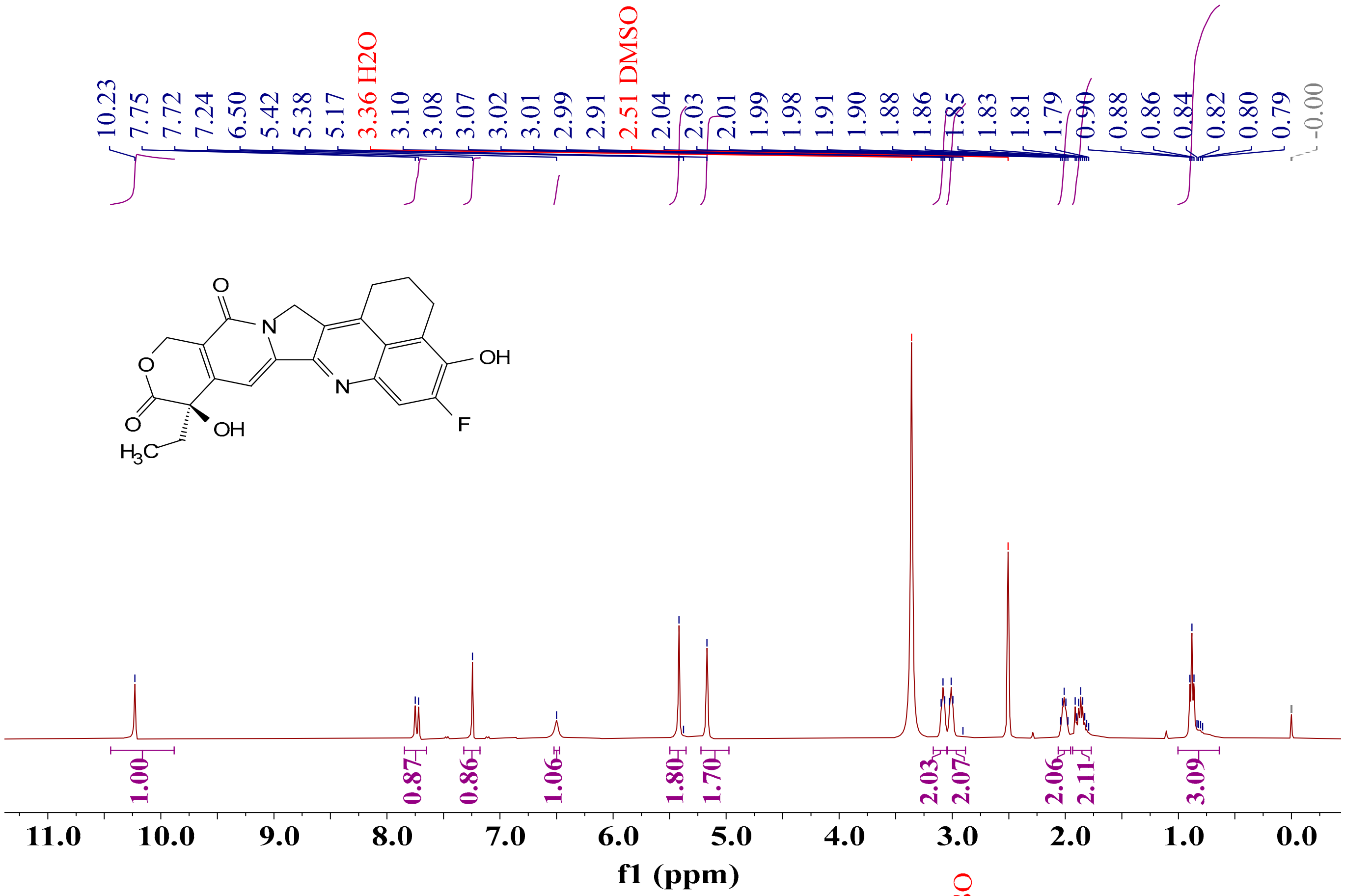

10.23 7.75 7.72 7.24 6.50 5.42 5.38 5.17 3.36 H2O 3.10 3.08 3.07 3.02 3.01 2.99 2.91 2.51 DMSO 2.04 2.03 2.01 1.99 1.98 1.91 1.90 1.88 1.86 1.85 1.83 1.81 1.79 0.90 0.88 0.86 0.84 0.82 0.80 0.79 -0.00
OH
F
N
O
OH
H3C
1.00 0.87 0.86 1.06 1.80 1.70 2.03 2.07 2.06 2.11 3.09
11.0 10.0 9.0 8.0 7.0 6.0 5.0 4.0 3.0 2.0 1.0 0.0
f1 (ppm)

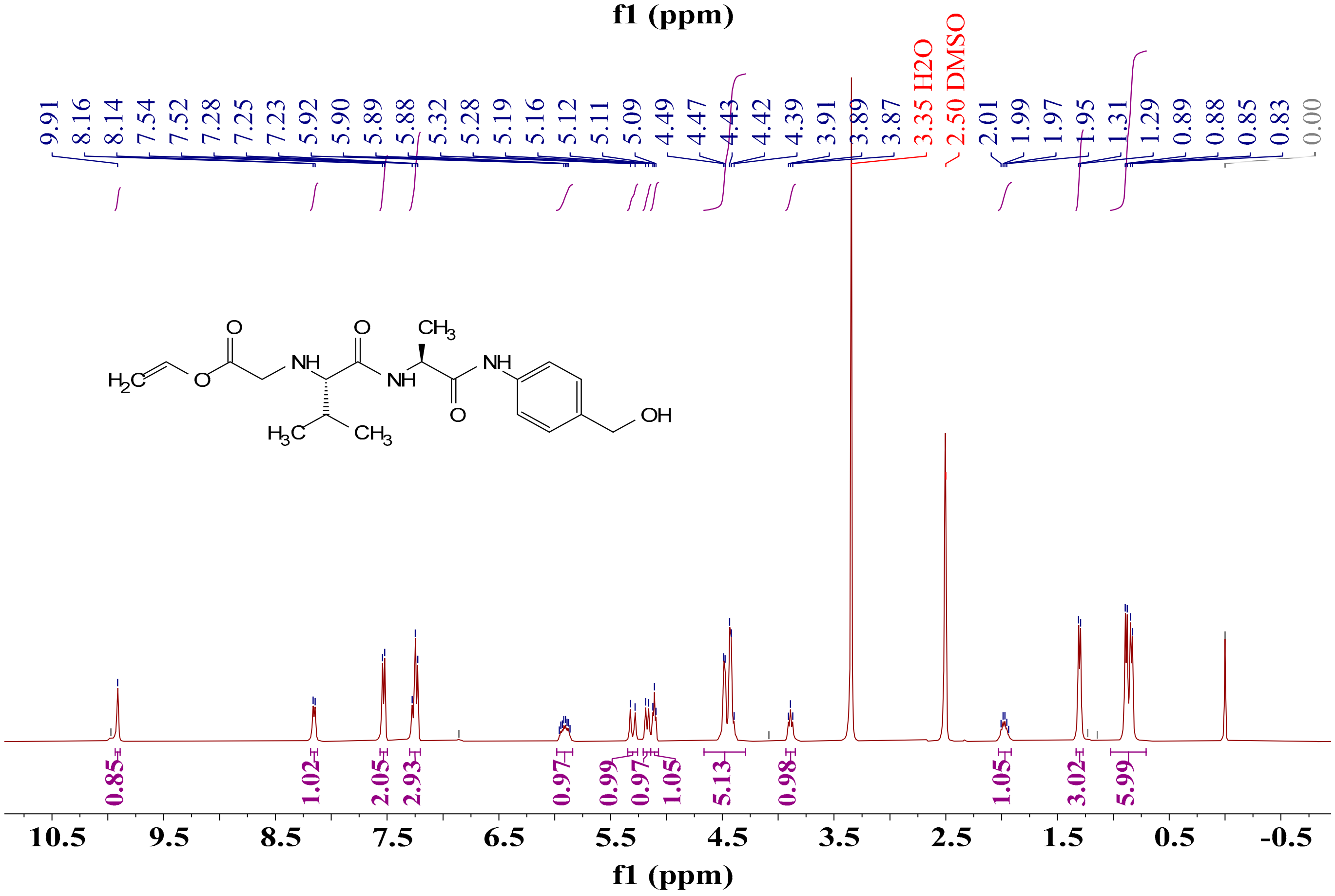

9.91 8.16 8.14 7.54 7.52 7.28 7.25 7.23 5.92 5.90 5.89 5.88 5.32 5.28 5.19 5.16 5.12 5.11 5.09 4.49 4.47 4.43 4.42 4.39 3.91 3.89 3.87 3.35 H2O 2.50 DMSO 2.01 1.99 1.97 1.95 1.31 1.29 0.89 0.88 0.85 0.83 0.00
H2C
O
NH
CH3
OH
H3C
CH3
0.85 1.02 2.05 2.93 0.97 0.99 0.97 1.05 5.13 0.98 1.05 3.02 5.99
10.5 9.5 8.5 7.5 6.5 5.5 4.5 3.5 2.5 1.5 0.5 -0.5
f1 (ppm)

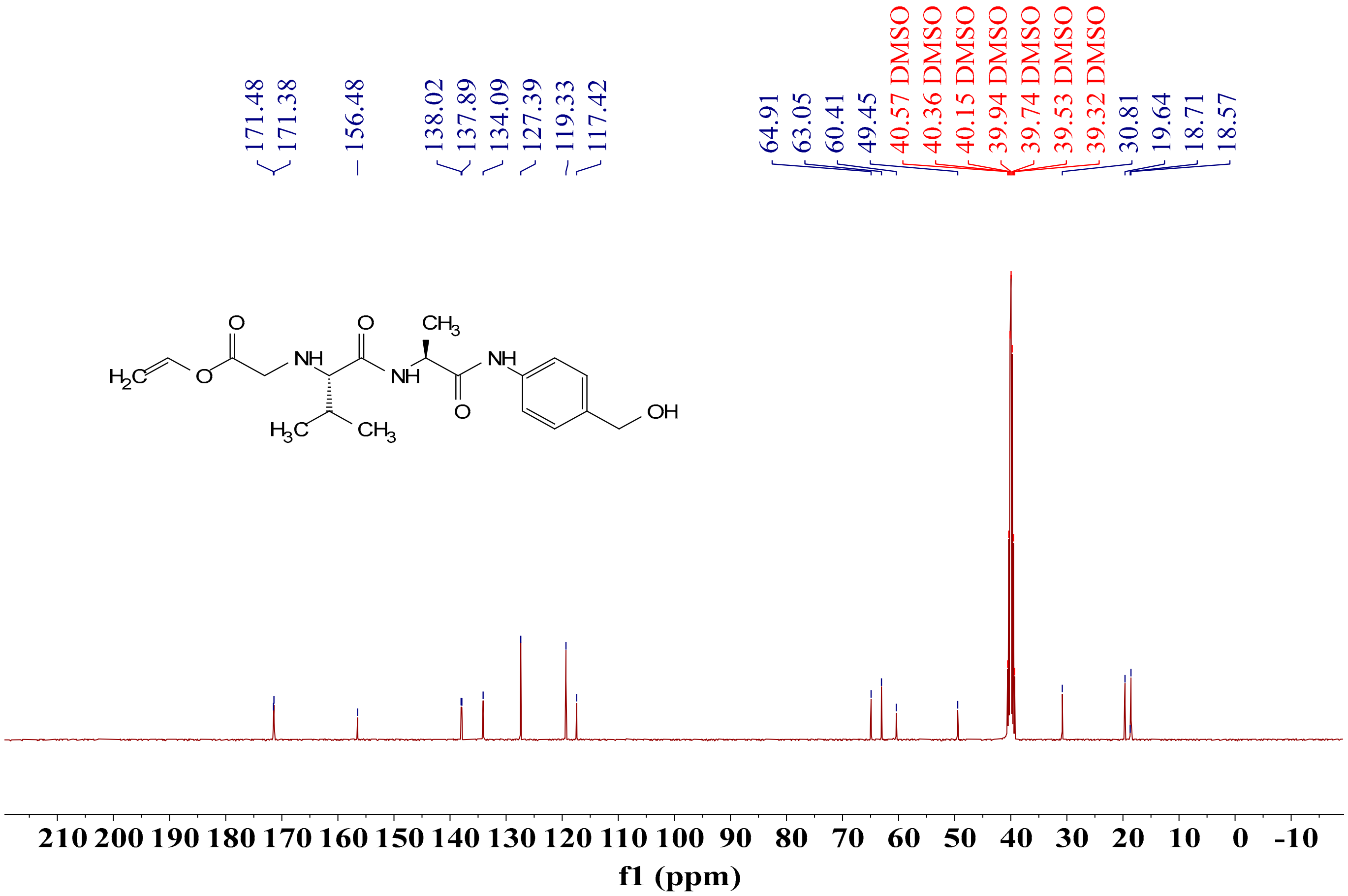
171.48
171.38
156.48
138.02
137.89
134.09
127.39
119.33
117.42
64.91
63.05
60.41
49.45
40.57 DMSO
40.36 DMSO
40.15 DMSO
39.94 DMSO
39.74 DMSO
39.53 DMSO
39.32 DMSO
30.81
19.64
18.71
18.57
210 200 190 180 170 160 150 140 130 120 110 100 90 80 70 60 50 40 30 20 10 0 -10
f1 (ppm)

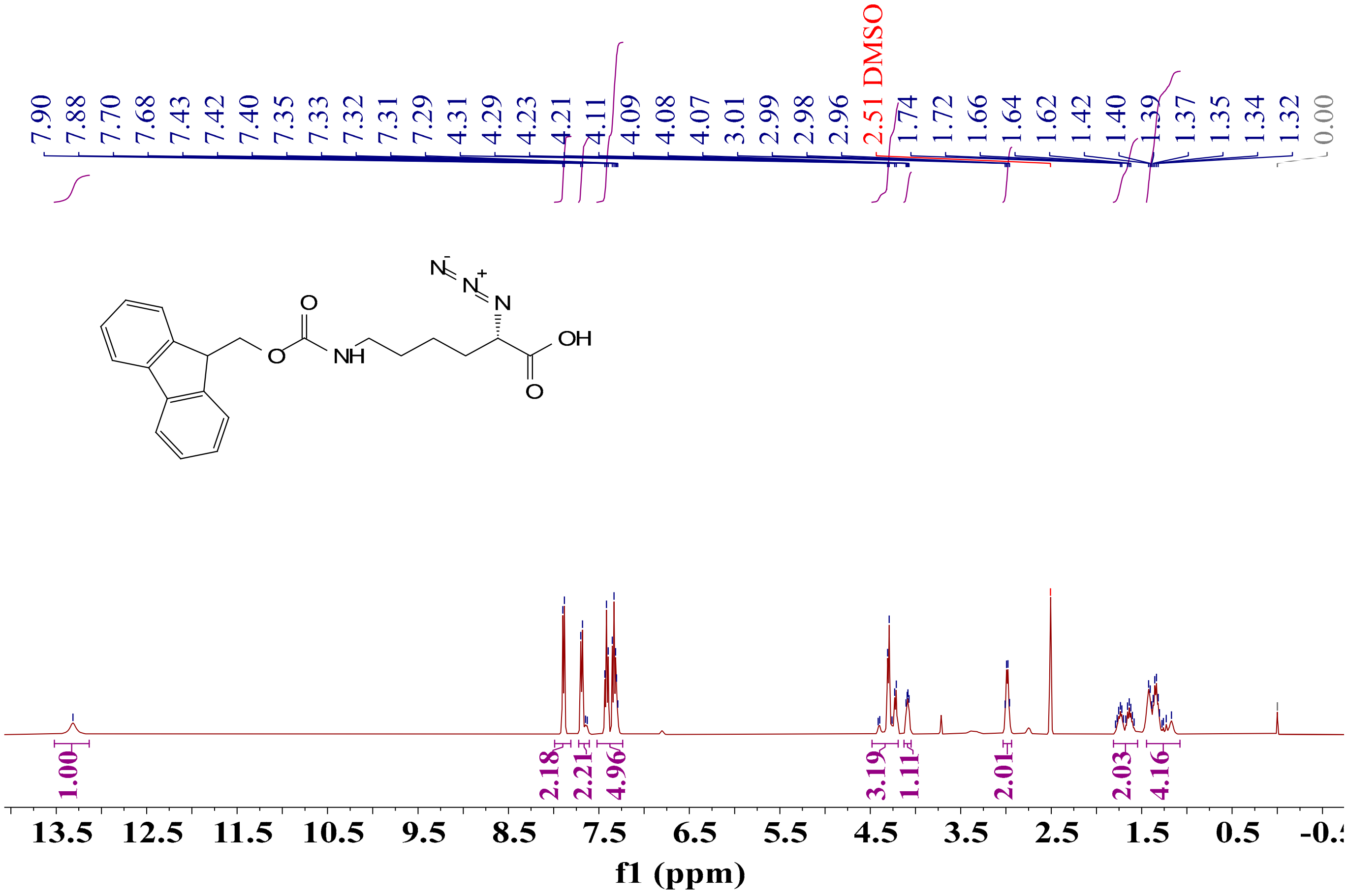
7.90
7.88
7.70
7.68
7.43
7.42
7.40
7.35
7.33
7.32
7.31
7.29
4.31
4.29
4.23
4.21
4.11
4.09
4.08
4.07
3.01
2.99
2.98
2.96
2.51 DMSO
1.74
1.72
1.66
1.64
1.62
1.42
1.40
1.39
1.37
1.35
1.34
1.32
0.00
1.00
2.18
2.21
4.96
3.19
1.11
2.01
2.03
4.16
13.5 12.5 11.5 10.5 9.5 8.5 7.5 6.5 5.5 4.5 3.5 2.5 1.5 0.5
f1 (ppm)

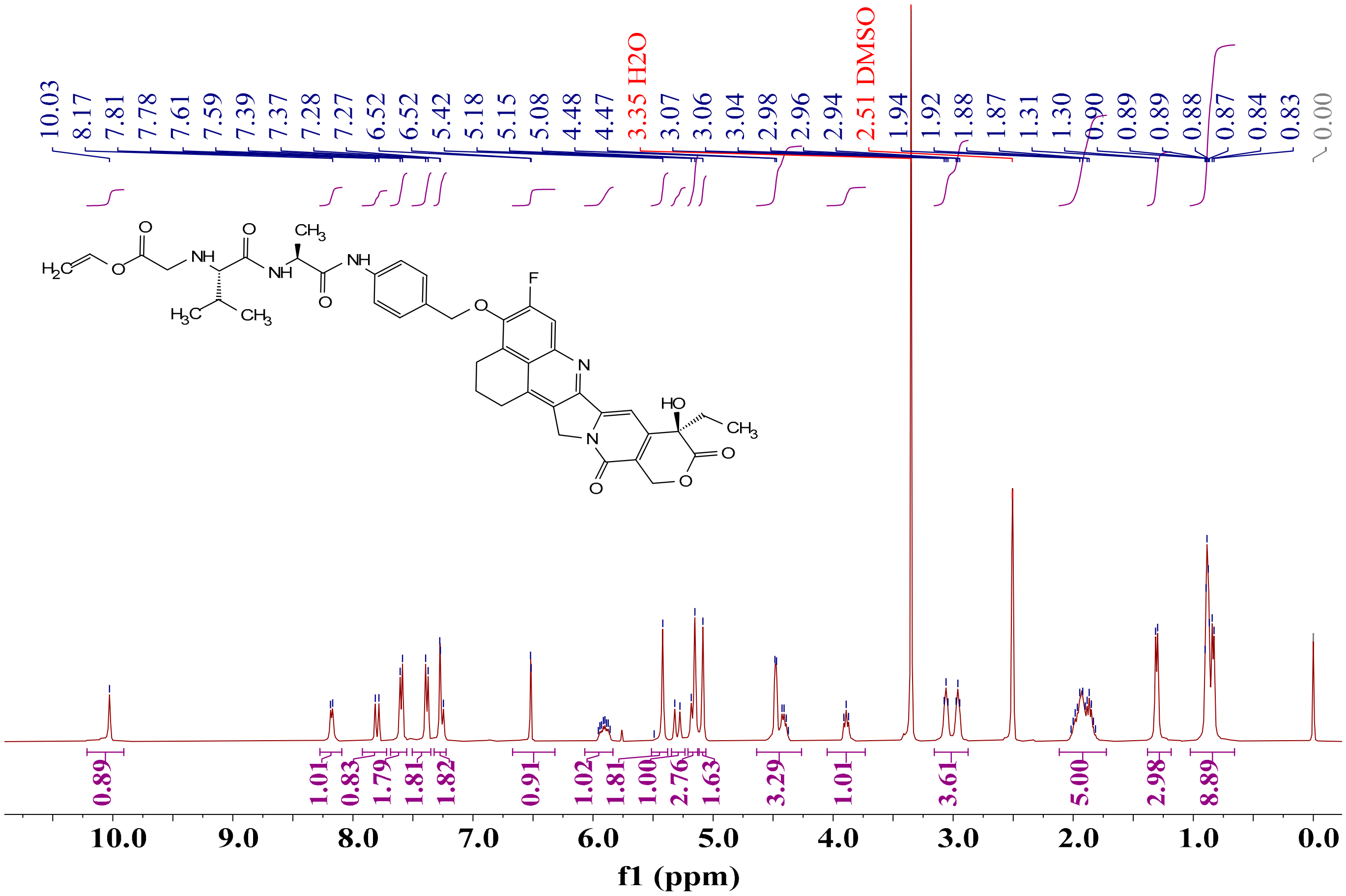

10.03
8.17
7.81
7.78
7.61
7.59
7.39
7.37
7.28
7.27
6.52
6.52
5.42
5.18
5.15
5.08
4.48
4.47
3.35 H2O
3.07
3.06
3.04
2.98
2.96
2.94
2.51 DMSO
1.94
1.92
1.88
1.87
1.31
1.30
0.90
0.89
0.89
0.88
0.87
0.84
0.83
0.00
0.89
1.01
0.83
1.79
1.81
1.82
0.91
1.02
1.81
1.00
2.76
1.63
3.29
1.01
3.61
5.00
2.98
8.89
10.0
9.0
8.0
7.0
6.0
5.0
4.0
3.0
2.0
1.0
0.0
f1 (ppm)

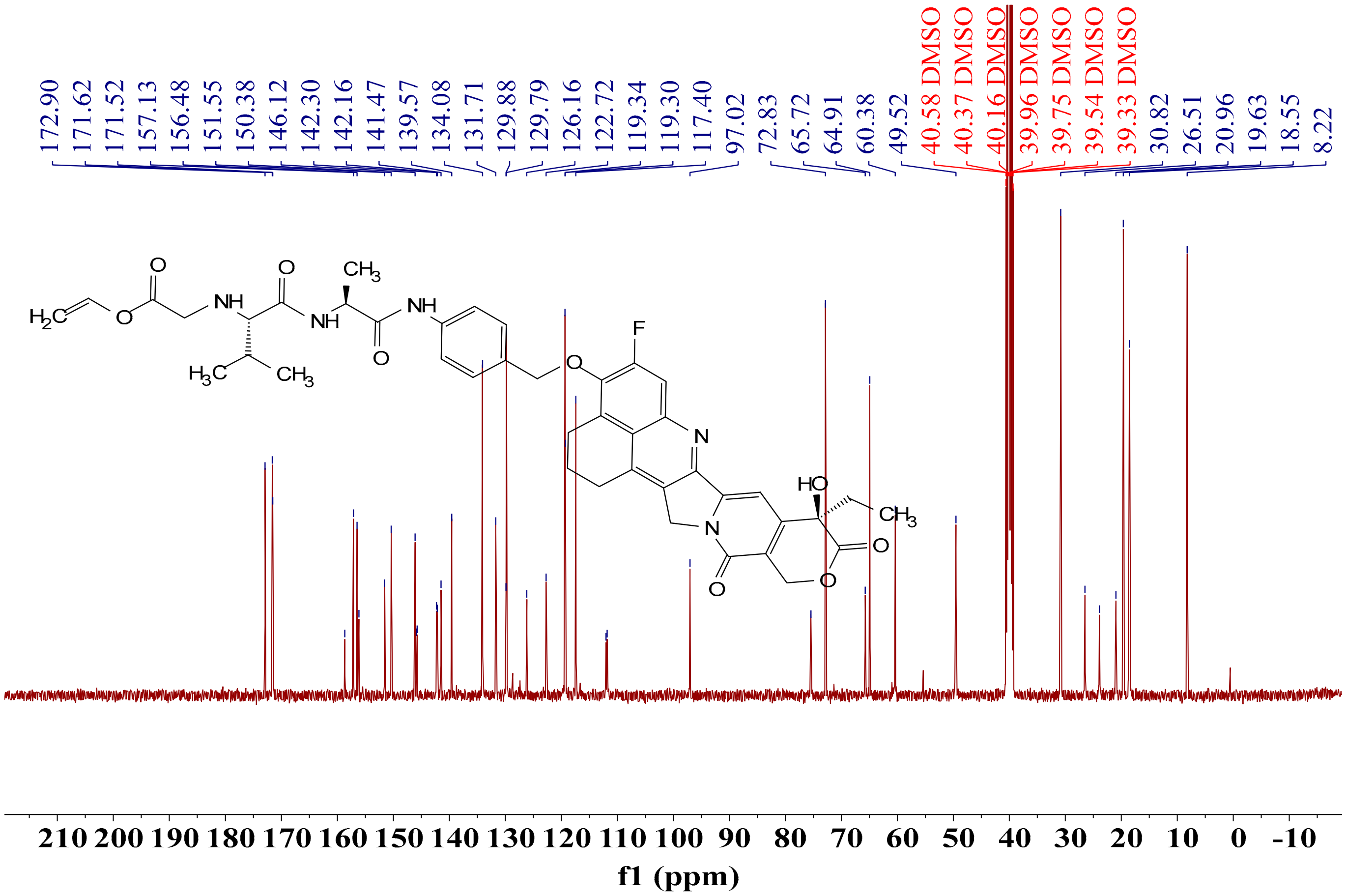

172.90
171.62
171.52
157.13
156.48
151.55
150.38
146.12
142.30
142.16
141.47
139.57
134.08
131.71
129.88
129.79
126.16
122.72
119.34
119.30
117.40
97.02
72.83
65.72
64.91
60.38
49.52
40.58 DMSO
40.37 DMSO
40.16 DMSO
39.96 DMSO
39.75 DMSO
39.54 DMSO
39.33 DMSO
30.82
26.51
20.96
19.63
18.55
8.22
210 200 190 180 170 160 150 140 130 120 110 100 90 80 70 60 50 40 30 20 10 0 -10
f1 (ppm)

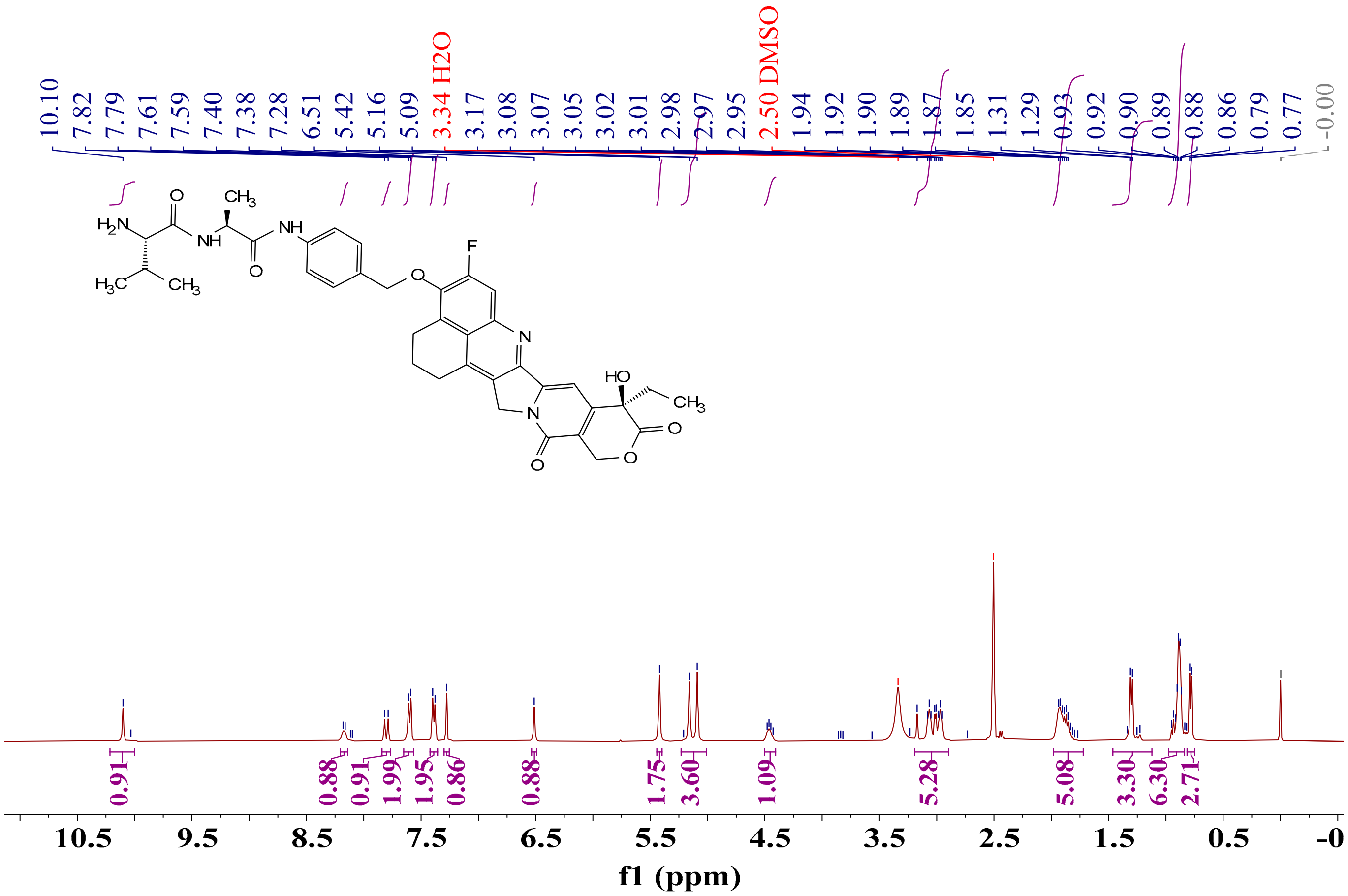
10.10 7.82 7.79 7.61 7.59 7.40 7.38 7.28 6.51 5.42 5.16 5.09 3.34 H2O 3.17 3.08 3.07 3.05 3.02 3.01 2.98 2.97 2.95 2.50 DMSO 1.94 1.92 1.90 1.89 1.87 1.85 1.31 1.29 0.93 0.92 0.90 0.89 0.88 0.86 0.79 0.77 -0.00
0.91 0.88 0.91 1.99 1.95 0.86 0.88 1.75 3.60 1.09 5.28 5.08 3.30 6.30 2.71
10.5 9.5 8.5 7.5 6.5 5.5 4.5 3.5 2.5 1.5 0.5 -0
f1 (ppm)

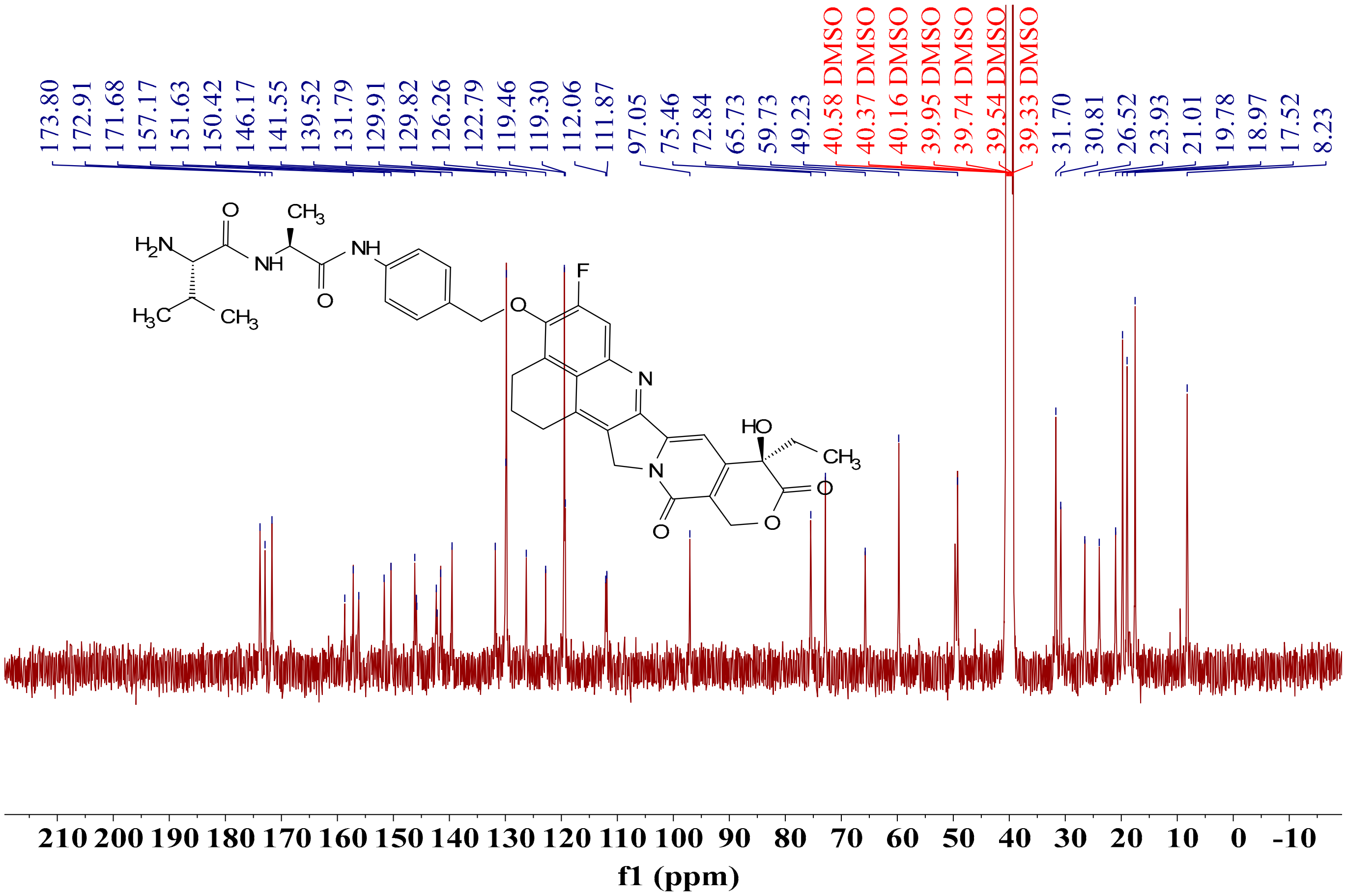
173.80 172.91 171.68 157.17 151.63 150.42 146.17 141.55 139.52 131.79 129.91 129.82 126.26 122.79 119.46 119.30 112.06 111.87 97.05 75.46 72.84 65.73 59.73 49.23 40.58 DMSO 40.37 DMSO 40.16 DMSO 39.95 DMSO 39.74 DMSO 39.54 DMSO 39.33 DMSO 31.70 30.81 26.52 23.93 21.01 19.78 18.97 17.52 8.23
210 200 190 180 170 160 150 140 130 120 110 100 90 80 70 60 50 40 30 20 10 0 -10
f1 (ppm)

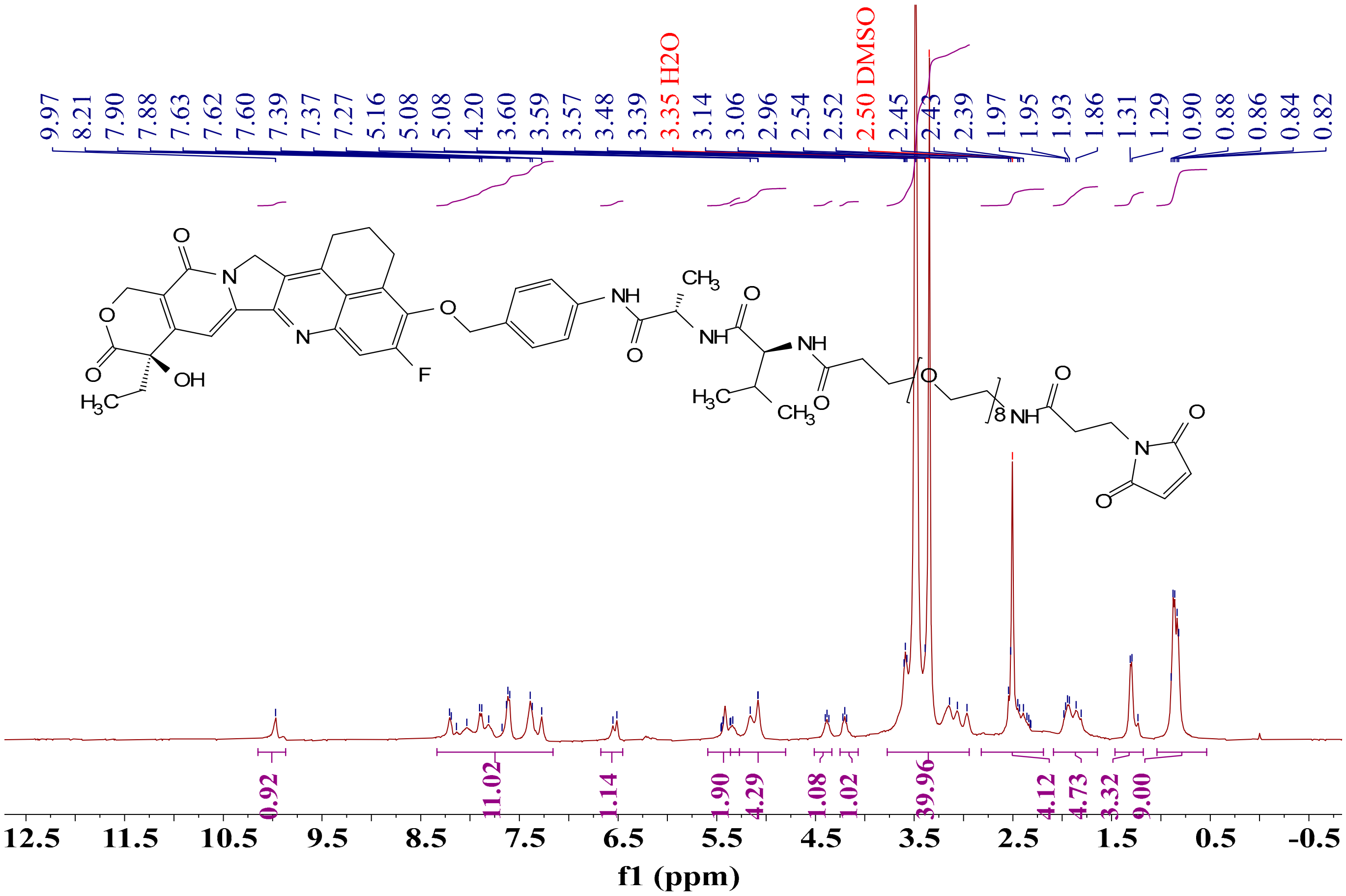

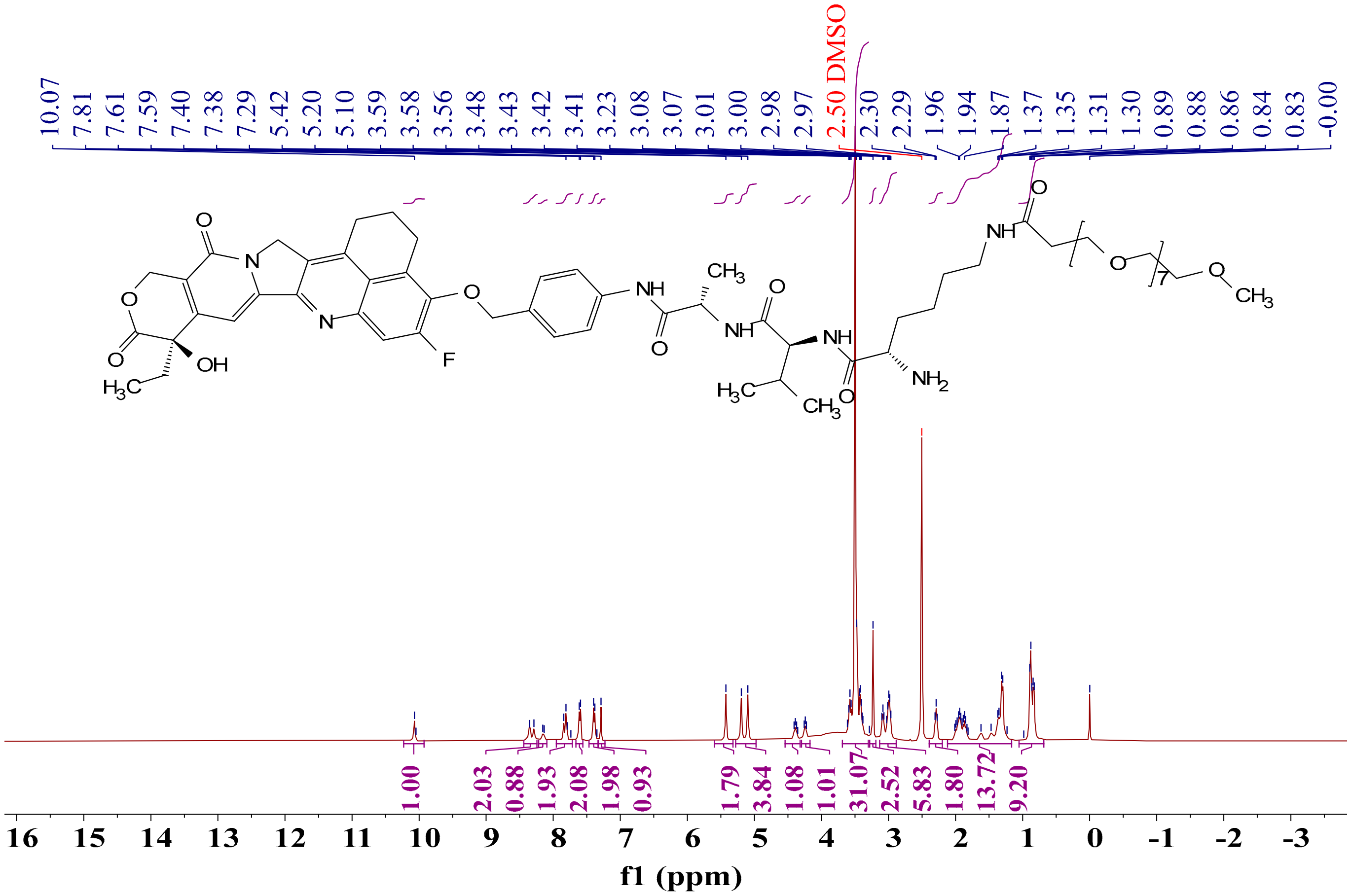

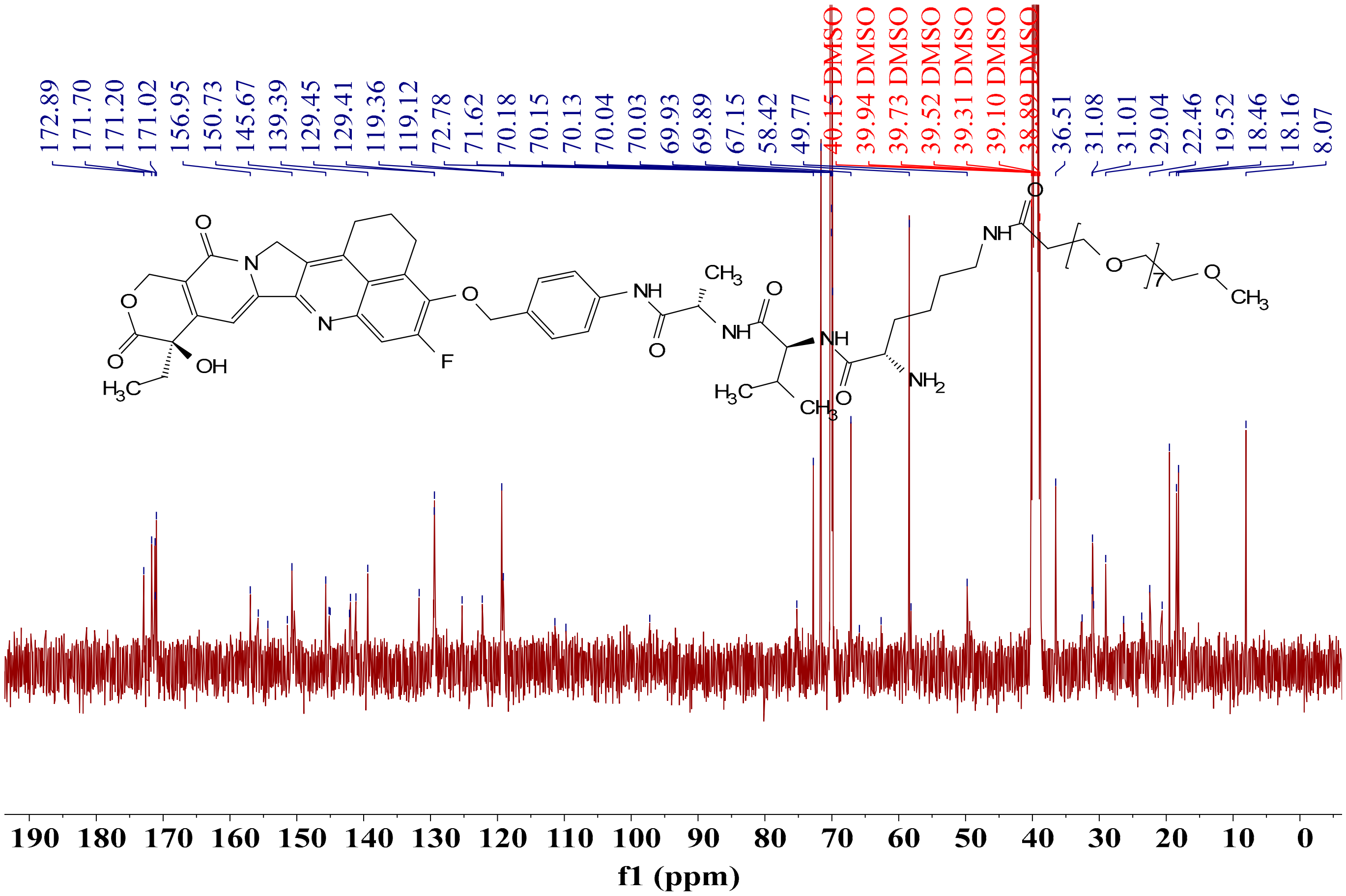

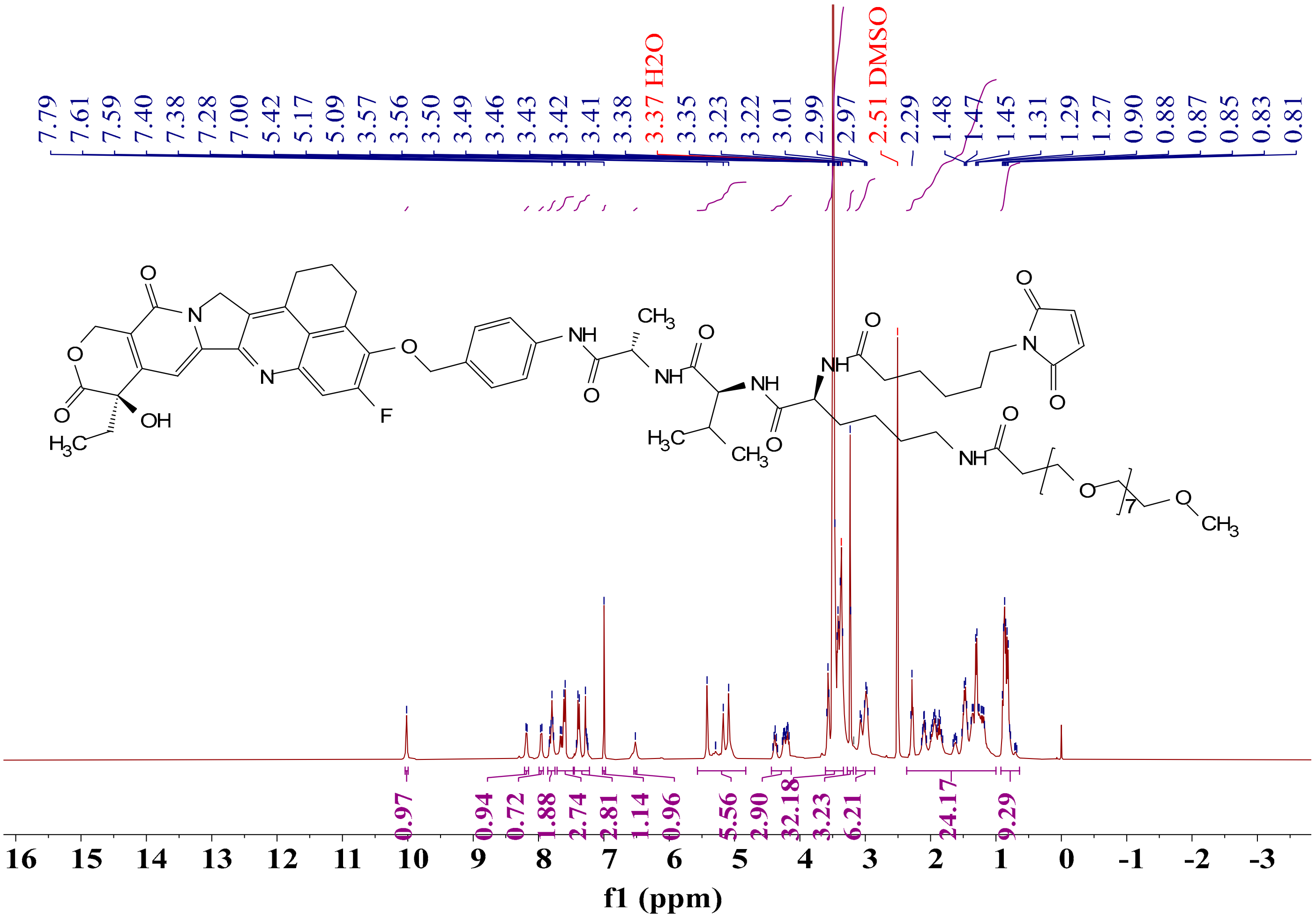

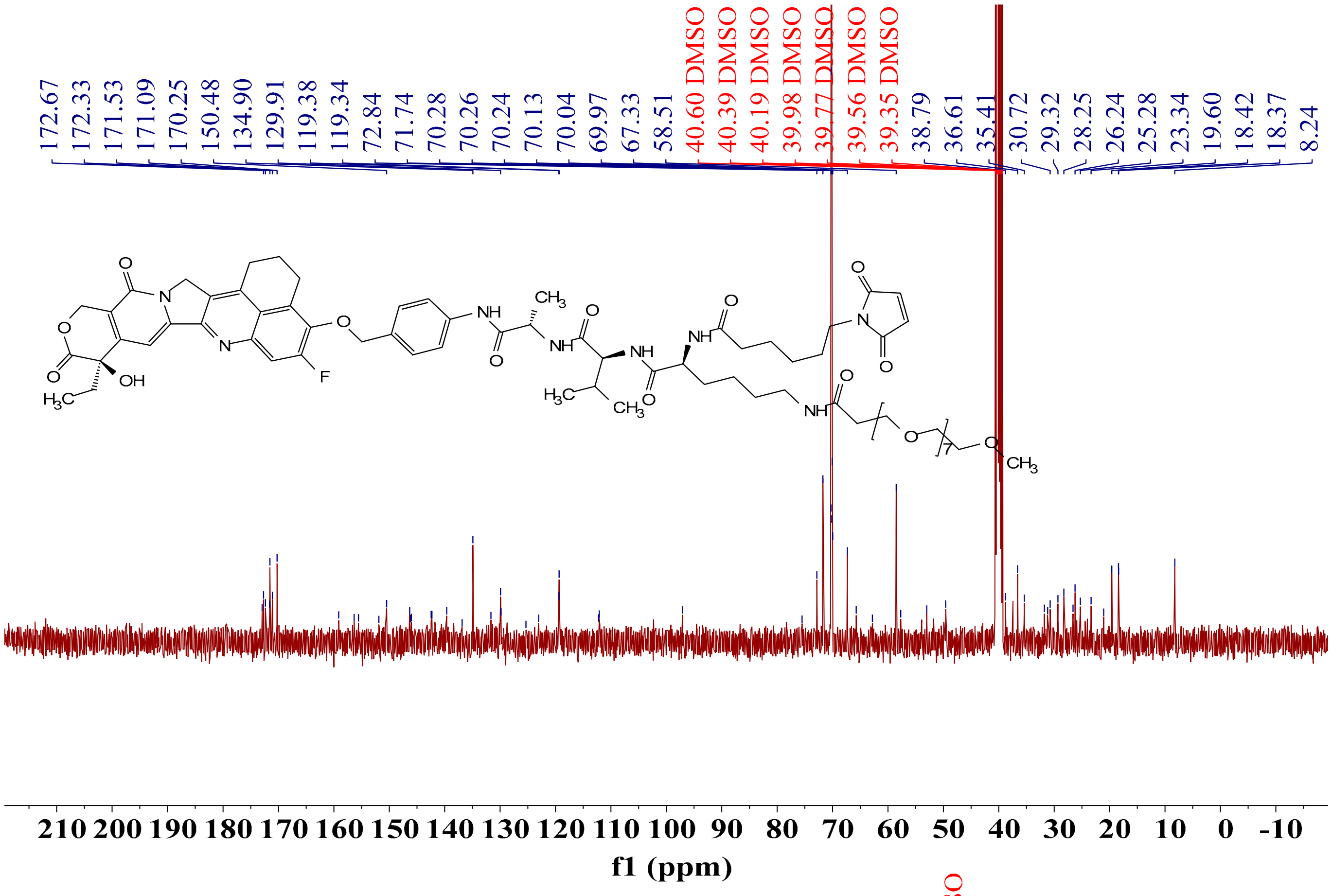

172.67
172.33
171.53
171.09
170.25
150.48
134.90
129.91
119.38
119.34
72.84
71.74
70.28
70.26
70.24
70.13
70.04
69.97
67.33
58.51
40.60 DMSO
40.39 DMSO
40.19 DMSO
39.98 DMSO
39.77 DMSO
39.56 DMSO
39.35 DMSO
38.79
36.61
35.41
30.72
29.32
28.25
26.24
25.28
23.34
19.60
18.42
18.37
8.24
210 200 190 180 170 160 150 140 130 120 110 100 90 80 70 60 50 40 30 20 10 0 -10
f1 (ppm)

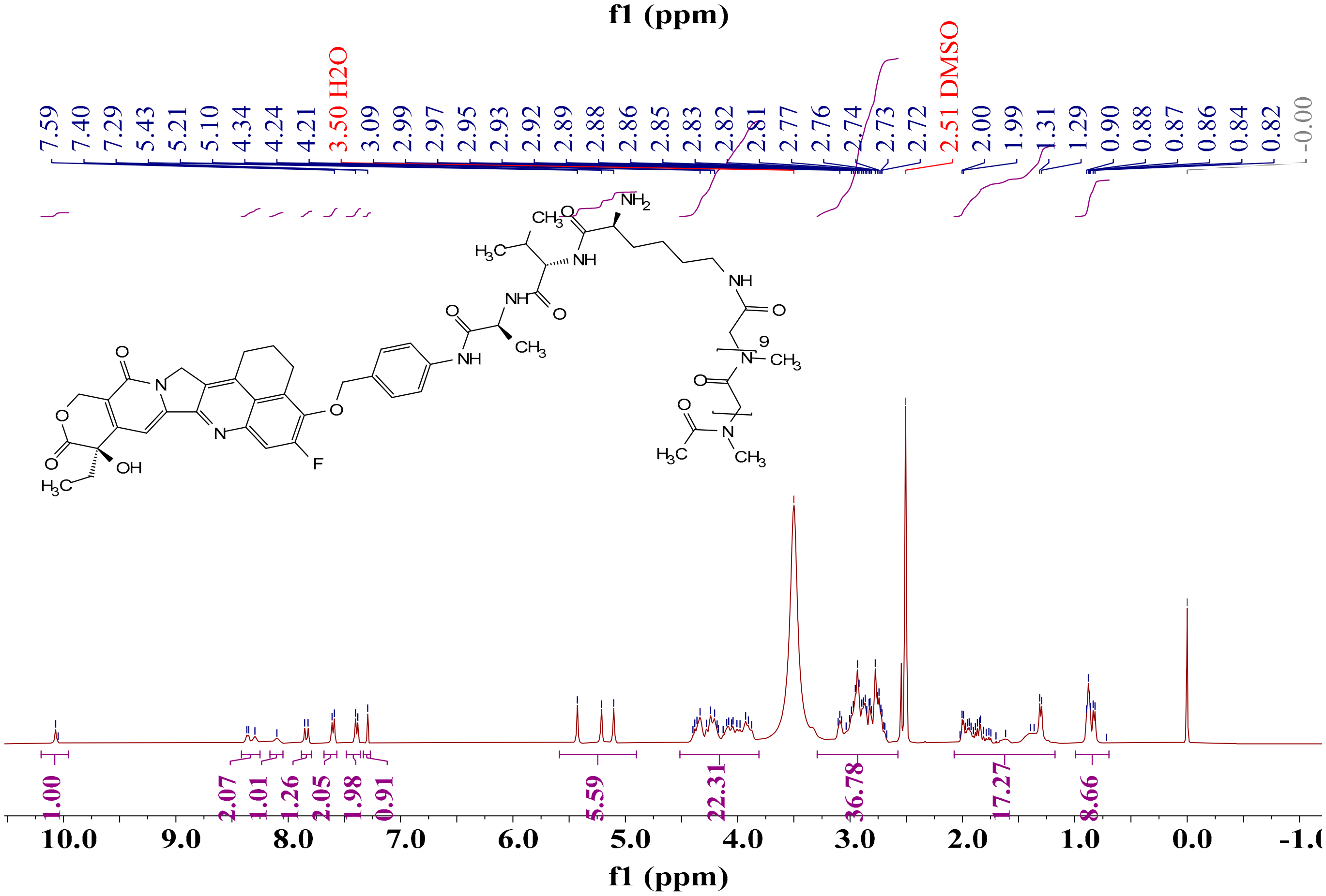

7.59
7.40
7.29
5.43
5.21
5.10
4.34
4.24
4.21
3.50 H2O
3.09
2.99
2.97
2.95
2.93
2.92
2.89
2.88
2.86
2.85
2.83
2.82
2.81
2.77
2.76
2.74
2.73
2.72
2.51 DMSO
2.00
1.99
1.31
1.29
0.90
0.88
0.87
0.86
0.84
0.82
-0.00
1.00
2.07
1.01
1.26
2.05
1.98
0.91
5.59
22.31
36.78
17.27
8.66
10.0 9.0 8.0 7.0 6.0 5.0 4.0 3.0 2.0 1.0 0.0
f1 (ppm)

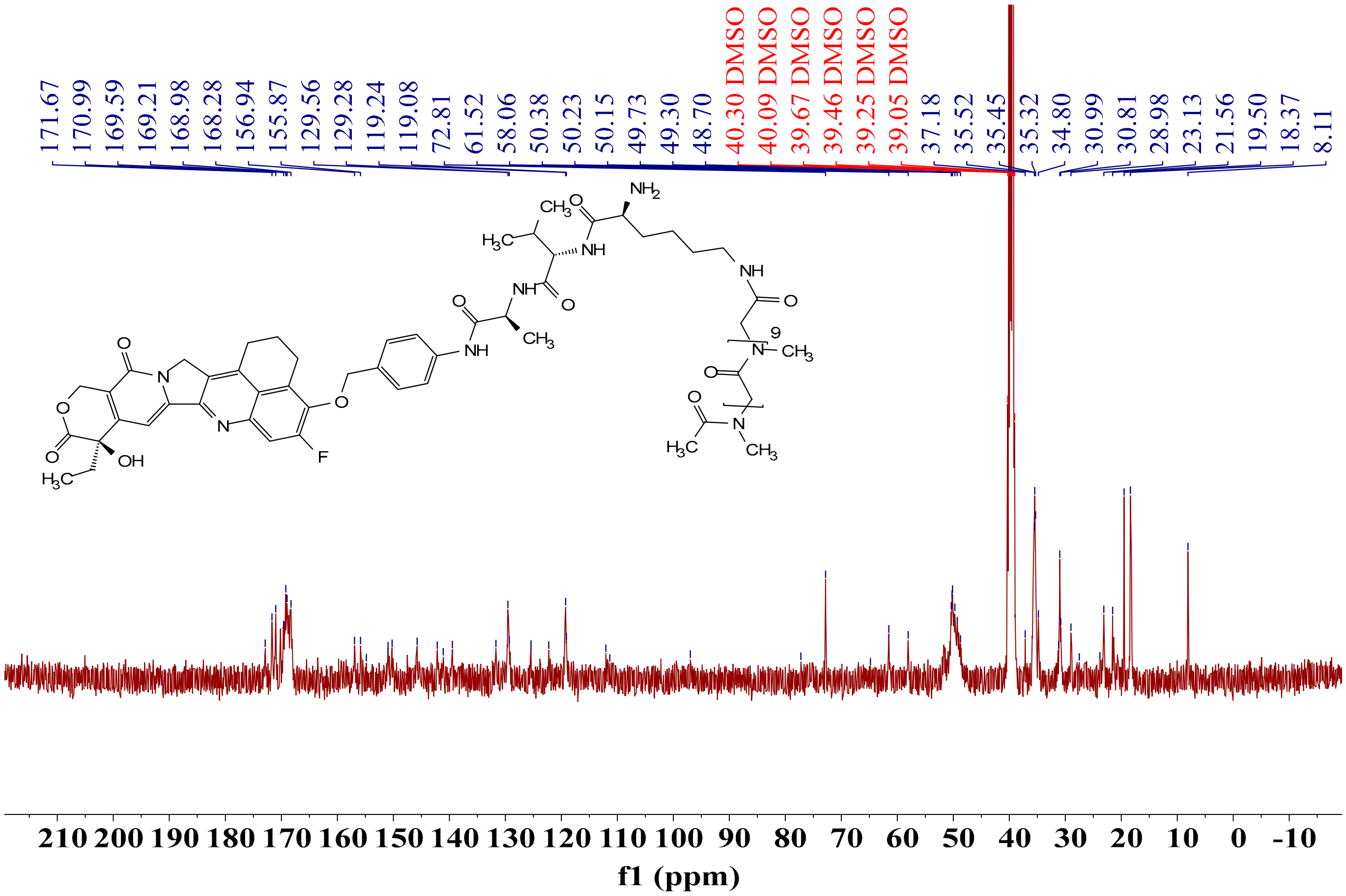
171.67
170.99
169.59
169.21
168.98
168.28
156.94
155.87
129.56
129.28
119.24
119.08
72.81
61.52
58.06
50.38
50.23
50.15
49.73
49.30
48.70
40.30 DMSO
40.09 DMSO
39.67 DMSO
39.46 DMSO
39.25 DMSO
39.05 DMSO
37.18
35.52
35.45
35.32
34.80
30.99
30.81
28.98
23.13
21.56
19.50
18.37
8.11
210 200 190 180 170 160 150 140 130 120 110 100 90 80 70 60 50 40 30 20 10 0 -10
f1 (ppm)

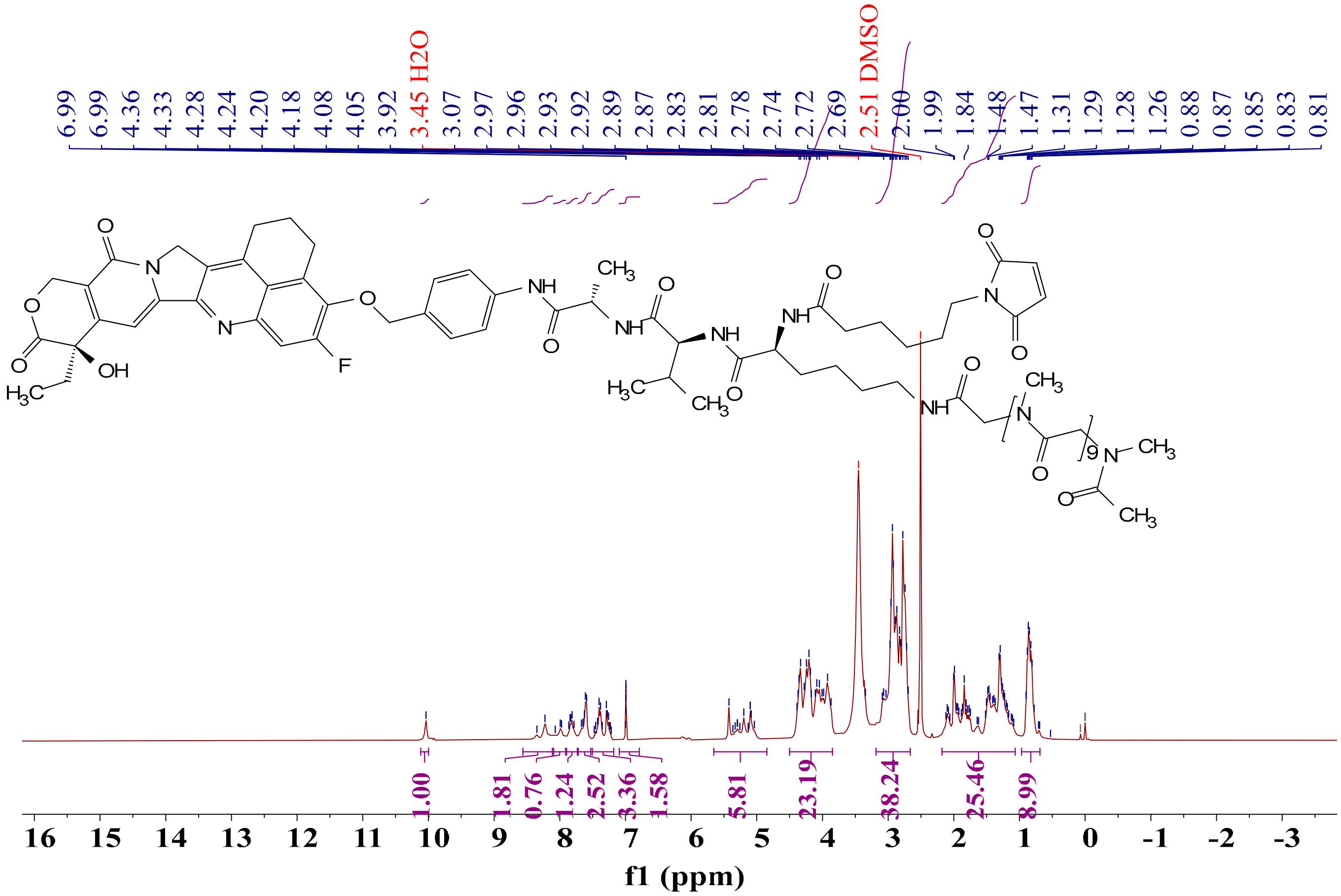
6.99
6.99
4.36
4.33
4.28
4.24
4.20
4.18
4.08
4.05
3.92
3.45 H2O
3.07
2.97
2.96
2.93
2.92
2.89
2.87
2.83
2.81
2.78
2.74
2.72
2.69
2.51 DMSO
2.00
1.99
1.84
1.48
1.47
1.31
1.29
1.28
1.26
0.88
0.87
0.85
0.83
0.81
1.00
1.81
0.76
1.24
2.52
3.36
1.58
5.81
23.19
38.24
25.46
8.99
16 15 14 13 12 11 10 9 8 7 6 5 4 3 2 1 0 -1 -2 -3
f1 (ppm)

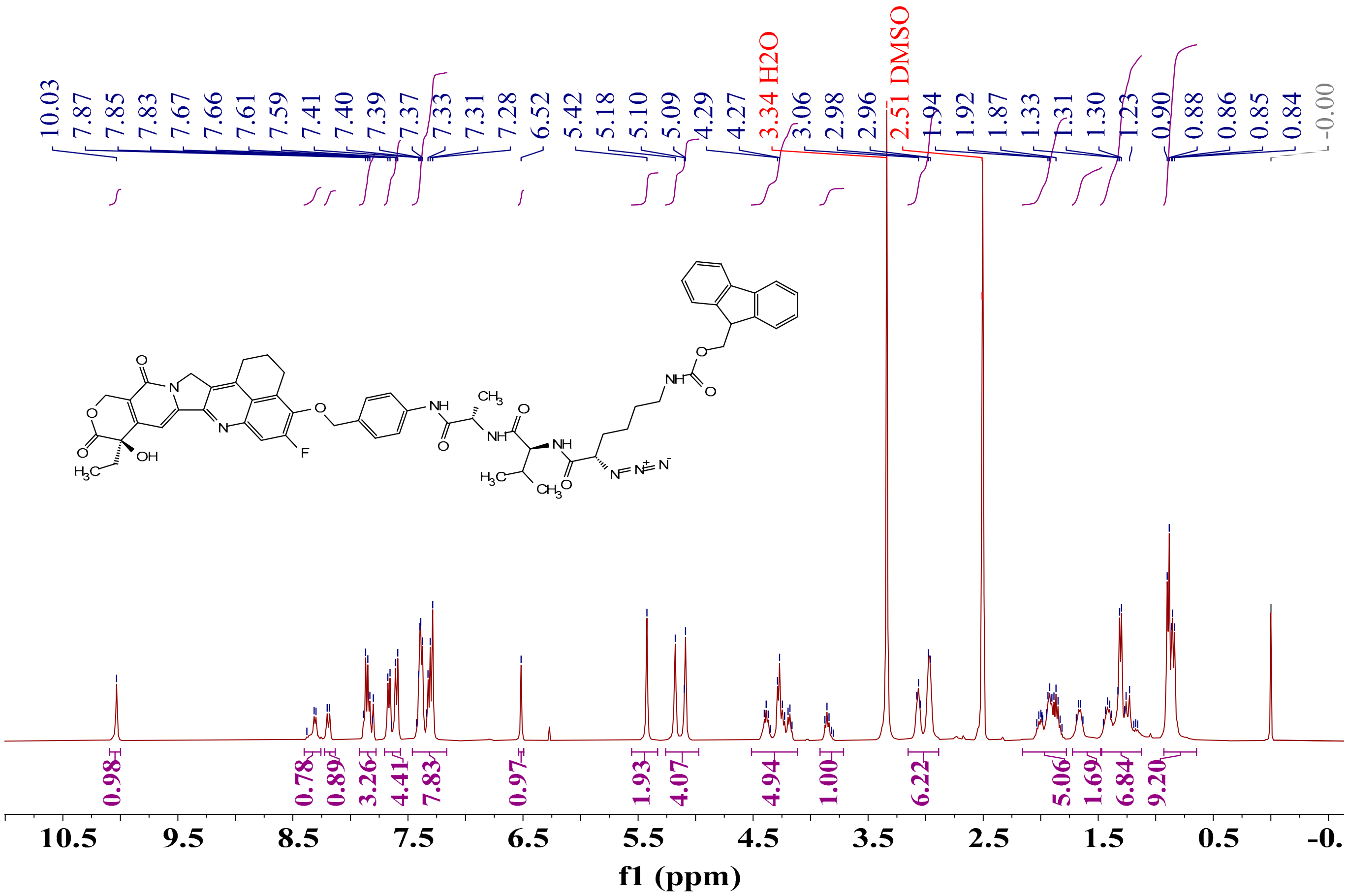
10.03 7.87 7.85 7.83 7.67 7.66 7.61 7.59 7.41 7.40 7.39 7.37 7.33 7.31 7.28 6.52 5.42 5.18 5.10 5.09 4.29 4.27
3.34 H2O
3.06 2.98 2.96
2.51 DMSO
1.94 1.92 1.87 1.33 1.31 1.30 1.23 0.90 0.88 0.86 0.85 0.84 -0.00
0.98 0.78 0.89 3.26 4.41 7.83 0.97 1.93 4.07 4.94 1.00 6.22 5.06 1.69 6.84 9.20
10.5 9.5 8.5 7.5 6.5 5.5 4.5 3.5 2.5 1.5 0.5 -0.
f1 (ppm)

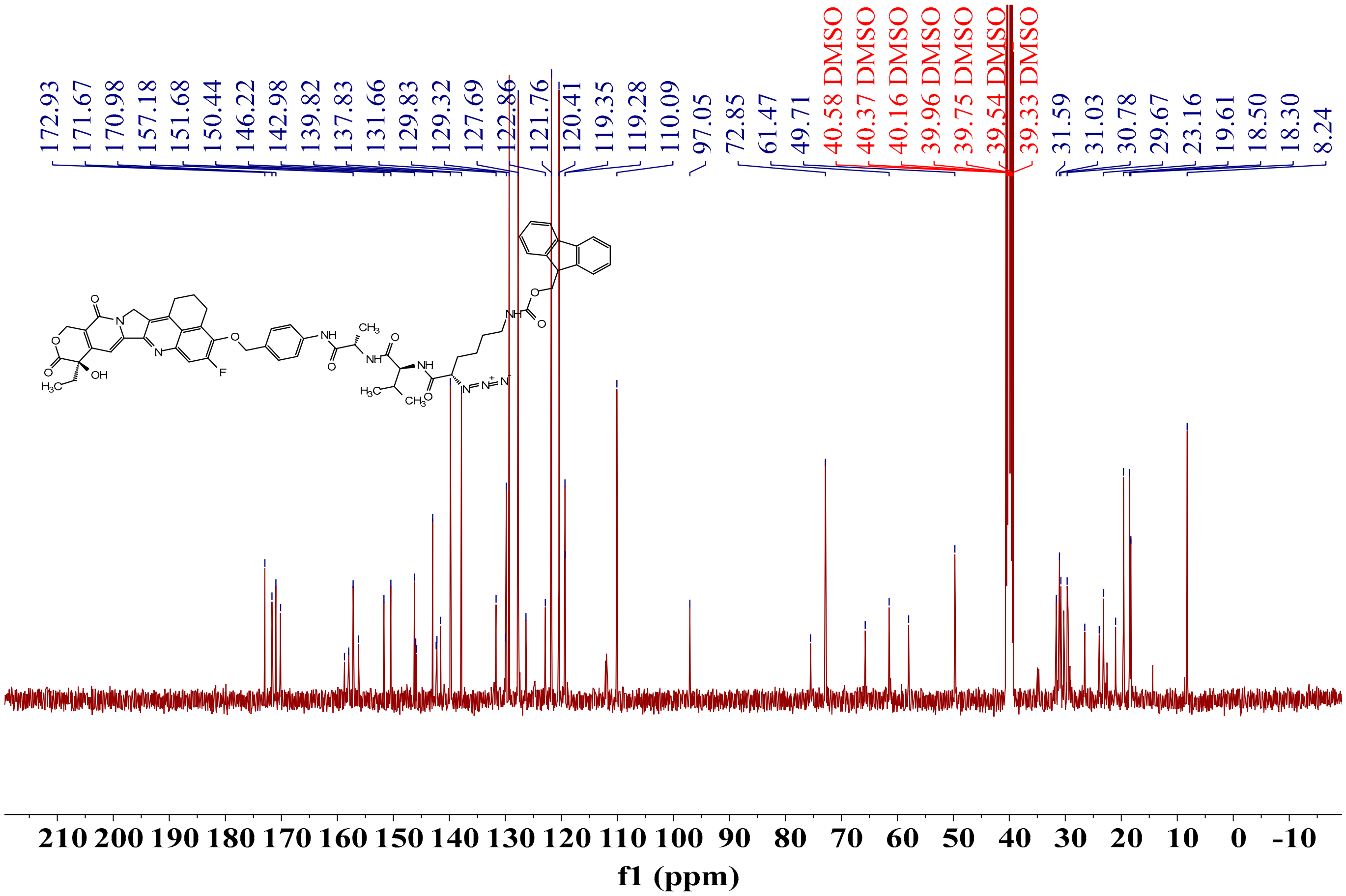
172.93 171.67 170.98 157.18 151.68 150.44 146.22 142.98 139.82 137.83 131.66 129.83 129.32 127.69 122.86 121.76 120.41 119.35 119.28 110.09 97.05 72.85 61.47 49.71
40.58 DMSO
40.37 DMSO
40.16 DMSO
39.96 DMSO
39.75 DMSO
39.54 DMSO
39.33 DMSO
31.59 31.03 30.78 29.67 23.16 19.61 18.50 18.30 8.24
210 200 190 180 170 160 150 140 130 120 110 100 90 80 70 60 50 40 30 20 10 0 -10
f1 (ppm)

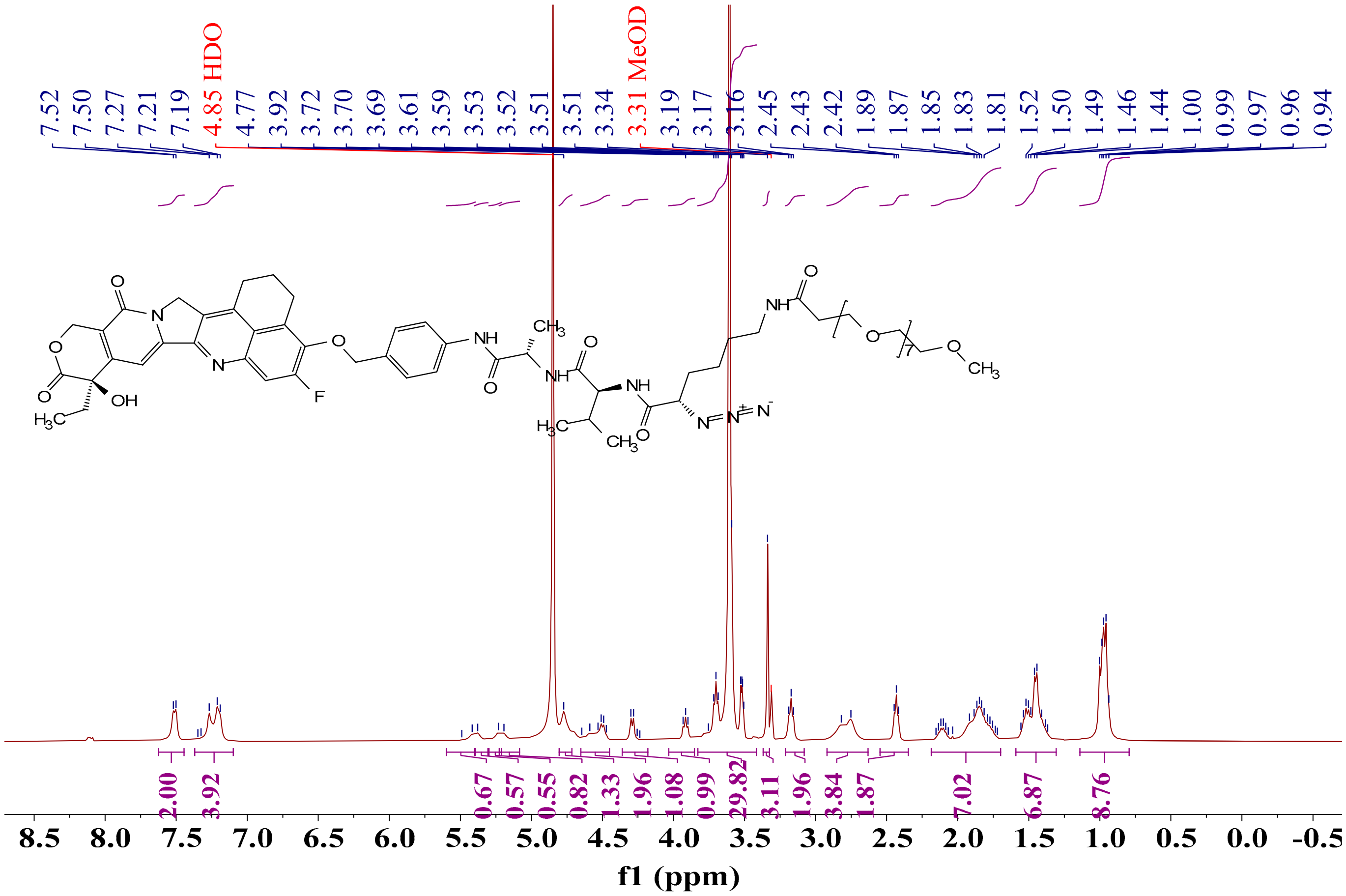
7.52 7.50 7.27 7.21 7.19 4.85 HDO 4.77 3.92 3.72 3.70 3.69 3.61 3.59 3.53 3.52 3.51 3.51 3.34 3.31 MeOD 3.19 3.17 3.16 2.45 2.43 2.42 1.89 1.87 1.85 1.83 1.81 1.52 1.50 1.49 1.46 1.44 1.00 0.99 0.97 0.96 0.94
2.00 3.92 0.67 0.57 0.55 0.82 1.33 1.96 1.08 0.99 29.82 3.11 1.96 3.84 1.87 7.02 6.87 8.76
f1 (ppm)

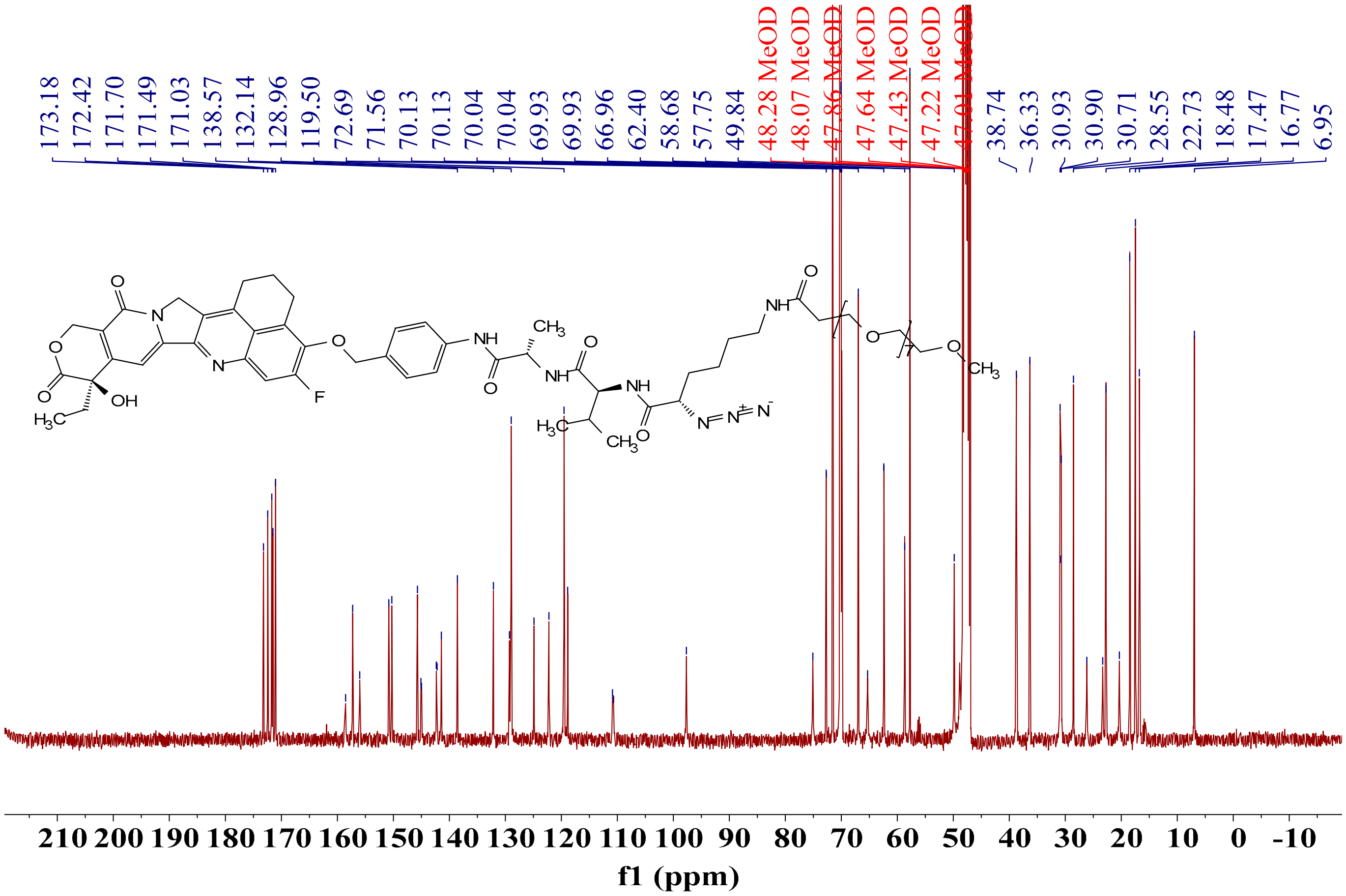
173.18 172.42 171.70 171.49 171.03 138.57 132.14 128.96 119.50 72.69 71.56 70.13 70.13 70.04 70.04 69.93 69.93 66.96 62.40 58.68 57.75 49.84 48.28 MeOD 48.07 MeOD 47.86 MeOD 47.64 MeOD 47.43 MeOD 47.22 MeOD 47.01 MeOD 38.74 36.33 30.93 30.90 30.71 28.55 22.73 18.48 17.47 16.77 6.95
f1 (ppm)

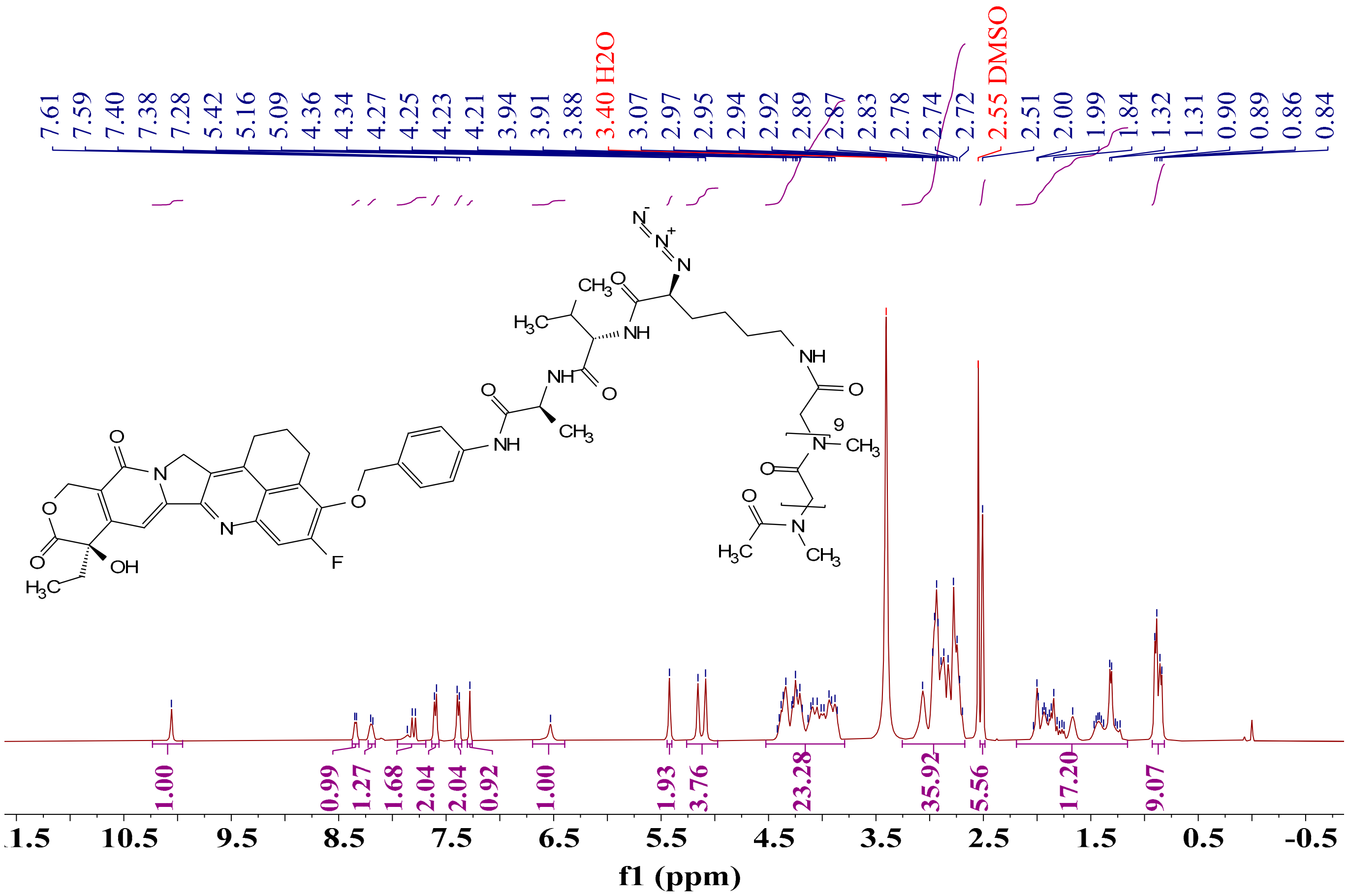
7.61 7.59 7.40 7.38 7.28 5.42 5.16 5.09 4.36 4.34 4.27 4.25 4.23 4.21 3.94 3.91 3.88 3.40 H2O 3.07 2.97 2.95 2.94 2.92 2.89 2.87 2.83 2.78 2.74 2.72 2.55 DMSO 2.51 2.00 1.99 1.84 1.32 1.31 0.90 0.89 0.86 0.84
1.00 0.99 1.27 1.68 2.04 2.04 0.92 1.00 1.93 3.76 23.28 35.92 5.56 17.20 9.07
f1 (ppm)

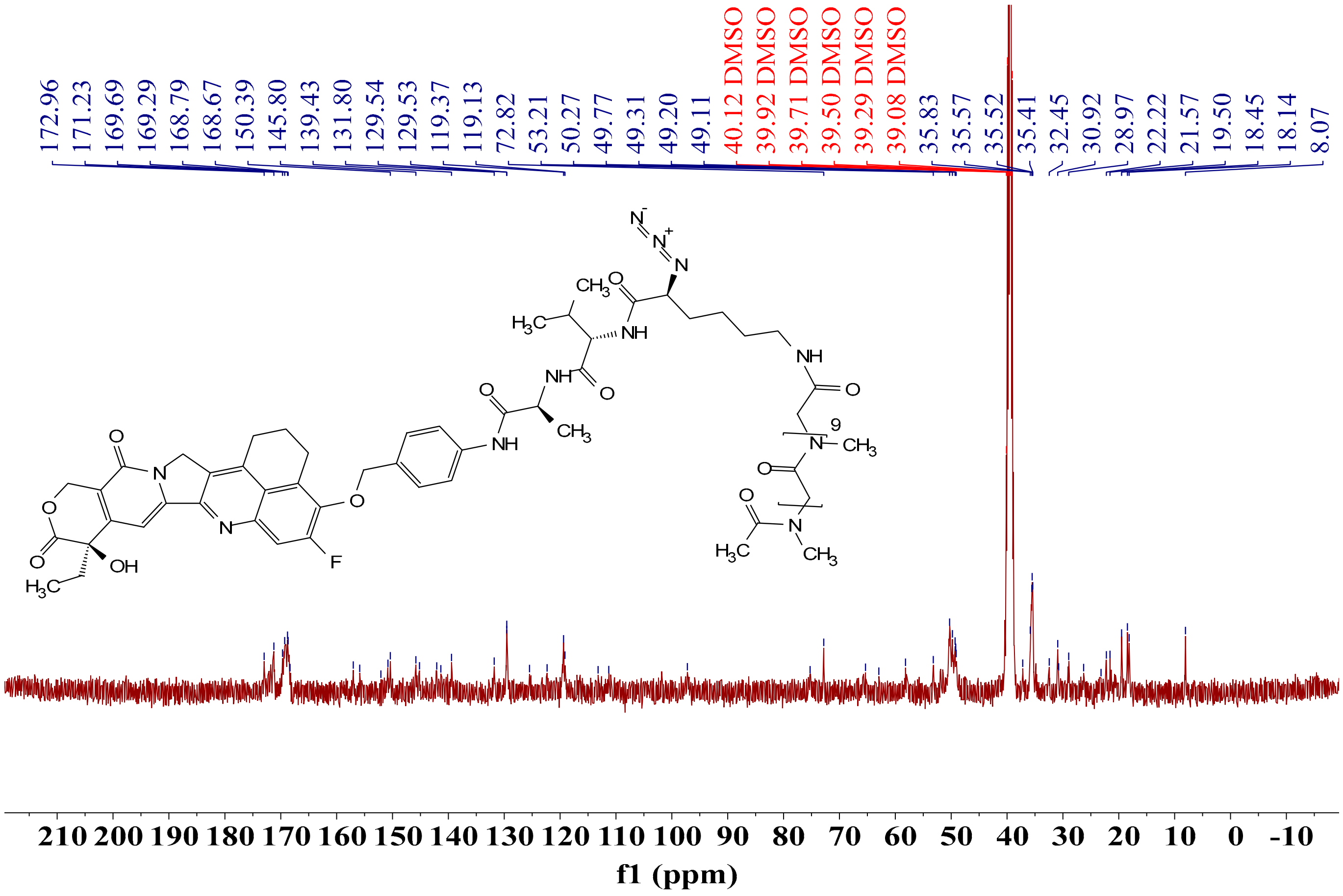
172.96 171.23 169.69 169.29 168.79 168.67 150.39 145.80 139.43 131.80 129.54 129.53 119.37 119.13 72.82 53.21 50.27 49.77 49.31 49.20 49.11 40.12 DMSO 39.92 DMSO 39.71 DMSO 39.50 DMSO 39.29 DMSO 39.08 DMSO 35.83 35.57 35.52 35.41 32.45 30.92 28.97 22.22 21.57 19.50 18.45 18.14 8.07
f1 (ppm)

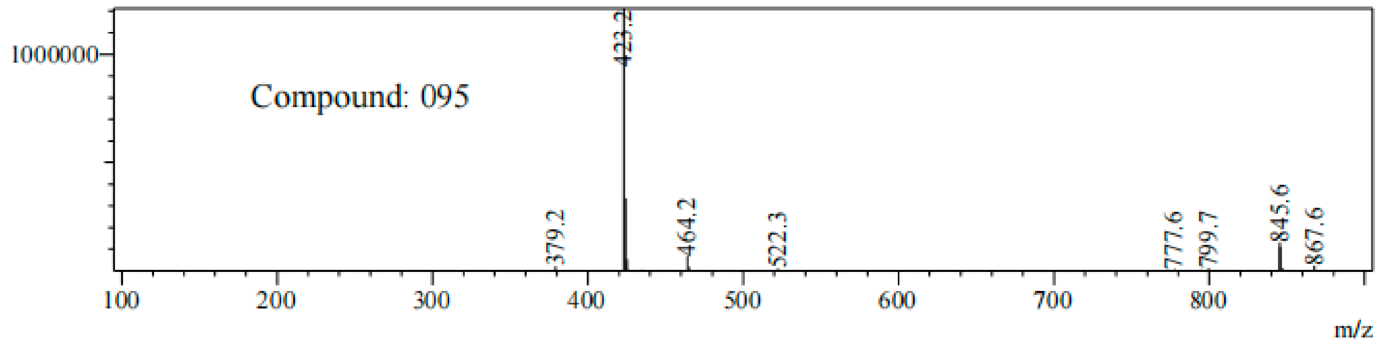
Compound: 095
1000000
423.2
379.2
464.2
522.3
777.6
799.7
845.6
867.6
m/z

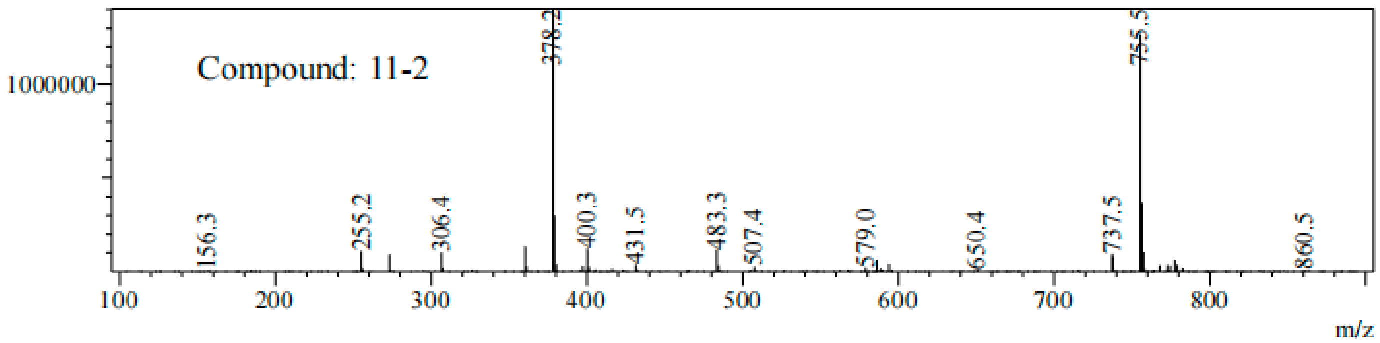
Compound: 11-2
1000000
378.2
755.5
156.3
255.2
306.4
400.3
431.5
483.3
507.4
579.0
650.4
737.5
860.5
m/z

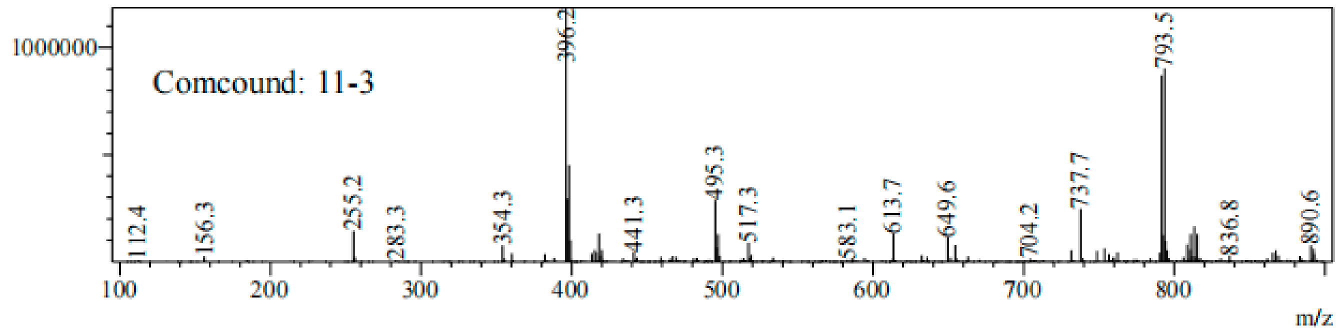
Comcound: 11-3
1000000
396.2
793.5
112.4
156.3
255.2
283.3
354.3
441.3
495.3
517.3
583.1
613.7
649.6
704.2
737.7
836.8
890.6
m/z

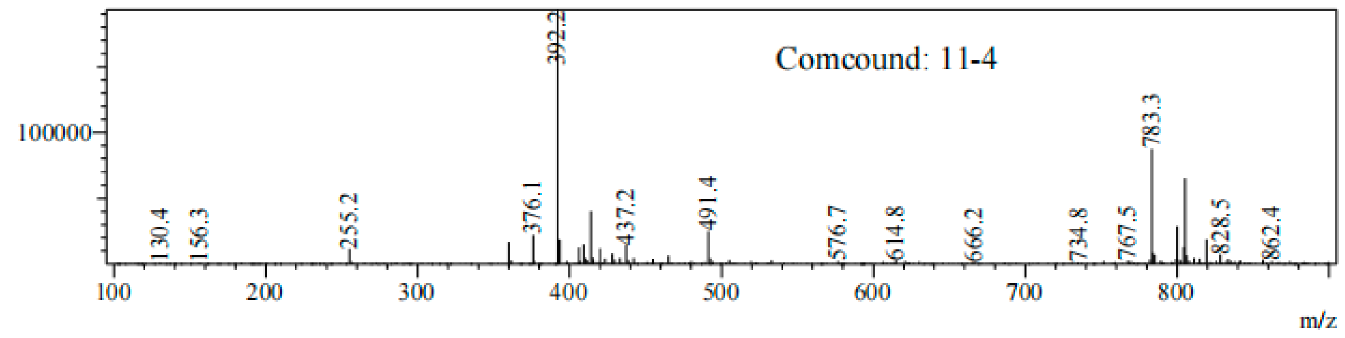
Comcound: 11-4
100000
392.2
783.3
130.4
156.3
255.2
376.1
437.2
491.4
576.7
614.8
666.2
734.8
767.5
828.5
862.4
m/z

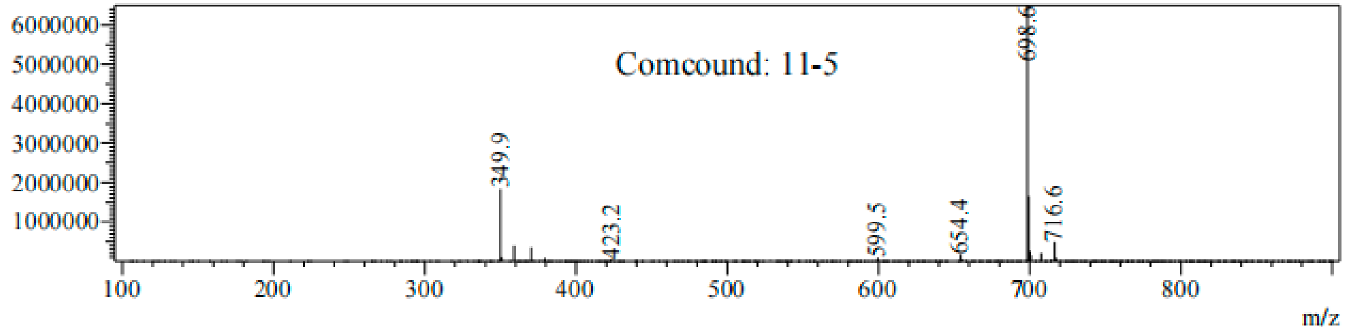
Comcound: 11-5
6000000
5000000
4000000
3000000
2000000
1000000
349.9
423.2
599.5
654.4
698.6
716.6
100
200
300
400
500
600
700
800
m/z

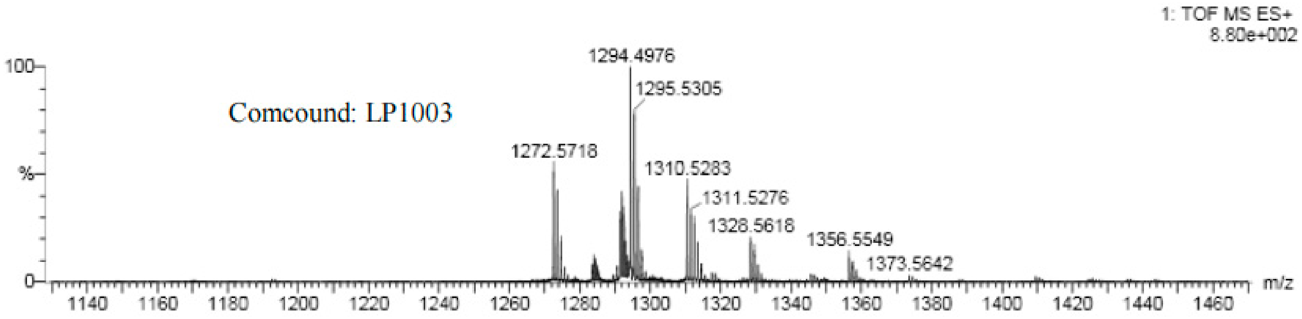
1: TOF MS ES+
8.80e+002
100
%
0
Comcound: LP1003
1294.4976
1295.5305
1272.5718
1310.5283
1311.5276
1328.5618
1356.5549
1373.5642
1140
1160
1180
1200
1220
1240
1260
1280
1300
1320
1340
1360
1380
1400
1420
1440
1460
m/z

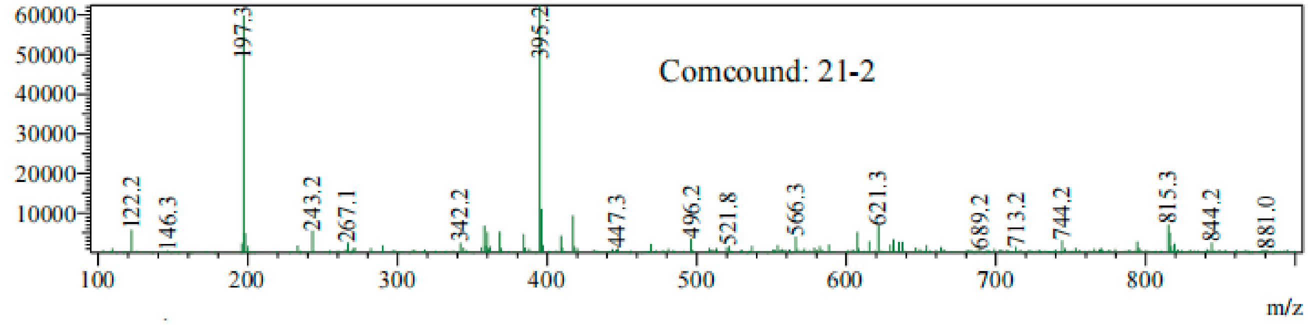
Comcound: 21-2
60000
50000
40000
30000
20000
10000
122.2
146.3
197.3
243.2
267.1
342.2
395.2
447.3
496.2
521.8
566.3
621.3
689.2
713.2
744.2
815.3
844.2
881.0
100
200
300
400
500
600
700
800
m/z

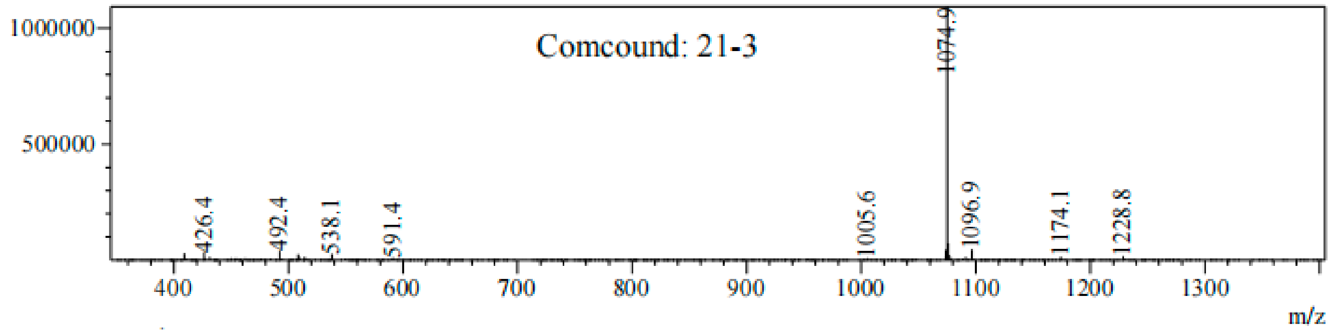
Comcound: 21-3
1000000
500000
426.4
492.4
538.1
591.4
1005.6
1074.9
1096.9
1174.1
1228.8
400
500
600
700
800
900
1000
1100
1200
1300
m/z

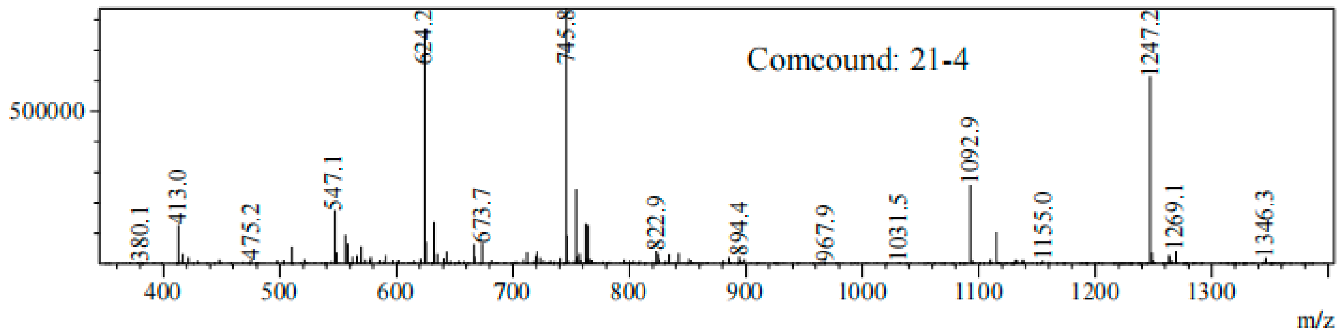
Comcound: 21-4
500000
380.1
413.0
475.2
547.1
624.2
673.7
745.8
822.9
894.4
967.9
1031.5
1092.9
1155.0
1247.2
1269.1
1346.3
400
500
600
700
800
900
1000
1100
1200
1300
m/z

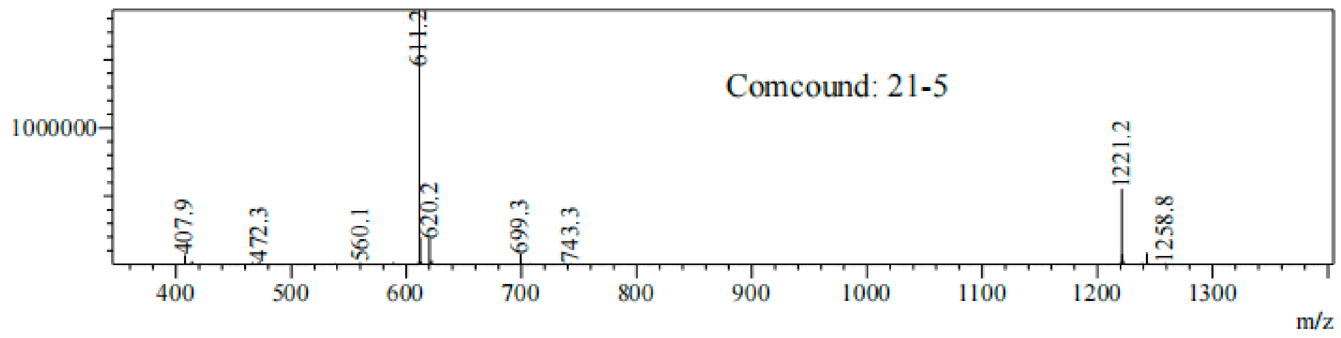
Comcound: 21-5
1000000
611.2
407.9
472.3
560.1
620.2
699.3
743.3
1221.2
1258.8
400
500
600
700
800
900
1000
1100
1200
1300
m/z

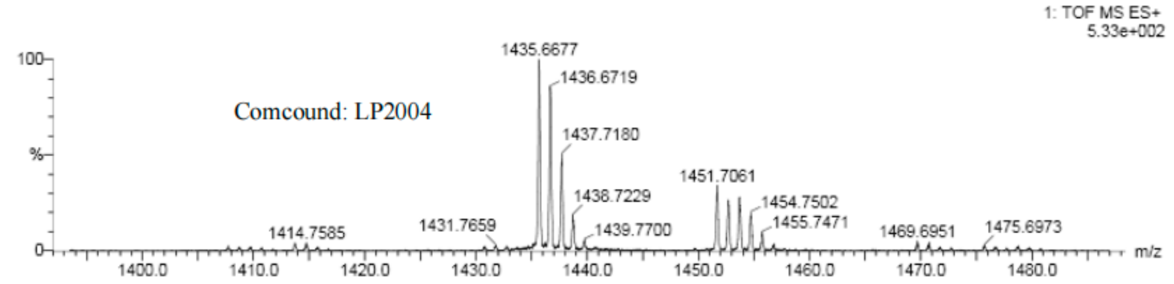
1: TOF MS ES+
5.33e+002
100
%
0
Comcound: LP2004
1435.6677
1436.6719
1437.7180
1438.7229
1439.7700
1451.7061
1454.7502
1455.7471
1414.7585
1431.7659
1469.6951
1475.6973
1400.0
1410.0
1420.0
1430.0
1440.0
1450.0
1460.0
1470.0
1480.0
m/z

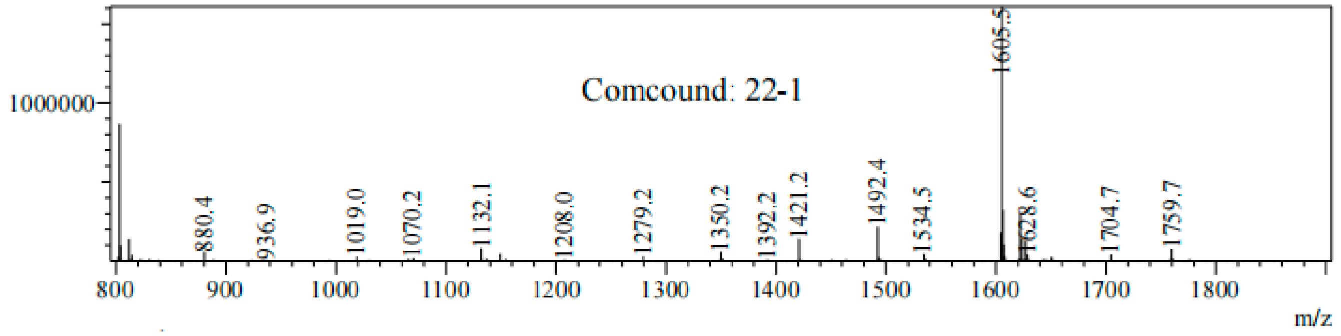
Comcound: 22-1
1000000
1605.5
880.4
936.9
1019.0
1070.2
1132.1
1208.0
1279.2
1350.2
1392.2
1421.2
1492.4
1534.5
1628.6
1704.7
1759.7
800
900
1000
1100
1200
1300
1400
1500
1600
1700
1800
m/z

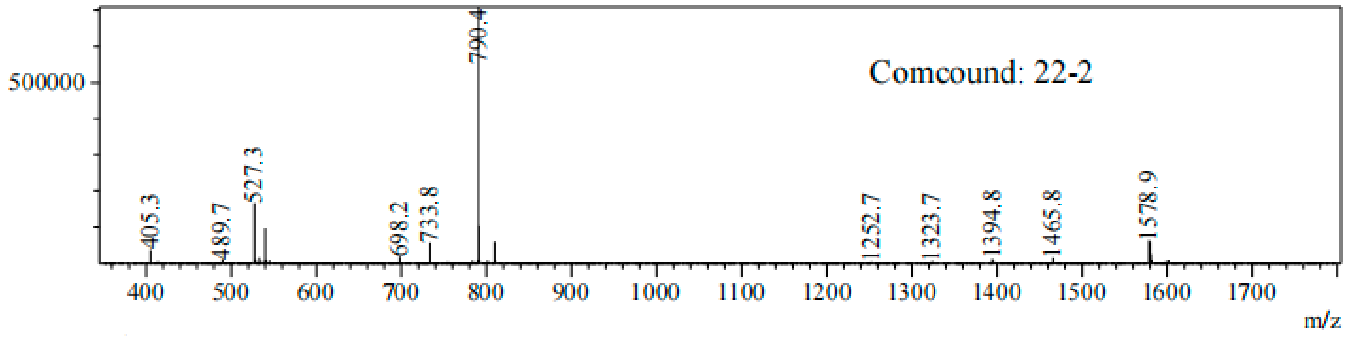
Comcound: 22-2
500000
790.4
405.3
489.7
527.3
698.2
733.8
1252.7
1323.7
1394.8
1465.8
1578.9
400
500
600
700
800
900
1000
1100
1200
1300
1400
1500
1600
1700
m/z

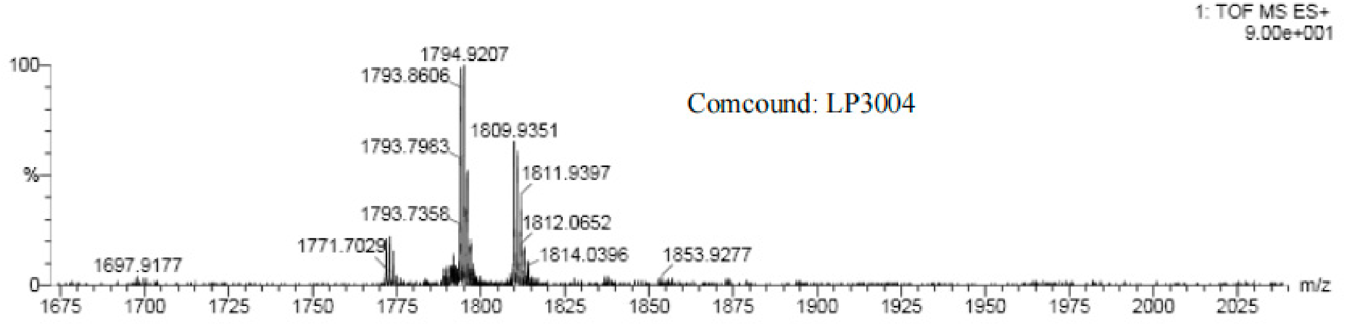
1: TOF MS ES+
9.00e+001
100
%
0
Comcound: LP3004
1794.9207
1793.8606
1809.9351
1793.7963
1811.9397
1793.7358
1812.0652
1771.7029
1814.0396
1853.9277
1697.9177
1675
1700
1725
1750
1775
1800
1825
1850
1875
1900
1925
1950
1975
2000
2025
m/z
